# Proteomic Profiling Reveals Novel Molecular Insights into Dysregulated Proteins in Established Cases of Rheumatoid Arthritis

**DOI:** 10.3390/proteomes13030032

**Published:** 2025-07-04

**Authors:** Afshan Masood, Hicham Benabdelkamel, Assim A. Alfadda, Abdurhman S. Alarfaj, Amina Fallata, Salini Scaria Joy, Maha Al Mogren, Anas M. Abdel Rahman, Mohamed Siaj

**Affiliations:** 1 Proteomics Resource Unit, Obesity Research Center, College of Medicine, King Saud University, Riyadh 11461, Saudi Arabia; afsmasood@ksu.edu.sa (A.M.); helkamel@ksu.edu.sa (H.B.); aalfadda@ksu.edu.sa (A.A.A.); afallata@ksu.edu.sa (A.F.); 2 Department of Chemistry and Biochemistry, Université du Québec à Montréal (UQAM), Montreal, QC H2L 2C4, Canada; 3 Department of Medicine, College of Medicine, King Saud University, Riyadh 11461, Saudi Arabia; 4 Strategic Center for Diabetes Research, College of Medicine, King Saud University, Riyadh 11461, Saudi Arabia; sjoy@ksu.edu.sa; 5 Rheumatology Unit, Department of Medicine, College of Medicine, King Saud University, Riyadh 11461, Saudi Arabia; asarfaj@ksu.edu.sa; 6 Metabolomics Section, Precision Medicine Laboratory Department, Genome Medicine Center of Excellence, King Faisal Specialist Hospital and Research Centre (KFSHRC), Riyadh 11211, Saudi Arabia; mmogren@kfshrc.edu.sa; 7 Department of Biochemistry and Molecular Medicine, College of Medicine, Alfaisal University, Riyadh 11533, Saudi Arabia

**Keywords:** rheumatoid arthritis, plasma proteomics, osteocalcin, metallothionein-2, 2D DIGE, MALDI-TOF, biomarker

## Abstract

**Background**: Rheumatoid arthritis (RA) is a chronic autoimmune disorder that predominantly affects synovial joints, leading to inflammation, pain, and progressive joint damage. Despite therapeutic advancements, the molecular basis of established RA remains poorly defined. **Methods:** In this study, we conducted an untargeted plasma proteomic analysis using two-dimensional differential gel electrophoresis (2D-DIGE) and matrix-assisted laser desorption/ionization time-of-flight mass spectrometry (MALDI-TOF-MS) in samples from RA patients and healthy controls in the discovery phase. **Results:** Significantly (ANOVA, *p* ≤ 0.05, fold change > 1.5) differentially abundant proteins (DAPs) were identified. Notably, upregulated proteins included mitochondrial dicarboxylate carrier, hemopexin, and 28S ribosomal protein S18c, while CCDC124, osteocalcin, apolipoproteins A-I and A-IV, and haptoglobin were downregulated. Receiver operating characteristic (ROC) analysis identified CCDC124, osteocalcin, and metallothionein-2 with high diagnostic potential (AUC = 0.98). Proteins with the highest selected frequency were quantitatively verified by multiple reaction monitoring (MRM) analysis in the validation cohort. Bioinformatic analysis using Ingenuity Pathway Analysis (IPA) revealed the underlying molecular pathways and key interaction networks involved STAT1, TNF, and CD40. These central nodes were associated with immune regulation, cell-to-cell signaling, and hematological system development. **Conclusions:** Our combined proteomic and bioinformatic approaches underscore the involvement of dysregulated immune pathways in RA pathogenesis and highlight potential diagnostic biomarkers. The utility of these markers needs to be evaluated in further studies and in a larger cohort of patients.

## 1. Introduction

Rheumatoid arthritis (RA) is a complex and challenging disease characterized by a chronic autoimmune inflammatory state mainly involving the synovial joints, leading to articular damage, pain, and disability. The global prevalence of RA has been estimated to affect approximately 0.1–2.0% of the population, affecting females more than their male counterparts [1]. The etiology of RA is multifactorial, arising from complex interactions between genetic predisposition, immune dysregulation, and environmental triggers. Its development is strongly influenced by T cells and genetic susceptibility linked to the major histocompatibility complex (MHC), particularly HLA class II genes. Polymorphisms within the HLA-DR, DP, and DQ subregions—especially the HLA-DRB1 locus—have been consistently associated with increased RA susceptibility [2]. The onset of RA is hypothesized to involve dysregulation of immune signaling pathways, leading to heightened local and systemic inflammation. To this effect, immune cells of both the innate and adaptive immune systems, including monocytes, macrophages, as well as the B and T lymphocytes acting through several mediators, have been implicated in the pathogenesis of RA [3].

Patients with RA generally present clinically with swelling and pain in the joints that, in severe cases, result in the erosion of bones and deformity of the joints [4]. This can be accompanied by complications such as anemia, osteoporosis, cardiovascular disease, and lung disorders [5] in prolonged cases of the disease. The early diagnosis and treatment of RA can ameliorate these conditions, but many reports show that the underlying pathology is not resolved. Routine laboratory diagnosis of RA is primarily based on assessing disease activity through detecting high levels of autoantibodies such as rheumatoid factor, anti-citrullinated protein antibodies (ACPAs), and anti-carbamylated protein antibodies that have been used, although with low sensitivity and limited reliability [5,6].

Proteomics, a powerful branch of molecular biology, has emerged as a promising tool for investigating the complex and dynamic changes occurring within cells and tissues during disease through a systematic study of the entire proteome. The high-throughput nature of proteomic technologies allows for the simultaneous assessment of numerous proteins, making it well-suited for the comprehensive profiling of the molecular alterations associated with RA. Proteomics approaches have used different biological fluids, including serum, plasma, and synovial fluid, to elucidate the molecular heterogeneity of RA to study markers for the early diagnosis of disease [7] and to identify those that can indicate remissions and avoid disease flare-ups [8,9,10]. In recent years, significant progress has been made in RA proteomic research aiming to yield novel biomarkers and potential therapeutic targets and in ascertaining potential pathways involved in RA pathogenesis [11,12,13,14]. Recently, two studies by Hu et al. and Cheng et al. using proteomics proposed that changes in the serum levels of ORM1 and FCN-2, respectively, were correlated with disease activity [12,15]. Proteomic analysis of the synovial fluid by Liao et al. showed changes in S100A9 and S100A12 proteins as prognostic markers of erosive forms of RA [16]. Recently, a targeted proteomic analysis of plasma samples using LC-MS/MS in MRM mode was carried out to accurately quantify soluble co-stimulatory molecules as potential RA diagnostic biomarkers [17].

Despite the significant steps towards understanding RA, the underlying pathophysiology and molecular mechanisms underlying disease remain elusive. In the present study, we aimed to use 2D-DIGE coupled with MALDI-TOF-MS to identify changes in the plasma proteome between the RA and control groups and relate them to the affected metabolic pathways using bioinformatics analysis. Exploring the untargeted plasma proteome will provide the ability to gain a better perception of changes in molecular pathways and provide potential biomarkers for disease diagnosis, prognosis, or management.

## 2. Results

### 2.1. Clinical and Biochemical Data for the Study Participants

The demographic and biochemical characteristics of the study participants in the discovery and validation phases are presented in Table 1. The mean ages of the study participants in the RA and control groups were 46.8 ± 12.4 and 41.5 ± 6.2 years, respectively, and were matched in body weight, BMI, and other laboratory markers. Significant differences were noted in the ESR and CRP levels, which were higher in the RA group than in the control group.

### 2.2. Proteomic Analysis and Identification of Differentially Expressed Proteins

**Figure 1:**
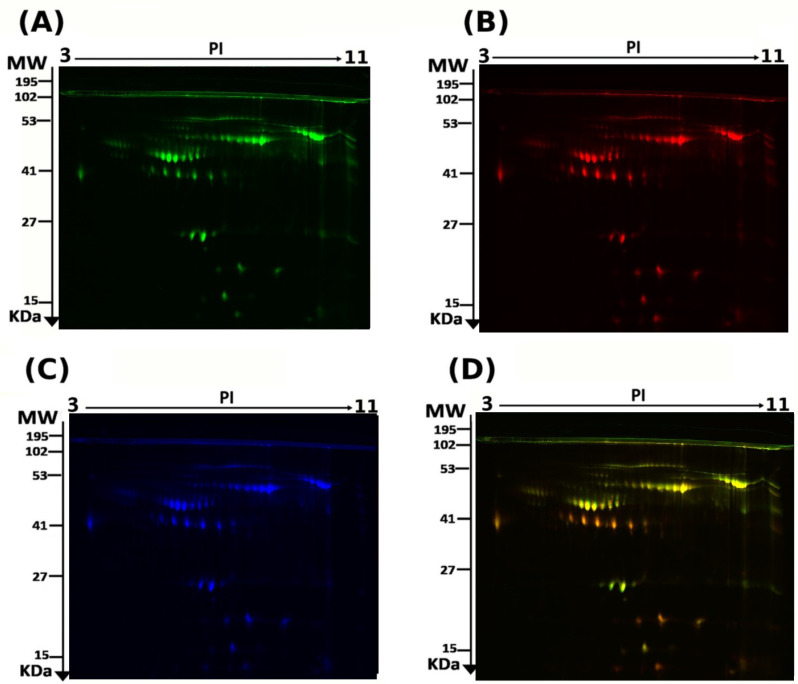
Representative images of two-dimensional difference in gel electrophoresis (2D-DIGE) gels depicting the separation of the fluorescent cye-dye-labeled proteins. RA samples labeled with Cy3 (**A**), controls labeled with Cy5 (**B**), pooled internal controls labeled with Cy2 (**C**), and the merged image (**D**).

The DAPs spots between the RA and control groups (24 samples from 12 gels) were assessed using 2D-DIGE coupled with MALDI-TOF mass spectrometry. Figure 1 shows the representative fluorescent protein profiles of the 2D-DIGE of control samples labeled with Cy5 (Figure 1A), RA samples labeled with Cy3 (Figure 1B), and a pooled internal control labeled with Cy2 (Figure 1D). A total of 1200 spots were detected, matching across all 24 gel images. These spot patterns were reproducible across all 24 gel images, leading to alignment and further analysis. Normalization across the complete gels and quantitative differential analysis of the protein levels were achieved using an internal standard with Cy2 labeling. Figure S1 shows a total of 89 spots that were statistically significant on the gels (cutoff: ANOVA, FDR *p* ≤ 0.05; fold change of 1.5) between the RA and control groups.

**Figure 2:**
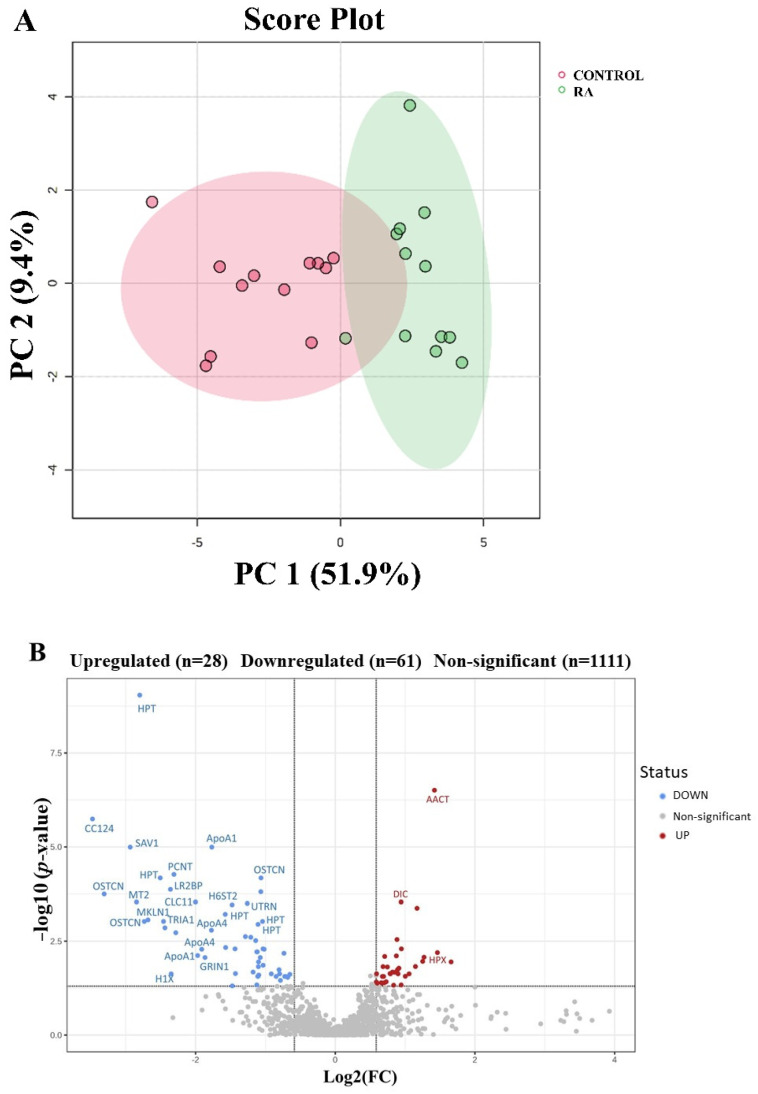
Unsupervised univariate analysis of the DAPs identified between RA and control groups. (**A**) Principal component analysis of the proteomic dataset demonstrating the separation between the RA and control groups. The green dots indicate the RA patients, and the red dots represent the controls. The PC1 components explained 41.9% of the selected spot’s variability values. The colored dots and numbers represent the gels and spots, respectively. (**B**) The volcano plot represents the gene names corresponding to the protein spots between the RA and control groups, which revealed significant dysregulation in 89 protein spots based on the cutoffs (cutoff: ANOVA, *p*-value ≤ 0.05, and FC ≥ 1.5). From these, 28 (red) and 61 (blue) protein spots were upregulated and downregulated, respectively. The gray circled (Non-significant) protein spots (1111) failed to pass both cutoffs and were excluded from further analysis.

An additional univariate analysis using the MetaboAnalyst software package (MetaboAnalyst version 6.0, McGill University, Montreal, QC, Canada; http://www.metaboanalyst.ca, access date 10 September 2024) was conducted, where the data were further normalized via log transformation and Pareto scaling. The significant and dysregulated proteins in this study were further analyzed using PCA (Figure 2A) and a volcano plot, in which the x- and y-axis indicate the fold change (cutoff > 1.5) and the y-axis *t*-test (FDR *p* < 0.05), respectively (Figure 2B). This rigorous analysis combining the expression fold change and FDR-corrected *p*-value (FDRp) showed that only 28 and 61 proteins were up- and downregulated in the RA patients compared with the control patients, respectively. The spots showing a statistical significance between the two conditions were then manually excised from the preparative gel for protein identification by MALDI TOF-MS.

### 2.3. Mass Spectrometry Analysis and Protein Identification Using MALDI-TOF

Peptide mass fingerprints (PMFs) successfully identified 69 of the 89 protein spots excised from the preparative gel that were successfully matched to entries in the SWISS-PROT database by Mascot with high confidence scores. The sequence coverage of the proteins identified by PMF ranged from 8 to 75% (Table S1). In a few cases, variants of the same protein were found at several locations on the gel (Table S2, Figure S1). Among the differentially abundant proteins (DAPs) identified in RA vs. controls, 12 proteins demonstrated an increased abundance, while 57 had a decreased abundance (Table S2). The proteins with significantly increased abundance included mitochondrial dicarboxylate carrier (1.59-fold, *p* < 0.001), hemopexin (1.9-fold, *p* < 0.001), 28S ribosomal protein S18c (mitochondrial) (1.58-fold, *p* = 0.017), coiled-coil domain-containing protein 151 (2.87-fold, *p* = 0.022), and nucleolar and spindle-associated protein 1 (1.65-fold, *p* = 0.01); the full list is provided in Table S2. The significantly DAPs with decreased abundance included coiled-coil domain-containing protein 124 (1.5-fold, <0.001), osteocalcin (2.87-fold, <0.001), apolipoprotein A-I (2.87-fold, <0.001), apolipoprotein A-IV (2.87-fold, <0.001), haptoglobin (HPT) (2.87-fold, *p* = 0.001), muskelin (1.92-fold, <0.001), heparan-sulfate 6-O-sulfotransferase 2 (1.6-fold, *p* = 0.002), branched-chain-amino-acid aminotransferase (1.66-fold, *p* = 0.029), and mitochondrial metallothionein-2 (1.5-fold, <0.001), with the full list presented in Table S2. Several proteins, 27 in all, including osteocalcin, haptoglobin, alpha-1-antitrypsin, and albumin, were found in more than one spot on the gels and appeared as multiple proteoforms with shifts in both pI or MW, which could be associated with their post-translational modifications for example (glycosylation and phosphorylation) or degradation, cleavage by enzymes (Table S2).

### 2.4. Orthogonal Partial Least Squares-Discriminant Analysis and Heat Map for the Proteomics Profile Between RA and Control Groups

**Figure 3:**
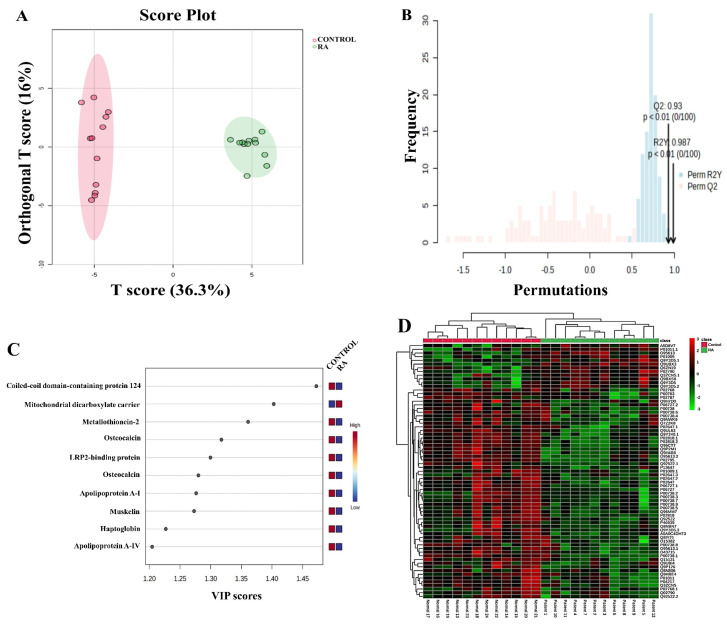
Proteomics profiling between the RA and control groups. (**A**) The orthogonal partial least squares-discriminant analysis (OPLS-DA) score plot showed evident separation between the two groups (RA and controls). The control and RA samples are represented as red and green circles, respectively. (**B**) The robustness of the created models was evaluated by the fitness of the model (R2Y = 0.987) and predictive ability (Q2 = 0.93) values. (**C**) The top 10 features of variable importance identified between the RA and control groups. (**D**) The heat map and hierarchical clustering analysis of the 69 identified proteins that were significantly altered between the RA and control groups based on the Pearson measure and average clustering method with the *t*-test/ANOVA using MetaboAnalyst Software V6 (Montreal, QC, Canada).

A multivariate supervised analysis using the OPLS-DA model was used to analyze the DAPs identified between RA and control groups. The OPLS-DA showed a clear and distinct separation between the two groups, highlighting that the proteomic profile of patients with RA significantly differed from that of the control group. Model robustness was assessed through a permutation-based approach involving 100 iterations using MetboAnalyst that yielded an R2Y value of 0.987 and a Q2 value of 0.93, reflecting excellent fit and predictive capability (Figure 3A,B). The top 10 discriminant proteins with a variable influence on projection (VIP) value of >1 were responsible for the proposed separation (Figure 3C). A heat map and hierarchical clustering analysis of the differentially abundant proteins identified via MALDI-TOF-MS showed clusters of different expression patterns (Figure 3D) between the two groups. The clustering pattern demonstrates that the changes in the protein intensity of selected spots in RA compared to the control samples were significantly different (Figure 3D). Shades of red indicate high levels of expression, and shades of green indicate low levels of expression.

### 2.5. Functional Enrichment of DAPs in RA Group Compared to Control Group

**Figure 4:**
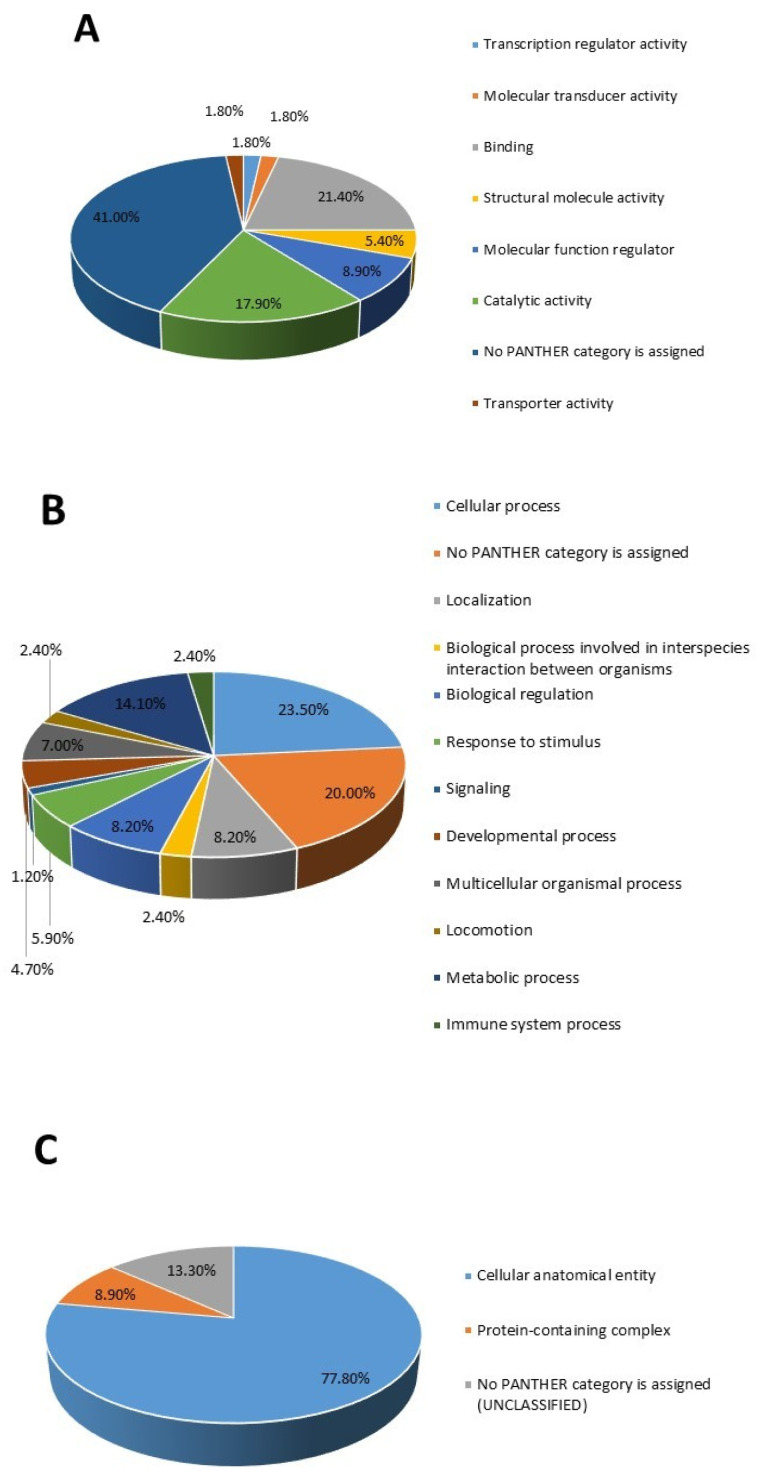
Pie charts showing the protein classes based on (**A**) molecular function, (**B**) biological process, and (**C**) cellular component of the significantly differentially abundant proteins between the RA and control groups.

The DAPs were classified using the Protein Analysis Through Evolutionary Relationships (PANTHER) system into their molecular and biological functions and cellular components (Figure 4). Gene ontology analysis showed that the differentially expressed proteins were primarily associated with binding and catalytic activity as their main molecular functions (Figure 4A). These proteins were involved in cellular and metabolic processes and biological regulation and localized mainly within cellular anatomical structures and protein-containing complexes with corresponding FDR-adjusted *p*-values presented in Table S3; Figure 4B,C).

### 2.6. Biomarker and Network Pathway Analysis of the DAPs Using Bioinformatics

**Figure 5:**
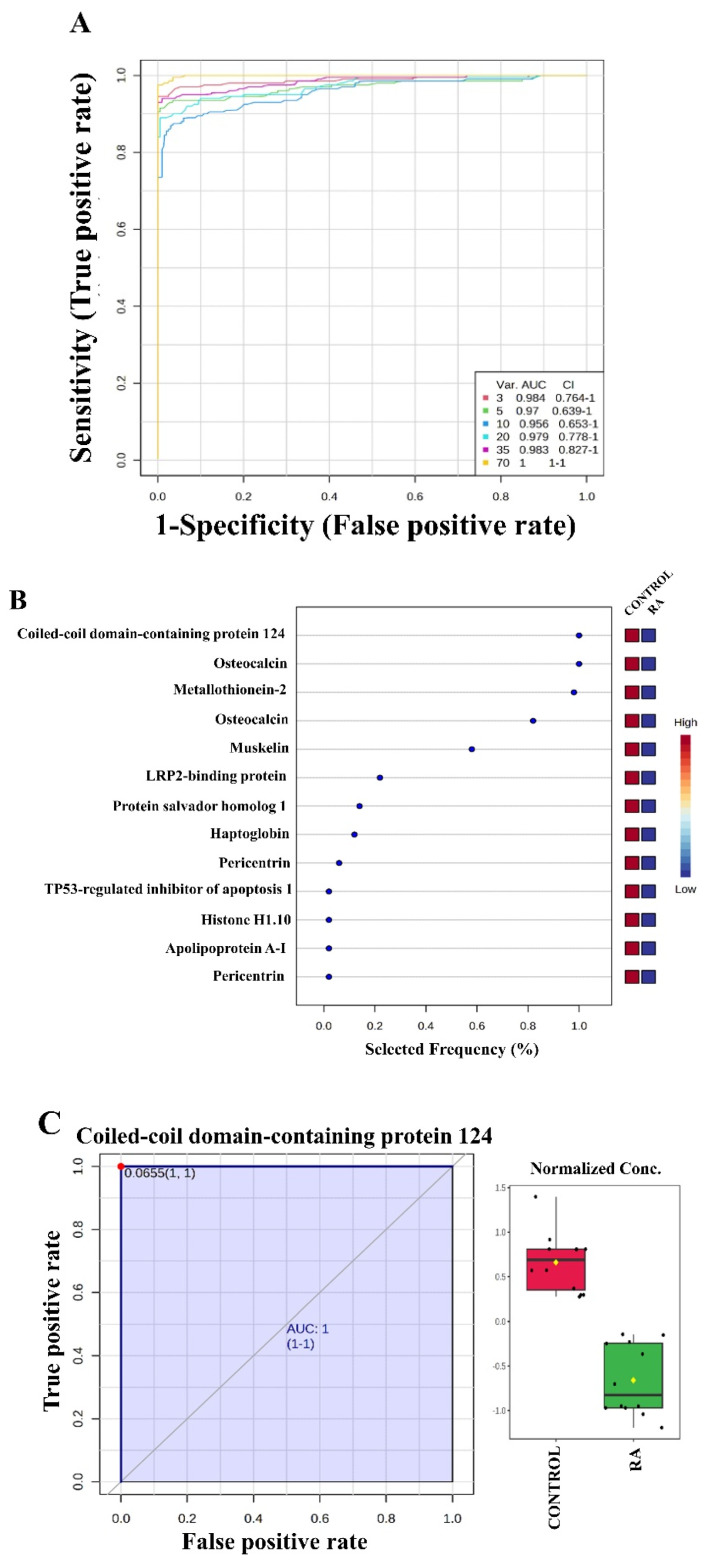

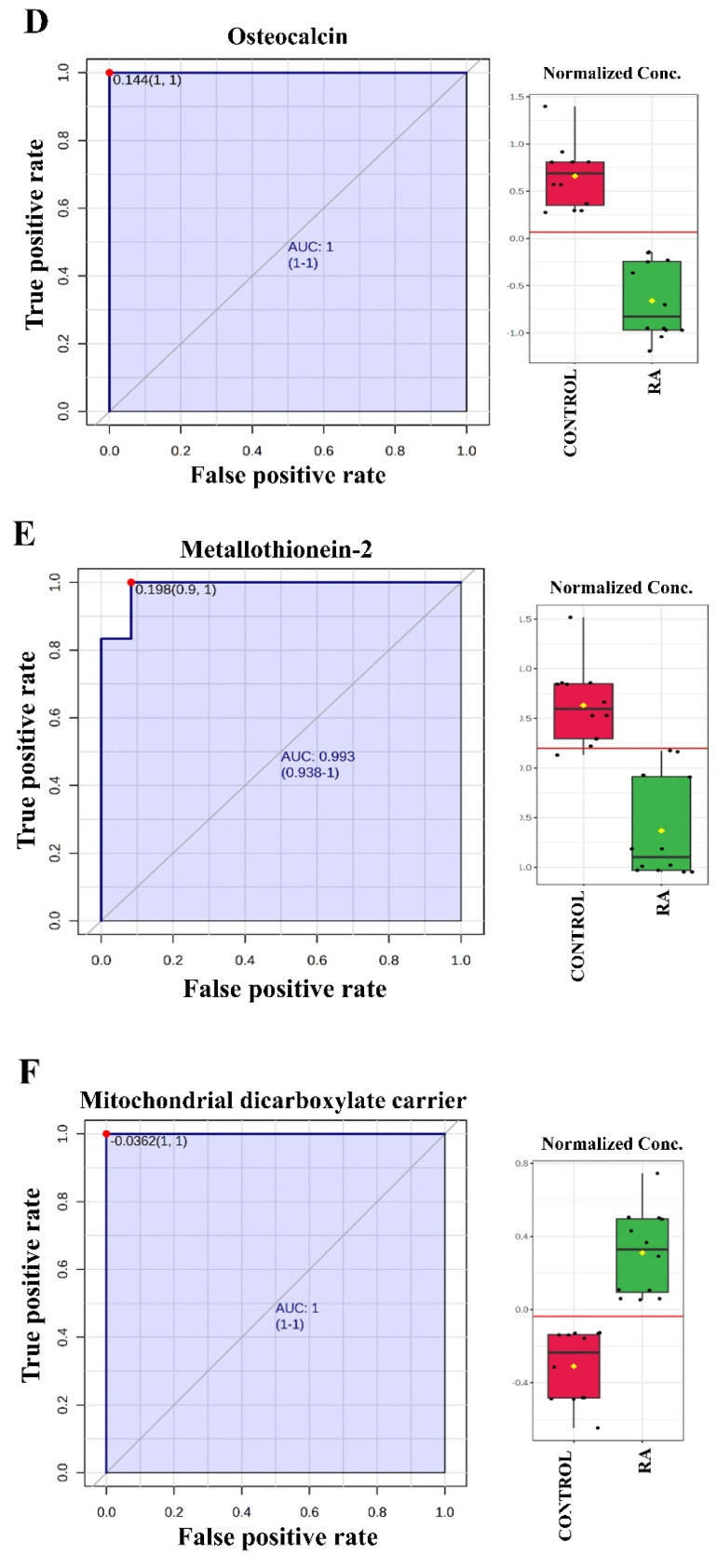
Multivariate biomarker analysis using the receiver operating characteristic (ROC) curve in patients with rheumatoid arthritis (RA) and control groups, using PLSDA as the feature ranking method. Multiple ROCs were generated using the biomarker analysis module of the MetaboAnalyst software (Montreal, QC, Canada) (www.metaboanalyst.ca). (**A**) The values of area under the curve (AUC between different models using 3, 5, 10, 20, 35, and 70 of the identified proteins). (**B**) The top 10 proteins with the highest selected frequency. The individual ROC for (**C**–**E**) down- and (**F**) upregulated proteins in RA along with the Box plot (FDR *p* ≤ 0.05 and fold change ≥ 1.5), where red represents the control, and green represents RA.

We further analyzed the proteins using multivariate exploratory ROC analysis based on the identified common and significantly dysregulated proteins between the RA and control (*n* = 69) groups. The ROC curves were generated using PLS-DA as a classification and feature ranking method. The highest AUC of the exploratory ROC curve for a model with three proteins was 0.984 (Figure 5A). The frequency plot of the top 13 significantly dysregulated identified protein biomarkers in the RA and control groups showed that coiled-coil domain-containing protein 124, osteocalcin (OC), metallothionein-2, muskelin, etc., were downregulated in the RA group (Figure 5B). Four proteins showing the highest AUCs are shown, namely coiled-coil domain-containing protein 124 (AUC of 1, Figure 5C), OC (AUC of 1, Figure 5D), metallothionein-2 (AUC of 0.933, Figure 5E), and mitochondrial dicarboxylate carrier (AUC of 1, Figure 5F).

**Figure 6:**
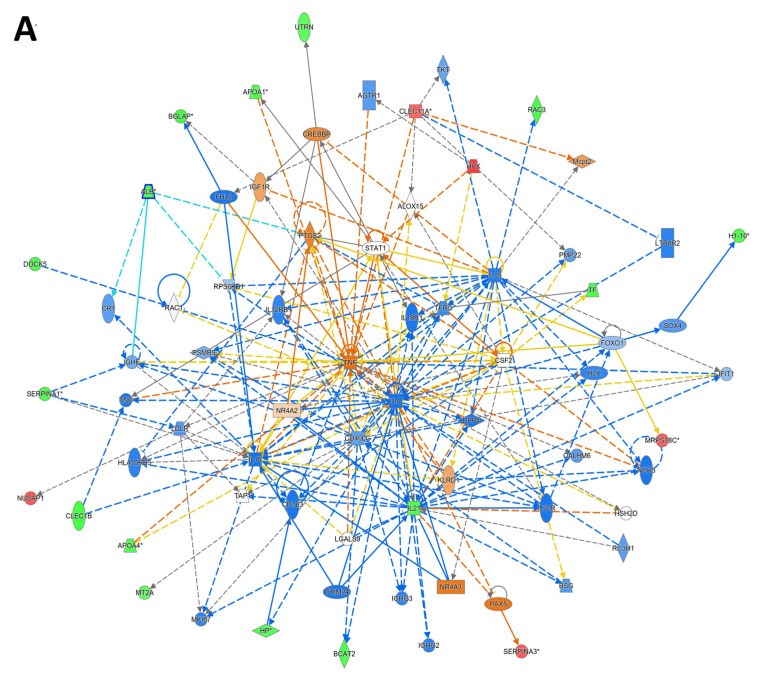

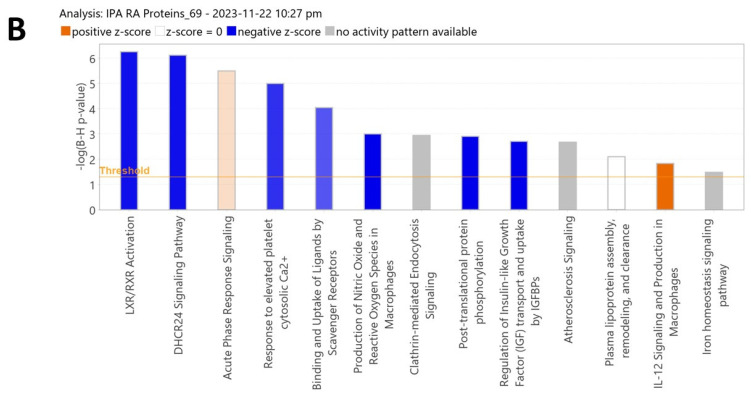
Network and biological pathway analysis relating to the significantly identified proteins in the study. (**A**) Network pathway analysis of the significantly dysregulated proteins identified in the RA group compared to the control group revealed dysregulation in pathways related to cell-to-cell signaling and interaction, hematological system development and function, and immune cell trafficking. The pathway identified TNF and IFNG as the central nodes. (**B**) The top canonical pathways related to the DAPs were LXR/RXR activation, DHCR24 signaling pathway, acute-phase response signaling, and response to elevated platelet cytosolic Ca^2+^ and IL-12 signaling. The blue color indicates an inhibition, while the orange color indicates an activation of these pathways between the RA and control groups.

The significant DAPs identified between the RA and control groups in our study were uploaded into the IPA software. IPA maps the proteins in our dataset to known proteins and their biological pathways. The DAPs were mapped to three major network pathways. The network with the highest score (score = 37) showed that these proteins were focused around the dysregulation of STAT1, TNF, and IL4 signaling pathways and were related to cell-to-cell signaling and interaction, hematological system development and function, and immune cell trafficking with a score of 37. The top canonical pathways identified were related to LXR/RXR activation (*p*-value of 2.76 × 10^−9^ and overlap of 5.7%, 7/122), the DHCR24 signaling pathway (*p*-value of 7.60 × 10^−9^ and overlap of 5.0%, 7/141), acute-phase response signaling (*p*-value of 4.79 × 10^−8^ and overlap of 3.8%, 7/184), response to elevated platelet cytosolic Ca^2+^ (*p*-value of 2.00 × 10^−7^ and overlap of 4.4%, 6/136), and binding and uptake of ligands by scavenger receptors (*p*-value of 2.25 × 10^−6^ and overlap of 4.4%, 5/113) (Figure 6A,B).

### 2.7. Multiple Reaction Monitoring (MRM) Mass Spectrometry

**Figure 7:**
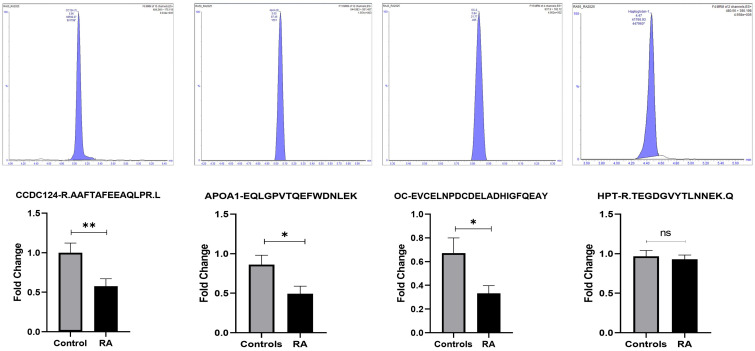
Multiple reaction monitoring (MRM) mass spectrometry for validating study findings. The MRM method based on signature peptides was developed to validate the expression of four proteins, namely CCDC124, Apo-A1, OC, and HPT, found in the proteomics approach (2D-DIGE MALDI-TOF-MS). The top panel shows representative MRM chromatograms of selected peptides asterisk (*) indicates the extrapolated concentration value. The bottom panel shows expression of these four proteins preesnted as fold changes between the RA and control groups. Statistical significance was evaluated using an unpaired *t*-test (*n* = 120), in which * represents *p* ≤ 0.05, ** represents *p* ≤ 0.01 and ns represent non-significant.

Four significantly dysregulated proteins from the 2D-DIGE proteomic profile were selected for validation. Signature peptides for the selected proteins were identified using criteria described previously [17]. Proteins were selected based on the highest selected frequency and on their involvement in the protein–protein interaction network pathway. Proteins with a higher number of interactions and fold changes showing significant changes in abundance (CC124, APOA1, OC, and HPT) were used to confirm the findings. The uniqueness and reliability of these signature peptides were confirmed using Skyline Software V3 and PeptideAtlas (Human 2025-01, https://peptideatlas.org/, access date 14 March 2025) [18]. The MRM method was optimized using triple-quadrupole mass spectrometry (LC-MS/MS). Representative chromatograms for each protein signature peptide are shown in Figure 7 and Table S4. This validation experiment shows that these four selected proteins have similar expression trends compared to the MALDI-TOF results, as shown in Figure 7, with a different fold-change value. The expression profiles of these proteins were statistically evaluated using unpaired *t*-tests with PrismPad Software version 8 (Dotmatics, Boston, MA, USA).

## 3. Discussion

The present study identified the changes in the proteome between established cases of RA and the controls. The pathophysiology of RA is multifactorial and involves multiple different immunological and inflammatory pathways. Routine laboratory measurements of acute-phase reactants, such as erythrocyte sedimentation rate (ESR) and levels of C-reactive protein (CRP), are commonly used to assess disease activity when following patients with RA. The levels of both these markers were significantly elevated in the RA group. Our plasma proteomic analysis revealed significant dysregulation of numerous proteins involved in extracellular matrix remodeling, bone metabolism, immune response, cell signaling, glycosaminoglycan synthesis, and acute-phase response proteins in patients with RA compared to the controls. The majority of the DAPs had a decreased abundance when compared to the controls.

### 3.1. Significantly Increased Proteins in RA Compared to Controls

Among the DAPs that showed an increased abundance was mitochondrial dicarboxylate (DIC) carrier protein. Mitochondria are major energy-producing organelles that have central roles in cellular metabolism. Mitochondrial activity affects the differentiation, activation, and survival of immune and non-immune cells that contribute to the pathogenesis of this disease [19,20]. DIC belongs to the solute carrier family 25 (SLC25) transport proteins involved in the transport of dicarboxylates, participating in the TCA cycle within the immune and non-immune cells, regulating cellular energy production and influencing immune responses in the context of RA [21]. It is also known to regulate mitochondrial glutathione transport intracellular ROS levels. The knockdown of SLC25A10 induces sensitivity to glutamine deprivation, decreases NADP and NAPDH levels, and is associated with a low expression of key genes involved in the antioxidant system, including thioredoxin 2 (TXN2) and thioredoxin reductase 2 (TXNRD2), thereby increasing sensitivity to oxidative stress [22]. Although the precise mechanisms remain an area of ongoing research, the involvement of DIC in cellular metabolism may have relevance to the altered proteomic profile observed in RA. Targeting mitochondrial metabolism has emerged as a promising avenue for developing potential treatments in RA, as it may offer new opportunities to modulate immune responses and attenuate inflammatory processes. Dysfunctional mitochondrial metabolism has been demonstrated to lead to increased oxidative stress and inflammation, contributing to the perpetuation of RA pathology. Understanding the specific role of the mitochondrial dicarboxylate carrier in RA could unveil novel targets for therapeutic intervention.

Another notable protein with increased abundance in our study was hemopexin. Hemopexin is an acute-phase protein that is involved in modulating the inflammatory and immune response and also acts as an antioxidant by scavenging free heme. In animal models, hemopexin was demonstrated to downregulate the secretion of TNF and IL-6 from murine bone macrophages, following stimulation with TLR4-dependent agonists, and inhibit cytokine secretion, thereby decreasing inflammation. In another study, a deficiency in HPT and hemopexin was shown to have a negative regulatory role in Th17-mediated inflammation by favoring the differentiation of naïve CD4+ T cells towards the Th17 lineage and enhancing the stabilization and expansion of memory Th17 cells by IL-23 [23,24,25]. We identified multiple spots related to hemopexin in our study that can represent different proteoforms. Multiple proteoforms of hemopexin, primarily resulting from oxidation, differential glycosylation, and limited proteolysis, have been identified in different conditions, including fibromyalgia [26,27]. These may differ in their heme-binding efficiency and immunomodulatory properties. A recent study identified carbamylated hemopexin as a biomarker in patients with early RA [28]. An increase in hemopexin levels in our group of patients may indicate a decrease in differentiation of T cells and a decrease in inflammation [23,29].

### 3.2. Significantly Decreased Proteins in RA Compared to Controls

A significant decrease was noted in the protein OC in patients with RA compared to the controls. OC, also known as bone GLA, is a bone matrix component representing non-collagenous proteins released into the circulation during bone formation. Although generally considered a surrogate marker for evaluating ongoing bone remodeling and bone formation and resorption [30,31], it is now known to have a much broader role that involves the regulation of whole-body metabolism, insulin regulation, and even cognition [32]. RA is a commonly considered a form of inflammatory polyarthritis, which, in established cases, is associated with a greater risk of bone loss and osteoporosis [33]. The deterioration of the bone is considered to be due to the actions of polymorphonuclear leukocytes that act in concert with osteoclasts under the influence of cytokines, including TNF and IL1, as well as other cytokines, including IL-17 [34]. While T and B cells represent the immunological aspects of RA, most of the damage from the disease is driven through effector cells and their products, including cytokines and other mediators. The fate of immune cells, besides immune metabolism, is regulated by diverse metabolic processes. Alterations in cellular metabolic states can influence immune cell behavior, contributing to susceptibility to diseases such as type 2 diabetes mellitus, cardiovascular disease, obesity, and cancer. Osteoporosis represents a well-known extra-articular manifestation of RA, and treatment with disease-modifying antirheumatic drugs is known to decrease erosive bone loss. Clinical studies addressing bone loss in RA have yielded conflicting results. Seriolo et al. found that serum osteocalcin concentrations in patients with active RA were found to be significantly lower than those in patients with remission of RA and in healthy controls [35], while other authors found the levels to be similar to those in controls [36] or increased [37]. Decreased levels of circulating OC may not only indicate a decrease in bone remodeling but also an overall decrease in body metabolism. A recent study showed that B cells from RA patients express molecules that inhibit osteoblast differentiation and inhibit bone formation. A significant decrease in the levels of OC in these patients may indicate a much broader role of osteoimmunology in RA. OC undergoes post-translational gamma-carboxylation at three residues, 17, 21, and 24. Based on the degree of carboxylation, it is known to exist in the carboxylated, undercarboxylated, and uncarboxylated forms, with each having distinct functions. In this regard, we found multiple spots of OC with a significant decrease in abundance on the 2D-DIGE gels. Uncarboxylated OC, unlike the carboxylated fraction, is known to have an important role in the modulation of glucose, insulin, and energy metabolism [38,39] and could have a potential impact on metabolic homeostasis in patients with RA. The decrease in OC levels was validated through MRM analysis and was statistically significant.

The levels of HPT, an acute phase reactant protein, were observed to be decreased in patients with RA compared to controls. Additionally, HPT acts as an antioxidant by scavenging free hemoglobin, thereby protecting tissues from oxidative stress and through its binding to neutrophils. It also modulates the inflammatory and immune responses by controlling cytokine production and binding to different immunologic cells, including CD8 T cells and human B cells that are known to be dysregulated in autoimmunity, including RA. Characteristically, HPT is known to have various proteoforms derived from genetic polymorphisms (HP1-1, Hp2-2 proteoforms) and post-translational modifications, especially glycosylation. Among its proteoforms, the Hp 2-2 genotype has been previously identified as an independent risk factor for CVD in patients with RA [40]. Different glycosylated HPT proteoforms, characterized by increased fucosylation and decreased mannosylation, have also been previously identified in the serum of patients with alcoholic cirrhosis, cancer, and RA [41]. In our study, we identified multiple spots with a decreased abundance related to HPT, which could indicate the presence of these different proteoforms in RA. MRM validation for HP was carried out between the cases and controls but was not statistically significant and warrants further studies.

A significant decrease in abundance was noted in a novel coiled-coil domain-containing protein-124 (CCDC124). CCDC124 is a putative mRNA-binding protein containing a coiled-coil domain motif involved in protein–protein interaction. These proteins are expressed in a wide range of tissues, with diverse localization, thereby exhibiting functional roles in most of the physiological processes [5]. Previous studies have shown its important role in cellular physiology and RNA metabolism, in regulating cytokinesis, and in the process of translation during mitotic cell division [20]. Upregulation of the CCDC124 gene was noted in cases of cancer, and its mRNA levels are considered molecular prognostic signatures in breast, ovarian, endometrial, and urinary bladder cancers [42]. Although CCDC124 has primarily been studied in the context of cancer, its roles in regulating cytokinesis and translation during mitosis may be critically relevant in autoimmune diseases such as RA, where aberrant immune function and cell division are observed. The decrease in levels of CCDC124 in patients with RA was confirmed through MRM in our validation cohort.

The levels of two apolipoproteins (APOA1 and APOA IV) were observed to have a decreased abundance in patients with RA compared to controls. Our findings are in line with other studies that have also shown a decrease in circulating levels of Apoa1 [43,44]. Apolipoproteins are well-known for their roles in the regulation of lipid metabolism. Besides this role, they have been recently implicated in the regulation of immune cells and as negative acute-phase proteins that inhibit TNF-α and IL-1β production induced by stimulated T cells in monocytes or monocytic cells [45,46,47,48]. ApoA1 also regulates macrophage activation sites by T lymphocytes through its binding to surface factors on stimulated T cells. It acts by limiting contact-mediated cytokine induction in monocyte–macrophages and inhibits critical pathways associated with disease exacerbation [49]. MRM validation confirmed the decrease in levels of ApoA-1. These findings indicate that ApoA-I has anti-inflammatory properties and may have important implications for the treatment of chronic inflammatory diseases. In addition to ApoA1, a decrease in abundance of Apo AIV was also noted in RA compared to the controls. ApoA-IV is a multifunctional protein with anti-inflammatory properties. In mice models, Apo AIV was shown to reduce the secretion of pro-inflammatory cytokines and regulate immune cell infiltration [50]. Aside from inflammation, it is involved in other physiological processes, including lipid metabolism, as an antioxidant, and in coagulation and glucose homeostasis, while its deficiency has been associated with atherosclerosis and diabetes. A decrease in the levels of this protein in RA may predispose these patients to continued inflammation, along with the dysregulation of lipid and glucose metabolism and increased levels of resting mast cells, CD8 T cells, and follicular helper T cells [51], a characteristic feature of RA.

Similarly, there was a decrease in the abundance of the protein spots related to the enzyme heparan-sulfate 6-O-sulfotransferase 2 in the RA group compared to the control group. HS6ST2 is a member of a family of enzymes involved in the sulfation of heparan sulfate, which is critical for its synthesis and functioning [52]. Heparin/heparan sulfate (HS) is a member of the glycosaminoglycan family of polysaccharides involved in leukocyte transmigration, an essential feature of the inflammatory response, in the recruitment of leukocytes from the blood, and in the activation of chemokines [53]. 6-O-sulfation alters the affinity of HS to bind ligands, including signaling factors and molecules. Increased levels promote cartilage damage and were seen to be a feature in osteoarthritis [54]. It is to be noted that patients with RA show an alteration in the transmembrane glycoproteins or lipids expressed on the surface of different cells. Many of these cell surface proteins or lipids are responsible for propagating the inflammatory immune response, including the cluster of differentiation (CD) molecules, such as CD24, CD28, and CD40, that act as potent T-cell co-stimulatory factors. CD40 leads to the occurrence and progression of RA [2,55]. Measuring the changes in the levels of this enzyme could be an indicator for the glycosylation of glycosaminoglycans and also a potential marker for RA.

It is known that both cell proliferation and apoptosis were dysregulated in RA, and lymphocyte auto-reactivity and cell cycle interruptions are causes of autoimmunity. In RA, the dysregulation of cell proliferation and apoptosis have been reported [56]. This could be contributed to by the decrease in proteins muskelin and TP53-regulated inhibitor of apoptosis 1, which are involved in regulating the proteasomal degradation and apoptotic pathways, as seen in proteomic analysis. Muskelin is a component of the CTLH E3 ubiquitin-protein ligase complex that selectively mediates ubiquitination and subsequent proteasomal degradation [57]. It plays a role in regulating pathways controlling homeostasis and development and in the migration of cells, including lymphocytes [58]. TP53-regulated inhibitor of apoptosis 1 is involved in the modulation of the mitochondrial apoptotic pathway. Impaired apoptosis and proliferation resulted in autoreactive lymphocyte development and inflammation in RA. Our findings are in line with Ebrahimian et al., who showed that downregulated expression in RA PBMCs could be correlated with RA pathogenesis by regulating apoptosis, cell survival, inflammatory mediator production, and proliferation [56]. Bioinformatic analysis using IPA highlighted the role of STAT1, TNF, and IL4 as the central nodes dysregulated in RA. These signaling nodes are key mediators of pro- and anti-inflammatory pathways that govern immune cell activation, differentiation, and trafficking. STAT1 is known to be involved in macrophage activation and interferon signaling, often contributing to sustained inflammatory responses in RA, and in some cases, is used to predict response to treatment [59]. On the other hand, TNF is a well-established pro-inflammatory cytokine that drives joint inflammation, cartilage degradation, and systemic immune activation. IL-4, while classically anti-inflammatory, also modulates immune responses, especially Th2 responses, and may influence the balance between regulatory and effector T cells in the RA microenvironment. The identification of cell-to-cell signaling, hematopoietic system development, and immune cell trafficking network as the highest scoring pathway associated with the DAPs highlights its relevance to the chronic inflammatory milieu that characterizes RA. Future studies need to be carried out to confirm our findings within a larger cohort and by using other techniques such as LC-MS/MS.

## 4. Materials and Methods

### 4.1. Ethical Approval, Patient Recruitment, and Consent to Participate

The study procedures and protocols were reviewed and obtained from the institutional review board of the College of Medicine, King Saud University (IRB number: E-21-6341). The primary physician confirmed the RA diagnosis based on ACR/EULAR 2010 criteria [60,61]. The patients in the present study were recruited from those attending the rheumatology outpatient clinics at King Khalid University Hospital, College of Medicine, King Saud University, in the age group of 36–65 years. A total of 12 patients with known RA for a mean duration of 20 years in clinical remission (DAS28 < 2.6), mostly women, were selected from a cohort for the discovery phase, and 60 patients were selected for the validation phase. Control plasma samples were obtained from the blood bank and age-matched volunteers who were otherwise healthy and used in the discovery and validation phases. Their attending physician carried out the clinical assessment, and those willing to participate consented. The sample size was determined by conducting a power analysis using the Progenesis SameSpots non-linear dynamics statistical software to determine the minimum number of biological replicates required. The baseline demographic information and clinical laboratory parameters, including complete blood count with an erythrocyte sedimentation rate (ESR), lipid markers, liver function tests, fasting glucose, glycated hemoglobin, and C-reactive protein (CRP), were collected from the patient’s medical records (Table 1). The study procedures were conducted according to the ethical standards of the Declaration of Helsinki and the International Conference on Harmonization Good Clinical Practice guidelines. Blood samples were drawn in EDTA-containing vacutainers, and plasma was separated based on the standard protocol. The plasma samples were aliquoted and stored at −80 °C until further processing.

### 4.2. Sample Processing by Depletion Followed by Precipitation of Proteins

All plasma samples were processed with Top-20 Depletion ProteoPrep spin columns (Sigma-Aldrich, Saint Louis, MO, USA) to deplete the abundant proteins to decrease the sample complexity. Due to their abundance, these plasma proteins, including immunoglobulins, albumin, alpha-1 antitrypsin, and transferrin, mask the underlying low DAPs and are depleted before precipitation. Protein precipitation was carried out using the TCA/acetone (10% *w*/*v*) method, added to the depleted protein in a ratio of 1 to 4, vortexed for 15 s, and left overnight at −20 °C. The following day, sample mixtures were centrifuged (2000× *g* for 15 min at 4 °C), and the protein pellets obtained were re-suspended in labeling buffer (7 M urea, 2 M thiourea, 4% CHAPS, 30 mM Tris). All the samples were checked for their pH, and it was adjusted to 8.5 where required. Following this, the protein concentration of all the samples was estimated by the 2D-Quantkit (GE Healthcare, Danderyd, Sweden) in duplicate.

### 4.3. D-DIGE Gel Electrophoresis for Protein Separation and Image Scanning

Cye dye labeling was performed for the samples using CyDye™ DIGE Fluor minimal dye. Briefly, 400 pmol (GE Healthcare) of the cye dye, freshly dissolved in anhydrous dimethyl formamide (DMF), was used for the fluorescent labeling of 50 μg of protein taken from each sample using the dye switching technique between the control and RA patient samples using either Cy3 or Cy5 [62,63,64,65]. A pooled internal standard of 50 μg of total protein from each of the 24 samples was labeled with Cy2. After the addition of the cye dye, the samples were incubated in the dark on ice for the reaction, covalently linking with a fluorophore. Subsequently, the reaction was quenched by adding 1.0 μL of lysine (10 mM) and ice for 10 min in the dark. The samples were combined according to the experimental design (Table S1) and run on the same gel for comparison. Twelve Immobiline Dry Strips (24 cm, pH 3–11; GE Healthcare) were rehydrated passively (30 V, 12 h) and were used to carry out the first-dimensional analytical gel electrophoresis. The isoelectric focusing using an Ettan IPGphor IEF unit (GE Healthcare) was performed at 20 °C and 50 μA per strip, according to the following steps and hold sequence: (1) 500 V for 1 h, (2) 1000 V for 1 h, (3) 8000 V for 3 h, and (4) 8000 V for 45,000 Vh. The IPG strips were next equilibrated in the equilibration solution (5 mM Tris–HCl, pH 8.8, 6 M urea, 30% glycerol, 2% SDS) at room temperature for 15 min each, with a solution containing 65 mM DTT, followed by a solution containing 250 mM iodoacetamide. The second, using sodium dodecylsulfate-polyacrylamide gel electrophoresis (SDS-PAGE, 5–20%) prepared using low-fluorescent glass, was performed (six vertical units, Ettan DALT, GE Healthcare, Danderyd, Sweden; 15 °C, 1 W per gel for 1 h, and then 2 W per gel until the bromophenol blue dye front reached the bottom of the gel). After SDS PAGE electrophoresis, the gels were scanned with the Sapphire Biomolecular Imager (Azure Biosystems, Dublin, OH, USA) and digitalized via the image analysis software Sapphire Capture system (version 1.12.0921.0) (Azure Biosystems, Dublin, OH, USA). Preparative gels were prepared using total protein (1 mg) obtained from a pool of equal protein amounts from the 24 depleted plasma samples. Gels were stained for 5 days, and the stained gels were briefly rinsed with Milli-Q water and stored until the spots could be picked and identified using MS, as previously described [62,63,64,65].

### 4.4. Statistical Analysis for Determining Differentially Abundant Proteins (DAPs) Spots

2D-DIGE gel images were analyzed for differences in spot intensities using an automated spot detection method, Progenesis SameSpots software v.3.3 (Nonlinear Dynamics, Newcastle upon Tyne, UK), which included modules for gel warping, DIGE normalization, and comparison. The gel images from individual analytical gels were imported into the software, aligned to the reference gel, and overlaid to ensure that no data were lost. The normalized volume (NV) of each gel from the ratio of Cy3 (or Cy5) to Cy2 spot volume was calculated, and the spot volumes were log-transformed to generate normally distributed data. Log-normalized volume (LNV) was used to quantify the differential expression by directly comparing patient and control groups. All spots were pre-filtered and manually checked before applying statistical criteria using two cutoffs (analysis of variance (ANOVA), FDR *p* ≤ 0.05, and fold change (FC) > 1.5). Instead of spot intensities, normalized spot volumes were used for statistical processing. An additional univariate analysis using a volcano plot analysis was performed for each binary comparison to identify significantly differentially expressed proteins based on a fold-change criterion > 1.5, with a false discovery rate (FDR)-adjusted *p*-value less than 0.05. The *x*-axis on the volcano plot represents the fold change (FC) between the two comparison groups, while the y-axis represents the *p*-value.

### 4.5. MALDI-TOF-Based Mass Spectrometric Analysis for Protein Identification

Coomassie-stained gel spots were excised manually, washed, and digested according to previously described methods [62,63,64,65]. A mixture of tryptic peptides (0.8 μL) derived from each protein was spotted onto a MALDI target (384 MTP Anchorchip; 800 μm of Anchorchip; Bruker Daltonics, Bremen, Germany). MALDI-MS/MS spectra were obtained using an UltraflexTerm TOF mass spectrometer equipped with a LIFT-MS/MS device (Bruker Daltonics) at reflector and detector voltages of 21 kV and 17 kV, respectively, as described before [62,63,64,65]. Peptide mass fingerprints (PMFs) were calibrated against a standard (peptide calibration standard II, Bruker Daltonics). The PMFs were assessed using the Flex Analysis software (version 2.4, Bruker Daltonics). MS data were interpreted using BioTools v3.2 (Bruker Daltonics). The peptide masses were searched using the Mascot search algorithm (v2.0.04, updated on 9 May 2021; Matrix Science Ltd., London, UK). The Mascot significance score was calculated using the formula Protein score = −10 × Log(P), where P is the probability that the observed match is a random event; a Mascot score greater than 56 was considered significant (*p* ≤ 0.05) and was accepted. ID proteins with low scores were excluded, as they were mostly random matches and insignificant (*p* > 0.05). The Mascot parameters were as follows: fixed cysteine modification with propionamide, variable modification due to methionine oxidation, one missed cleavage site in case of incomplete trypsin hydrolysis, and a mass tolerance of 100 ppm. Identified proteins were accepted as correct if they showed a Mascot score greater than 65. Not all spots of interest could be identified because some proteins were low-abundance and did not yield sufficiently intense mass fingerprints; other spots were mixtures of multiple proteins [62,63,64,65].

### 4.6. Biomarker and Bioinformatics Analysis

Biomarker analysis of the proteomics expression profiles was performed using MetaboAnalyst version 6.0 (McGill University). The raw data were normalized to the sample’s total median to ensure that all samples were distributed normally, and they were corrected to make individual features more comparable by using log-transformation and Pareto-scaling, respectively. Because the data were Gaussian-distributed, the unpaired two-tailed Student’s *t*-test was used for binary comparisons between any two study groups, where the significance levels for the protein data were considered at a false discovery rate (FDR)-corrected *p* < 0.05 with a 1.5-fold cutoff change; the values are presented as the mean ± SEM. Potential biomarkers were assessed using receiver operating characteristic (ROC) curve analysis performed via MetaboAnalyst (version 6.0, McGill University). ROC curves were generated based on an orthogonal partial least squares-discriminant analysis (OPLS-DA) model. Model robustness was evaluated through the fitness-of-model (R^2^Y) and predictive ability (Q^2^) metrics, where R^2^Y values approaching 1 and Q^2^ values above 0.5 indicate strong model performance [65]. The DAPs were classified into different categories according to their molecular function and biological process using the PANTHER (Protein Analysis Through Evolutionary Relationships) classification system (http://www.pantherdb.org (accessed on 23 July 2024)). The interaction between the statistically significant differentially abundant proteins—the protein–protein interaction (PPI) network—was also constructed using Ingenuity Pathway Analysis (IPA) version 9.0 (Ingenuity Systems, Redwood, CA, USA) [62,63,64,65]. The IPA software maps the UniProt IDs into the ingenuity knowledge base, a manually curated resource containing data from all published scientific studies and considered the world’s largest. The list of identified proteins, with the FC and FDR *p*-values, was entered into the online software to define the interactions, key biological pathways, and molecular roles of the identified proteins between RA and control groups. The software-generated network maps provide a means of visualizing both direct and indirect protein–protein interactions and relate them to known biological and canonical pathways [61,62,63,64,65].

### 4.7. Validation of Results Using LC- MS/MS (MRM)

Four different proteins were selected from the proteomics profile, where at least one signature peptide per protein was identified using the criteria described previously, using Skyline Software v21 (MacCross Lab, Seattle, WA, USA) [17,18]. The suggested MRM transitions were exported to a Triple-Quadrupole-Tandem Mass spectrometer (XEVO TQmicro, Waters Corporation, Milford, MA, USA). A total of 120 samples were used for validation. A control-extracted sample (n = 60) was used to evaluate the calculated transitions and to optimize the collision energy and column retention time. The patient samples (n = 60) were digested with trypsin and solid-phase-extracted using the standard protocol reported by Galal et al., 2021 [66]. The validation batch of these samples was started with a calibration curve (7 points) and the 3 levels of QCs (low, medium, and high); all of these QCs and the calibration curves were evaluated with accuracy 85–115% and CV% < 15, with overall success > 67% of all QCs based on CLIA and FDA Guidelines. A pool of QC samples was also added as an extra control level to ensure a whole process of sample analysis control with <20 CV%. The same is now included in the manuscript. The extracted tryptic peptides were separated using an Acquity Ultra-Performance Liquid Chromatography (UPLC) (AQUITY BEH C18, 1.7 μm, 2.1 mm × 100 mm) column (at 25 °C) at a mobile phase flow rate of 0.3 mL/min over a total run time of 12 min (solvent A: 0.1% formic acid in H2O; solvent B: 0.1% formic acid in acetonitrile). The gradient profile for solvent A (0.1% formic acid in H2O) was 90% for 1 min, followed by a linear gradient to 10% over 10 min, which was then held at 10% for 1 min before returning to 90% in 2 min. For positive-mode mass spectrometric resolution, the eluted peptides were subjected to electrospray ionization (ESI). The source desolvation temperature was set to 450 °C, the desolvation gas flow was set to 700 L/h, the cone gas flow was set to 50 L/h, the MS capillary source voltage was set to 1.98 KV, and the cone source was set to 47 V. The total run time for each sample was 12 min at a mobile phase flow rate of 0.3 mL/min following the gradient table. The samples were stored in an autosampler at 4 °C, with an injection volume of 5 μL. During the run, frequent intermediate washing steps were performed to minimize the sample carryover. The experimental conditions are summarized in Supplementary Table S4.

## 5. Conclusions

A comprehensive plasma proteomic profiling of RA unveiled significant differences in proteins, underscoring the multifaceted nature of RA. The identified proteins were associated with inflammation, immune regulation, metabolism, and cellular homeostasis. The identification of differentially abundant proteins—such as the upregulation of mitochondrial dicarboxylate carrier and hemopexin and the downregulation of osteocalcin, apolipoproteins (ApoA1 and ApoA-IV), and CCDC124—reflects the complex interplay between metabolic dysfunction, immune cell dysregulation, impaired bone remodeling, and defective apoptotic pathways in RA pathophysiology. These proteins not only contribute to underlying disease pathology but also hold promise as potential biomarkers or therapeutic targets.

## Figures and Tables

**Table 1:** Baseline demographic and clinical characteristics of patients with rheumatoid arthritis and control groups in the discovery and validation phases.

Characteristics	Cases (Mean ± SD) (*n* = 12)	Controls (Mean ± SD) (*n* = 12)	*p*-Value	Cases (Mean ± SD) (*n* = 60)	Controls (Mean ± SD) (*n* = 63)	*p*-Value
	Discovery Phase			Validation Phase		
Duration (yrs)	14 ± 9			16 ± 7		
Age	46.8 ± 12.4	41.5 ± 6.2	0.19	49.8 ± 12.045	47.5 ± 13.86	0.32
BMI (Kg/m^2^)	24.2 ± 3.8	22.8 ± 4.2	0.40	29.1034 ± 7.2	29.9 ± 6.21	0.52
HB (gm/L)	126.8 ± 8.7	132.3 ± 14	0.26	127.2 ± 9.3	129.7 ± 9.6	0.44
WBC 10^9^/L	6.5 ± 1.8	6.9 ± 2.7	0.60	6.8 ± 2.2	6.6 ± 2.4	0.59
RBC (10^12^/L)	4.4 ± 0.3	4.6 ± 0.6	0.15	4.8 ± 0.6	5.1 ± 1.3	0.07
Lymphocyte (10^9^/L)	2.4 ± 0.8	2.5 ± 0.7	0.61	2.2 ± 0.7	2.4 ± 0.5	0.55
Neutrophil (10^9^/L)	3.3 ± 1.3	3.6 ± 2.1	0.62	3.2 ± 1.3	3.1 ± 1.5	0.09
ESR (mm/hr)	48.5 ± 26.8	5.8 ± 2.1	<0.001 *	43.0 ± 23.8	5.6± 2.8	<0.001 *
ALT (IU/L)	20.5 ± 4.2	19.7 ± 9.3	0.7	18.99 ± 7.06	18.04 ± 9.01	0.08
AST (IU/L)	17.2 ± 6.7	17.4 ± 4.1	0.9	19.0 ± 7.0	16.7 ± 7.3	0.08
Albumin (gm/L)	38.4 ± 7.6	40.2 ± 3.3	0.3	39.9 ± 6.5	41.3 ± 4.9	0.22
Glucose(mmol/L)	4.9 ± 0.5	4.6 ± 0.4	0.6	5.48 ± 1.12	5.15 ± 0.85	0.07
Chol (mmol/L)	4.4 ± 1.2	4.3 ± 0.7	0.7	4.63 ± 0.78	4.77 ± 0.94	0.36
HDL (mmol/L)	1.4 ± 0.3	1.4 ± 0.4	0.5	1.47 ± 0.410	1.49 ± 0.37	0.316
LDL (mmol/L)	2.8 ± 1.0	2.4 ± 0.5	0.2	2.99 ± 0.81	2.70 ± 0.80	0.18
Triglycerides (mmol/L)	1.2 ± 1.2	1.1 ± 0.3	0.7	1.19 ± 0.74	1.27 ± 0.54	0.507
HbA1c	5.4 ± 0.5	5.2 ± 0.4	0.1	5.74 ± 0.54	5.71 ± 0.84	0.916
CRP (mg/L)	10.0 ± 7.8	2.4 ± 1.7	<0.001 *	9.70 ± 7.40	1.9 ± 3.4	<0.001 *

Values are expressed as mean ± standard deviation (SD) in the parenthesis. A significant difference between RA and control (independent *t*-test, *p*-value < 0.05 for parametric tests). BMI: body mass index, HB: hemoglobin, WBC: white blood cells, RBC: red blood cells, PLT: platelets, ESR: erythrocyte sedimentation rate, ALT: alanine aminotransferase, AST: aspartate transaminase, HDL: high-density lipoprotein, LDL: low-density lipoprotein, HbA1c: glycated hemoglobin, CRP: C-reactive protein.* *p*-Value < 0.05 is considered as statistically significant.

## Data Availability

The datasets presented in this study can be found in online repositories. The names of the repository/repositories and accession number(s) can be found below: ftp://massive-ftp.ucsd.edu/v09/MSV000097902/ (accessed on 15 May 2025).

## Supplementary Materials

The Supplementary Material for this article can be found online at https://www.mdpi.com/article/10.3390/proteomes13030032/s1, Figure S1: The original gel images, unsupervised spot details and a representative 2D-gel electrophoresis showing the spot numbers of significant DAPs (cutoffs; FC > 1.5, ANOVA FDR *p* ≤ 0.05) identified on the 2D-DIGE gel between RA and control. The numbered spots were excised and taken for identification via MALDI-TOF-MS. MW—protein molecular weight; pI—isoelectric point; Table S1: Mass spectrometry list of significant differentially abundant proteins between RA and control states identified in samples using 2D-DIGE. Protein name, accession number, Mascot score, MS% coverage, protein MW, and pI values according to the Uniprot database are listed; Table S2: Using mass spectrometry, the table shows the list of differentially regulated proteins between patients with RA and controls with their respective fold changes and significance levels (ANOVA, FDR *p*-value < 0.05). (Analysis type: MALDI-TOF; Mascot score greater than 56, Mascot search algorithm (v2.0.04, Matrix Science Ltd., London, UK), database: SwissProt; taxonomy: Homo sapiens); Table S3: The proteins were involved in cellular and metabolic processes and biological regulation and localized mainly within cellular anatomical structures and protein-containing complexes with corresponding FDR-adjusted *p*-values; Table S4: Experimental conditions for the validation of the selected proteins signature peptides.
