# Supplementary material for: Proteomic Profiling Reveals Novel Molecular Insights into Dysregulated Proteins in Established Cases of Rheumatoid Arthritis
Source: Proteomes. 2025 Jul 4;13(3):32. doi: 10.3390/proteomes13030032 (PMC12286002; doi:10.3390/proteomes13030032)
Supplement: Supplementary file 1 [file proteomes-13-00032-s001.zip › supplementary materials.pdf]

Figure S1: A representative 2D-gel electrophoresis shows the spot numbers of significant DAPs (cutoffs; FC > 1.5, ANOVA FDR p ⩽ 0.05) identified on the 2D-DIGE gel between RA and control. The numbered spots were excised and taken for identification via MALDI-TOF-MS. MW—protein molecular weight; pI—isoelectric point

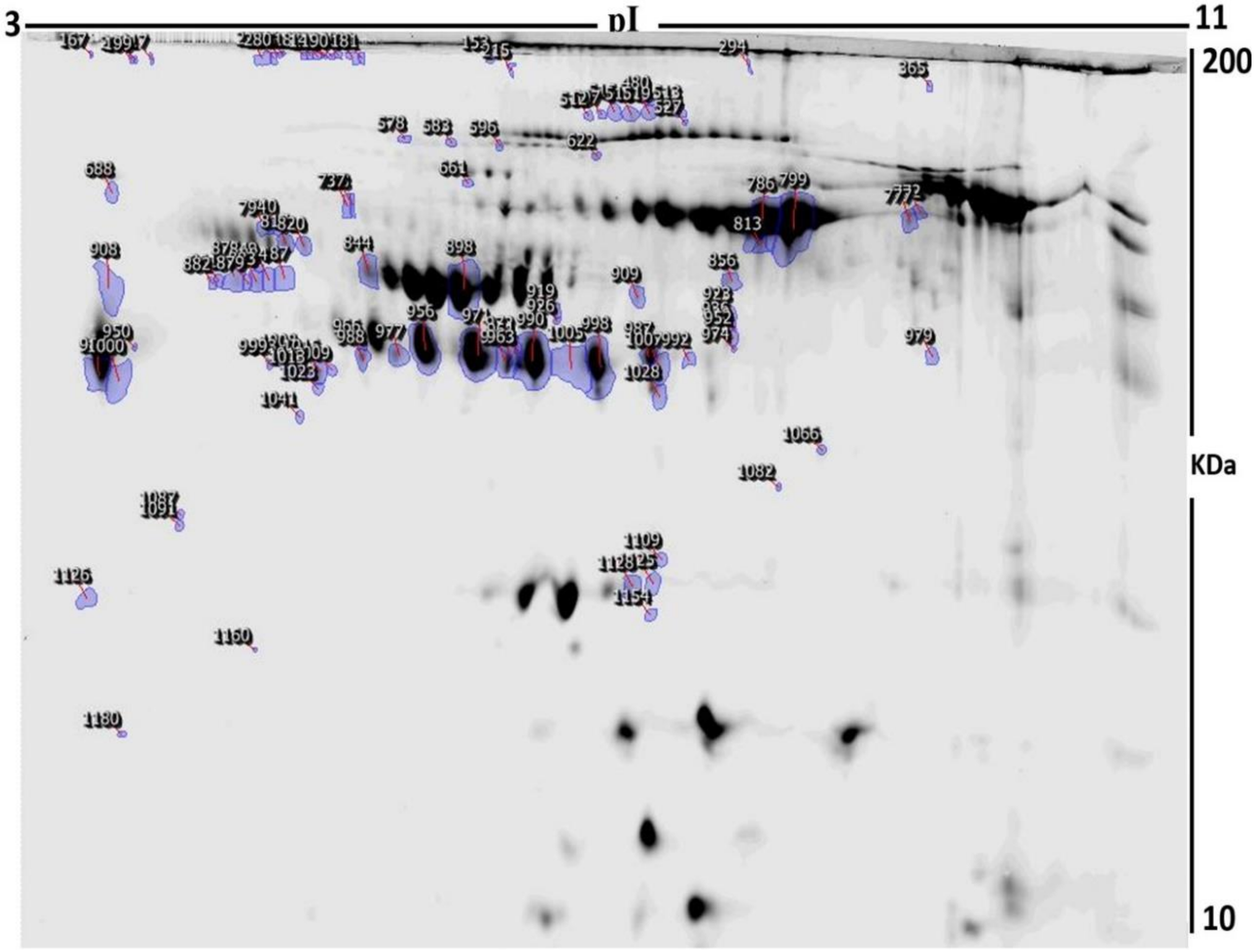

Supplementary Table S1: Mass spectrometry list of significant differentially abundant proteins between RA and control states identified in samples, using 2D-DIGE with. Protein name, accession number, Mascot score, MS % coverage, protein MW and pI values according to Uniprot database are listed.

S.NO	Spot Noa	Accession No[b]	Protein Name	MASCOT ID	Pi[c]	MW[d]	Cov%	Score
1	1016	Q96CT7	Coiled-coil domain-containing protein 124	CC124_HUMAN	9.54	25820	37	59
2	1109	P02647	Apolipoprotein A-I	APOA1_HUMAN	5.56	30759	65	146
3	1008	P02818	Osteocalcin	OSTCN_HUMAN	4.19	2935	95	59
4	977	P02818	Osteocalcin	OSTCN_HUMAN	4.19	2935	95	62
5	1013	P02795	Metallothionein-2	MT2_HUMAN	8.23	7178	36	60
6	1087	Q9P2M1	LRP2-binding protein	LR2BP_HUMAN	7.99	40154	12	57
7	952	P00738	Haptoglobin	HPT_HUMAN	6.13	45861	22	85
8	919	Q9UL63	Muskelin	MKLN1_HUMAN	5.91	85911	18	59
9	887	Q9UBX3	Mitochondrial dicarboxylate carrier	DIC_HUMAN	9.62	31718	27	62
10	963	P06727	Apolipoprotein A-IV	APOA4_HUMAN	5.28	45371	41	80
11	961	P06727	Apolipoprotein A-IV	APOA4_HUMAN	5.28	45371	42	105
12	997	P02818	Osteocalcin	OSTCN_HUMAN	4.19	2935	95	59
13	909	P02647	Apolipoprotein A-I	APOA1_HUMAN	5.56	30759	65	146

14	993	Q9Y240	C-type lectin domain family 11 member A	CLC11_HUMAN	5.06	36015	21	62
15	1091	Q9H4B6	Protein salvador homolog 1	SAV1_HUMAN	9.12	44720	15	59
16	983	P00738	Haptoglobin	HPT_HUMAN	6.13	45861	31	67
17	988	P46939	Utrophin	UTRN_HUMAN	5.2	396444	7	59
18	940	P00738	Haptoglobin	HPT_HUMAN	6.13	45861	22	70
19	926	O43715	TP53-regulated inhibitor of apoptosis 1	TRIA1_HUMAN	5.37	9008	40	60
20	519	Q3ZCN5	Otogelin-like protein	OTOGL_HUMAN	4.72	85249	25	68
21	972	Q96MM7	Heparan-sulfate 6-O-sulfotransferase 2	H6ST2_HUMAN	9.79	70055	12	59
22	518	P04217	Alpha-1B-glycoprotein	A1BG_HUMAN	5.58	54809	30	108
23	998	P00738	Haptoglobin	HPT_HUMAN	6.13	45861	29	59
24	1000	A0A0C4DH73	Immunoglobulin kappa variable 1-12	KV112_HUMAN	8.96	12233	46	61
25	990	P00738	Haptoglobin	HPT_HUMAN	6.13	45861	25	60
26	1012	O95613	Pericentrin	PCNT_HUMAN	5.39	380644	12	61
27	971	P00738	Haptoglobin	HPT_HUMAN	6.13	45861	27	59
28	966	Q92522	Histone H1.10	H1X_HUMAN	10.76	22474	34	76
29	879	Q9BXS6	Nucleolar and spindle-associated protein 1	NUSAP_HUMAN	9.92	49593	30	59
30	688	Q9H7D0	Dedicator of cytokinesis protein 5	DOCK5_HUMAN	8.08	216198	16	65

31	1023	Q92522	Histone H1.10	H1X_HUMAN	10.76	22474	27	62
32	861	P02790	Hemopexin	HEMO_HUMAN	6.55	52385	34	105
33	515	P01011	Alpha-1-antichymotrypsin	AACT_HUMAN	5.33	477792	43	100
34	882	Q9Y3D5	28S ribosomal protein S18c, mitochondrial	RT18C_HUMAN	9.63	16238	33	60
35	1154	P13647	Keratin, type II cytoskeletal 5	K2C5_HUMAN	7.59	62568	14	59
36	1066	P02647	Apolipoprotein A-I	APOA1_HUMAN	5.56	30759	39	113
37	875	Q9Y3D5	28S ribosomal protein S18c, mitochondrial	RT18C_HUMAN	9.63	16238	24	60
38	979	O95613	Pericentrin	PCNT_HUMAN	5.39	380644	13	66
39	507	Q02790	Peptidyl-prolyl cis-trans isomerase FKBP4	FKBP4_HUMAN	5.35	52057	16	61
40	215	A5D8V7 ·	Coiled-coil domain-containing protein 151	ODAD3_HUMAN	9.11	69268	15	59
41	884	Q3ZCN5	Otogelin-like protein	OTOGL_HUMAN	5.61	39460	20	59
42	898	P01009	Alpha-1-antitrypsin	A1AT_HUMAN	5.37	46878	39	63
43	480	P02768	Albumin	ALBU_HUMAN	5.92	71317	46	101
44	786	P02787	Serotransferrin	TRFE_HUMAN	6.81	79280	44	157

45	1028	O15382	Branched-chain-amino-acid aminotransferase, mitochondrial	BCAT2_HUMAN	8.88	44658	13	62
46	820	Q15131	Cyclin-dependent kinase 10	CDK10_HUMAN	9.06	41582	26	72
47	956	P00738	Haptoglobin	HPT_HUMAN	6.13	45861	37	96
48	817	Q9HBE4	Interleukin-21	IL21_HUMAN	9.42	18196	52	59
49	1128	Q8NBN7	Retinol dehydrogenase 13	RDH13_HUMAN	8.23	36195	14	63
50	583	O95613	Pericentrin	PCNT_HUMAN	5.39	380644	14	59
51	992	P00738	Haptoglobin	HPT_HUMAN	6.13	45861	36	95
52	758	P60763	Ras-related C3 botulinum toxin substrate 3	RAC3_HUMAN	8.43	21764	29	59
53	974	P00738	Haptoglobin	HPT_HUMAN	6.13	45861	37	96
54	844	P01009	Alpha-1-antitrypsin	A1AT_HUMAN	5.37	46878	40	94
55	1005	P06727	Apolipoprotein A-IV	APOA4_HUMAN	5.28	45371	59	192
56	728	P01011	Alpha-1-antichymotrypsin	AACT_HUMAN	5.33	477792	31	76
57	794	P02768	Albumin	ALBU_HUMAN	5.92	71317	31	58
58	790	Q9P126	C-type lectin domain family 1 member B	CLC1B_HUMAN	8.79	27091	36	59
59	1125	P02647	Apolipoprotein A-I	APOA1_HUMAN	5.56	30759	37	68

60	661	Q9UBI4	Stomatin-like protein 1	STML1_HUMAN	8.18	43283	20	59
61	883	Q9Y240	C-type lectin domain family 11 member A	CLC11_HUMAN	12	13693	56	60
62	964	Q9Y3D5	28S ribosomal protein S18c, mitochondrial	RT18C_HUMAN	9.63	16238	50	64
63	923	Q7Z2K8	G protein-regulated inducer of neurite outgrowth	GRIN1_HUMAN	8.33	103106	15	59
64	512	Q92522	Histone H1.10	H1X_HUMAN	10.76	22474	31	59
65	987	P00738	Haptoglobin	HPT_HUMAN	6.13	45861	23	64
66	527	Q8N806	Putative E3 ubiquitin-protein ligase UBR7	UBR7_HUMAN	4.7	49336	17	66
67	856	Q9NWK9	Box C/D snoRNA protein 1	BCD1_HUMAN	5.62	54511	30	54
68	849	Q6ZN19	Zinc finger protein 841	ZN841_HUMAN	9.48	95939	28	69
69	513	Q8IY21	Probable ATP-dependent RNA helicase DDX60	DDX60_HUMAN	7.65	199680	12	59

Supplementary Table S2: Using mass spectrometry, the table shows the list of differentially regulated proteins between patients with RA and controls with their respective fold changes and significance levels (ANOVA, FDRp-value < 0.05). [Analysis type: MALDI-TOF; Mascot score greater than 56, Mascot search algorithm (v2.0.04, Matrix Science Ltd., UK), database: SwissProt; taxonomy: Homo sapiens].

SL. No	Spot No[a]	Accession No	Protein Name	MASCOT ID	FDR P-value[b] (ANOVA)	Ratio[c] RA/Control	Exp[d]
1	1016	Q96CT7	Coiled-coil domain-containing protein 124	CC124	3.40E-06	1.5	DOWN
2	1109	P02647	Apolipoprotein A-I	APOA1	1.00E-04	1.5	DOWN
3	1008	P02818	Osteocalcin	OSTCN	1.00E-04	1.5	DOWN
4	977	P02818	Osteocalcin	OSTCN	1.00E-04	1.8	DOWN
5	1013	P02795	Metallothionein-2	MT2	2.00E-04	1.5	DOWN
6	1087	Q9P2M1	LRP2-binding protein	LR2BP	2.00E-04	1.92	DOWN
7	952	P00738	Haptoglobin	HPT	4.00E-04	1.63	DOWN
8	919	Q9UL63	Muskelin	MKLN1	4.00E-04	1.92	DOWN
9	887	Q9UBX3	Mitochondrial dicarboxylate carrier	DIC	6.90E-04	1.59	UP
10	963	P06727	Apolipoprotein A-IV	APOA4	7.00E-04	1.57	DOWN
11	961	P06727	Apolipoprotein A-IV	APOA4	8.00E-04	1.65	DOWN
12	997	P02818	Osteocalcin	OSTCN	8.00E-04	1.5	DOWN
13	909	P02647	Apolipoprotein A-I	APOA1	9.00E-04	1.5	DOWN
14	993	Q9Y240	C-type lectin domain family 11 member A	CLC11	1.00E-03	1.5	DOWN
15	1091	Q9H4B6	Protein salvador homolog 1	SAV1	1.00E-03	1.5	DOWN
16	983	P00738	Haptoglobin	HPT	1.00E-03	1.56	DOWN
17	988	P46939	Utrophin	UTRN	2.00E-03	1.53	DOWN
18	940	P00738	Haptoglobin	HPT	2.00E-03	1.5	DOWN
19	926	O43715	TP53-regulated inhibitor of apoptosis 1	TRIA1	2.00E-03	1.5	DOWN
20	519	Q3ZCN5	Otogelin-like protein	OTOGL	2.00E-03	2.1	DOWN
21	972	Q96MM7	Heparan-sulfate 6-O-sulfotransferase 2	H6ST2	2.00E-03	1.6	DOWN
22	518	P04217	Alpha-1B-glycoprotein	A1BG	2.00E-03	1.5	DOWN
23	998	P00738	Haptoglobin	HPT	3.00E-03	1.57	DOWN
24	1000	A0A0C4DH73	Immunoglobulin kappa variable 1-12	KV112	4.00E-03	1.5	DOWN

SL. No	Spot No[a]	Accession No	Protein Name	MASCOT ID	FDR P-value[b] (ANOVA)	Ratio[c] RA/Control	Exp[d]
25	990	P00738	Haptoglobin	HPT	5.00E-03	1.56	DOWN
26	1012	O95613	Pericentrin	PCNT	5.00E-03	1.56	DOWN
27	971	P00738	Haptoglobin	HPT	6.00E-03	1.53	DOWN
28	966	Q92522	Histone H1.10	H1X	9.00E-03	1.63	DOWN
29	879	Q9BXS6	Nucleolar and spindle-associated protein 1	NUSAP	0.01	1.65	UP
30	688	Q9H7D0	Dedicator of cytokinesis protein 5	DOCK5	0.01	1.74	DOWN
31	1023	Q92522	Histone H1.10	H1X	0.011	1.58	DOWN
32	861	P02790	Hemopexin	HEMO	0.013	1.9	UP
33	515	P01011	Alpha-1-antichymotrypsin	AACT	0.015	1.55	DOWN
34	882	Q9Y3D5	28S ribosomal protein S18c, mitochondrial	RT18C	0.015	1.58	UP
35	1154	P13647	Keratin, type II cytoskeletal 5	K2C5	0.017	1.5	DOWN
36	1066	P02647	Apolipoprotein A-I	APOA1	0.018	1.52	DOWN
37	875	Q9Y3D5	28S ribosomal protein S18c, mitochondrial	RT18C	0.018	1.5	UP
38	979	O95613	Pericentrin	PCNT	0.019	1.5	DOWN
39	507	Q02790	Peptidyl-prolyl cis-trans isomerase FKBP4	FKBP4	0.02	1.59	DOWN
40	215	A5D8V7 ·	Coiled-coil domain-containing protein 151	ODAD3	0.022	1.5	UP
41	884	Q3ZCN5	Otogelin-like protein	OTOGL	0.022	1.5	UP
42	898	P01009	Alpha-1-antitrypsin	A1AT	0.023	1.69	DOWN
43	480	P02768	Albumin	ALBU	0.028	1.72	DOWN
44	786	P02787	Serotransferrin	TRFE	0.029	1.66	DOWN
45	1028	O15382	Branched-chain-amino-acid aminotransferase, mitochondrial	BCAT2	0.029	1.66	DOWN
46	820	Q15131	Cyclin-dependent kinase 10	CDK10	0.029	1.68	DOWN
47	956	P00738	Haptoglobin	HPT	0.03	1.61	DOWN
48	817	Q9HBE4	Interleukin-21	IL21	0.03	1.66	DOWN
49	1128	Q8NBN7	Retinol dehydrogenase 13	RDH13	0.034	1.5	DOWN
50	583	O95613	Pericentrin	PCNT	0.034	1.98	UP
51	992	P00738	Haptoglobin	HPT	0.035	1.6	DOWN
52	758	P60763	Ras-related C3 botulinum toxin substrate 3	RAC3	0.037	1.7	DOWN
53	974	P00738	Haptoglobin	HPT	0.038	1.5	DOWN

SL. No	Spot No[a]	Accession No	Protein Name	MASCOT ID	FDR P-value[b] (ANOVA)	Ratio[c] RA/Control	Exp[d]
54	844	P01009	Alpha-1-antitrypsin	A1AT	0.041	1.5	UP
55	1005	P06727	Apolipoprotein A-IV	APOA4	0.042	1.61	DOWN
56	728	P01011	Alpha-1-antichymotrypsin	AACT	0.042	1.57	UP
57	794	P02768	Albumin	ALBU	0.044	1.69	DOWN
58	790	Q9P126	C-type lectin domain family 1 member B	CLC1B	0.045	1.74	DOWN
59	1125	P02647	Apolipoprotein A-I	APOA1	0.046	1.65	DOWN
60	661	Q9UBI4	Stomatin-like protein 1	STML1	0.047	1.75	DOWN
61	883	Q9Y240	C-type lectin domain family 11 member A	CLC11	0.047	1.51	UP
62	964	Q9Y3D5	28S ribosomal protein S18c, mitochondrial	RT18C	0.048	1.5	DOWN
63	923	Q7Z2K8	G protein-regulated inducer of neurite outgrowth 1	GRIN1	0.05	1.51	DOWN
64	512	Q92522	Histone H1.10	H1X	0.05	1.69	DOWN
65	987	P00738	Haptoglobin	HPT	0.052	1.72	DOWN
66	527	Q8N806	Putative E3 ubiquitin-protein ligase UBR7	UBR7	0.052	1.5	DOWN
67	856	Q9NWK9	Box C/D snoRNA protein 1	BCD1	0.053	1.57	DOWN
68	849	Q6ZN19	Zinc finger protein 841	ZN841	0.055	1.5	UP
69	513	Q8IY21	Probable ATP-dependent RNA helicase DDX60	DDX60	0.058	1.57	DOWN

[a] Protein accession number for SWISSPROT Database.

[b] P-Value (ANOVA).

[c] Ratio between the groups

[d] Protein expression between the groups

Table S3 A list of the overrepresented Biological Processes, Molecular Function and Cellular locations identified using PANTHER for the statistically significantly abundant proteins, fold change and their respective FDR values.

**GO Biological Process**	**Homo sapiens (REF)**	**Number of Mapped proteins**	**Fold Enrichment**	**+/-**	**raw P value**	**FDR**
regulation of cell-substrate adhesion	219	5	9.04	+	2.26E-04	3.71E-01
defense response	1515	12	3.13	+	3.15E-04	3.89E-01
regulation of vascular associated smooth muscle contraction	10	2	79.15	+	2.78E-04	4.12E-01
regulation of intestinal cholesterol absorption	9	2	87.95	+	2.23E-04	4.13E-01
positive regulation of cell-substrate adhesion	128	4	12.37	+	3.07E-04	4.13E-01
regulation of humoral immune response	45	3	26.38	+	2.00E-04	4.24E-01
regulation of lipid metabolic process	331	6	7.17	+	1.81E-04	4.46E-01
regulation of intestinal lipid absorption	12	2	65.96	+	4.07E-04	4.63E-01
regulation of intestinal absorption	15	2	52.77	+	6.44E-04	4.77E-01
regulation of substrate adhesion-dependent cell spreading	61	3	19.46	+	4.94E-04	4.88E-01
detoxification	151	4	10.48	+	5.73E-04	5.00E-01
regulation of phospholipid transport	15	2	52.77	+	6.44E-04	5.02E-01
phospholipid efflux	13	2	60.89	+	4.80E-04	5.08E-01
regulation of lipid localization	161	4	9.83	+	7.29E-04	5.15E-01
regulation of triglyceride catabolic process	14	2	56.54	+	5.59E-04	5.18E-01
positive regulation of substrate adhesion-dependent cell spreading	43	3	27.61	+	1.75E-04	5.18E-01
positive regulation of phospholipid transport	15	2	52.77	+	6.44E-04	5.30E-01
acute inflammatory response	73	3	16.26	+	8.35E-04	5.38E-01
reverse cholesterol transport	17	2	46.56	+	8.31E-04	5.60E-01
response to stimulus	8280	33	1.58	+	9.39E-04	5.80E-01
homeostatic process	1493	11	2.92	+	1.07E-03	6.10E-01
response to external stimulus	1981	13	2.6	+	1.03E-03	6.10E-01

regulation of defense response	845	8	3.75	+	1.18E-03	6.26E-01
high-density lipoprotein particle remodeling	21	2	37.69	+	1.28E-03	6.30E-01
regulation of complement activation	20	2	39.58	+	1.16E-03	6.34E-01
regulation of triglyceride metabolic process	43	3	27.61	+	1.75E-04	6.48E-01
positive regulation of humoral immune response	21	2	37.69	+	1.28E-03	6.51E-01
positive regulation of triglyceride metabolic process	22	2	35.98	+	1.40E-03	6.69E-01
positive regulation of cell adhesion	499	6	4.76	+	1.56E-03	6.80E-01
plasma lipoprotein particle assembly	24	2	32.98	+	1.67E-03	6.86E-01
regulation of heterotypic cell-cell adhesion	23	2	34.41	+	1.53E-03	6.88E-01
regulation of production of molecular mediator of immune response	200	4	7.92	+	1.63E-03	6.89E-01
cellular oxidant detoxification	90	3	13.19	+	1.53E-03	7.09E-01
positive regulation of lipid catabolic process	27	2	29.32	+	2.11E-03	7.27E-01
keratinocyte apoptotic process	1	1	> 100	+	2.53E-03	7.34E-01
lipid localization	357	5	5.54	+	2.03E-03	7.35E-01
regulation of interleukin-1 beta production	96	3	12.37	+	1.84E-03	7.38E-01
cholesterol efflux	30	2	26.38	+	2.60E-03	7.42E-01
Rac protein signal transduction	26	2	30.44	+	1.96E-03	7.44E-01
lipid transport	356	5	5.56	+	2.01E-03	7.44E-01
protein-lipid complex assembly	27	2	29.32	+	2.11E-03	7.45E-01
thiosulfate transport	1	1	> 100	+	2.53E-03	7.49E-01
positive regulation of lipid localization	103	3	11.53	+	2.25E-03	7.58E-01
transition metal ion transport	98	3	12.12	+	1.95E-03	7.62E-01
phospholipid transport	104	3	11.42	+	2.31E-03	7.62E-01
vibrational conductance of sound to the inner ear	1	1	> 100	+	2.53E-03	7.64E-01
negative regulation of interleukin-1 beta production	31	2	25.53	+	2.78E-03	7.77E-01
intermediate filament polymerization	1	1	> 100	+	2.53E-03	7.80E-01

regulation of immune system process	1653	11	2.63	+	2.42E-03	7.80E-01
steroid hormone receptor complex assembly	1	1	> 100	+	2.53E-03	7.97E-01
tissue homeostasis	236	4	6.71	+	2.97E-03	7.99E-01
anatomical structure homeostasis	236	4	6.71	+	2.97E-03	8.14E-01
regulation of interleukin-1 production	115	3	10.32	+	3.08E-03	8.14E-01
cellular detoxification	116	3	10.24	+	3.15E-03	8.20E-01
arabinose metabolic process	2	1	> 100	+	5.05E-03	8.40E-01
L-arabinose metabolic process	2	1	> 100	+	5.05E-03	8.50E-01
negative regulation of cytokine production involved in immune response	34	2	23.28	+	3.34E-03	8.52E-01
positive regulation of B cell mediated immunity	43	2	18.41	+	5.29E-03	8.52E-01
response to peptide	857	7	3.23	+	5.54E-03	8.54E-01
positive regulation of triglyceride catabolic process	8	2	98.94	+	1.74E-04	8.58E-01
regulation of plasma lipoprotein oxidation	2	1	> 100	+	5.05E-03	8.60E-01
positive regulation of immunoglobulin mediated immune response	43	2	18.41	+	5.29E-03	8.62E-01
acylglycerol homeostasis	44	2	17.99	+	5.54E-03	8.63E-01
response to stress	3539	17	1.9	+	5.45E-03	8.69E-01
defense response to other organism	1076	8	2.94	+	5.28E-03	8.69E-01
negative regulation of plasma lipoprotein oxidation	2	1	> 100	+	5.05E-03	8.70E-01
regulation of digestive system process	44	2	17.99	+	5.54E-03	8.72E-01
negative regulation of production of molecular mediator of immune response	45	2	17.59	+	5.78E-03	8.74E-01
branched-chain amino acid biosynthetic process	2	1	> 100	+	5.05E-03	8.80E-01
bone remodeling	45	2	17.59	+	5.78E-03	8.83E-01
neurotransmitter receptor transport postsynaptic membrane to endosome	2	1	> 100	+	5.05E-03	8.90E-01
multicellular organismal-level homeostasis	658	6	3.61	+	6.10E-03	8.94E-01

regulation of transport	1599	10	2.48	+	6.07E-03	8.99E-01
negative regulation of hydrogen peroxide catabolic process	2	1	> 100	+	5.05E-03	9.01E-01
superoxide metabolic process	46	2	17.21	+	6.04E-03	9.03E-01
regulation of response to external stimulus	1108	8	2.86	+	6.28E-03	9.12E-01
intermediate filament polymerization or depolymerization	2	1	> 100	+	5.05E-03	9.12E-01
regulation of receptor-mediated endocytosis	122	3	9.73	+	3.64E-03	9.13E-01
oxaloacetate(2-) transmembrane transport	2	1	> 100	+	5.05E-03	9.23E-01
negative regulation of superoxide anion generation	2	1	> 100	+	5.05E-03	9.35E-01
protein-lipid complex remodeling	37	2	21.39	+	3.94E-03	9.42E-01
regulation of complement activation, lectin pathway	2	1	> 100	+	5.05E-03	9.47E-01
Unclassified	2856	1	0.14	-	7.67E-03	9.47E-01
protein-containing complex remodeling	39	2	20.3	+	4.37E-03	9.52E-01
biological_process	17724	51	1.14	+	7.67E-03	9.55E-01
plasma lipoprotein particle remodeling	37	2	21.39	+	3.94E-03	9.57E-01
negative regulation of complement activation, lectin pathway	2	1	> 100	+	5.05E-03	9.59E-01
positive regulation of endocytosis	161	3	7.37	+	7.85E-03	9.61E-01
retinol metabolic process	52	2	15.22	+	7.66E-03	9.62E-01
response to toxic substance	255	4	6.21	+	3.91E-03	9.66E-01
negative regulation of interleukin-1 production	39	2	20.3	+	4.37E-03	9.67E-01
regulation of endocytosis	308	4	5.14	+	7.58E-03	9.68E-01
plasma lipoprotein particle organization	52	2	15.22	+	7.66E-03	9.70E-01
negative regulation of bone development	2	1	> 100	+	5.05E-03	9.71E-01
positive regulation of immune effector process	270	4	5.86	+	4.79E-03	9.71E-01
negative regulation of immune effector process	134	3	8.86	+	4.73E-03	9.73E-01
alcohol metabolic process	313	4	5.06	+	8.01E-03	9.73E-01

syncytiotrophoblast cell differentiation involved in labyrinthine layer development	3	1	> 100	+	7.56E-03	9.74E-01
regulation of sterol transport	60	2	13.19	+	1.01E-02	9.77E-01
regulation of cytokine production involved in immune response	128	3	9.28	+	4.16E-03	9.78E-01
positive regulation of lymphocyte mediated immunity	133	3	8.93	+	4.63E-03	9.80E-01
negative regulation of reactive oxygen species metabolic process	39	2	20.3	+	4.37E-03	9.81E-01
response to cytokine	840	7	3.3	+	4.97E-03	9.82E-01
complement-dependent cytotoxicity	3	1	> 100	+	7.56E-03	9.83E-01
regulation of cholesterol transport	60	2	13.19	+	1.01E-02	9.84E-01
positive regulation of activation of membrane attack complex	2	1	> 100	+	5.05E-03	9.84E-01
regulation of lipid transport	136	3	8.73	+	4.93E-03	9.86E-01
positive regulation of lipid metabolic process	134	3	8.86	+	4.73E-03	9.86E-01
chromosome condensation	40	2	19.79	+	4.59E-03	9.86E-01
chylomicron remodeling	4	1	98.94	+	1.01E-02	9.88E-01
cellular response to toxic substance	130	3	9.13	+	4.34E-03	9.90E-01
vertebrate eye-specific patterning	3	1	> 100	+	7.56E-03	9.91E-01
response to zinc ion	54	2	14.66	+	8.24E-03	9.92E-01
chylomicron assembly	4	1	98.94	+	1.01E-02	9.95E-01
monoatomic ion transmembrane transport	859	1	0.46	-	7.26E-01	1.00E+00
respiratory burst involved in defense response	10	0	< 0.01	-	1.00E+00	1.00E+00
response to lead ion	19	0	< 0.01	-	1.00E+00	1.00E+00
**GO Molecular Function**						
phosphatidylcholine-sterol O-acyltransferase activator activity	6	2	> 100	+	9.33E-05	4.73E-01
macrolide binding	12	2	65.96	+	4.07E-04	6.87E-01
ferric iron binding	10	2	79.15	+	2.78E-04	7.05E-01

lipid transfer activity	66	3	17.99	+	6.22E-04	7.89E-01
butanol dehydrogenase (NAD+) activity	1	1	> 100	+	2.53E-03	8.01E-01
peptidase regulator activity	236	4	6.71	+	2.97E-03	8.35E-01
thiosulfate transmembrane transporter activity	1	1	> 100	+	2.53E-03	8.54E-01
peptidase inhibitor activity	177	4	8.94	+	1.04E-03	8.77E-01
sterol transfer activity	32	2	24.74	+	2.96E-03	8.83E-01
endopeptidase inhibitor activity	169	4	9.37	+	8.74E-04	8.86E-01
antioxidant activity	84	3	14.13	+	1.25E-03	9.09E-01
C5a anaphylatoxin chemotactic receptor binding	1	1	> 100	+	2.53E-03	9.15E-01
cholesterol transfer activity	25	2	31.66	+	1.81E-03	9.18E-01
oxaloacetate transmembrane transporter activity	2	1	> 100	+	5.05E-03	9.84E-01
C5L2 anaphylatoxin chemotactic receptor binding	1	1	> 100	+	2.53E-03	9.86E-01
endopeptidase regulator activity	198	4	8	+	1.57E-03	9.94E-01
growth hormone receptor binding	10	0	< 0.01	-	1.00E+00	1.00E+00
granulocyte colony-stimulating factor receptor binding	1	0	< 0.01	-	1.00E+00	1.00E+00
14-3-3 protein binding	32	0	< 0.01	-	1.00E+00	1.00E+00
neuromedin U binding	2	0	< 0.01	-	1.00E+00	1.00E+00
neuropeptide binding	33	0	< 0.01	-	1.00E+00	1.00E+00
neuromedin U receptor binding	1	0	< 0.01	-	1.00E+00	1.00E+00
1-(4-iodo-2,5-dimethoxyphenyl)propan-2-amine binding	2	0	< 0.01	-	1.00E+00	1.00E+00
N-terminal protein N-methyltransferase activity	2	0	< 0.01	-	1.00E+00	1.00E+00
granulocyte macrophage colony-stimulating factor receptor binding	1	0	< 0.01	-	1.00E+00	1.00E+00
erythropoietin receptor binding	3	0	< 0.01	-	1.00E+00	1.00E+00
ciliary neurotrophic factor receptor binding	6	0	< 0.01	-	1.00E+00	1.00E+00
cytokine receptor binding	274	2	2.89	+	1.52E-01	1.00E+00
cytokine activity	237	1	1.67	+	4.53E-01	1.00E+00
scavenger receptor binding	4	0	< 0.01	-	1.00E+00	1.00E+00

death receptor binding	21	1	18.85	+	5.18E-02	1.00E+00
Toll binding	1	0	< 0.01	-	1.00E+00	1.00E+00
lactosylceramide 4-alpha-galactosyltransferase activity	1	0	< 0.01	-	1.00E+00	1.00E+00
N-acetylgalactosaminyl-proteoglycan 3-beta-glucuronosyltransferase activity	4	0	< 0.01	-	1.00E+00	1.00E+00
xenobiotic transmembrane transporter activity	32	0	< 0.01	-	1.00E+00	1.00E+00
inositol trisphosphate phosphatase activity	14	0	< 0.01	-	1.00E+00	1.00E+00
smoothened binding	4	0	< 0.01	-	1.00E+00	1.00E+00
Tat protein binding	10	0	< 0.01	-	1.00E+00	1.00E+00
potassium ion binding	13	0	< 0.01	-	1.00E+00	1.00E+00
receptor tyrosine kinase-like orphan receptor binding	1	0	< 0.01	-	1.00E+00	1.00E+00
type II transforming growth factor beta receptor binding	11	0	< 0.01	-	1.00E+00	1.00E+00
N-acetylglucosaminyl-proteoglycan 4-beta-glucuronosyltransferase activity	2	0	< 0.01	-	1.00E+00	1.00E+00
patched binding	8	0	< 0.01	-	1.00E+00	1.00E+00
glucuronosyl-N-acetylglucosaminyl-proteoglycan 4-alpha-N-acetylglucosaminyltransferase activity	3	0	< 0.01	-	1.00E+00	1.00E+00
Notch binding	26	0	< 0.01	-	1.00E+00	1.00E+00
type 2 fibroblast growth factor receptor binding	5	0	< 0.01	-	1.00E+00	1.00E+00
hyaluronan synthase activity	3	0	< 0.01	-	1.00E+00	1.00E+00
tubulin deacetylase activity	2	0	< 0.01	-	1.00E+00	1.00E+00
phospholipid:diacylglycerol acyltransferase activity	1	0	< 0.01	-	1.00E+00	1.00E+00
frizzled binding	40	0	< 0.01	-	1.00E+00	1.00E+00
type 1 fibroblast growth factor receptor binding	6	0	< 0.01	-	1.00E+00	1.00E+00
mitochondrion targeting sequence binding	5	0	< 0.01	-	1.00E+00	1.00E+00
fibroblast growth factor receptor binding	27	0	< 0.01	-	1.00E+00	1.00E+00
endoplasmic reticulum signal peptide binding	3	0	< 0.01	-	1.00E+00	1.00E+00
signaling receptor binding	1544	9	2.31	+	1.44E-02	1.00E+00

glycerol-3-phosphate dehydrogenase (NAD+) activity	2	0	< 0.01	-	1.00E+00	1.00E+00
5'-3' RNA polymerase activity	33	0	< 0.01	-	1.00E+00	1.00E+00
DNA polymerase activity	40	0	< 0.01	-	1.00E+00	1.00E+00
neuropeptide receptor binding	41	0	< 0.01	-	1.00E+00	1.00E+00
oxidoreductase activity, acting on peroxide as acceptor	58	0	< 0.01	-	1.00E+00	1.00E+00
enoyl-[acyl-carrier-protein] reductase (NADPH) activity	2	0	< 0.01	-	1.00E+00	1.00E+00
intracellularly calcium-gated channel activity	19	0	< 0.01	-	1.00E+00	1.00E+00
RNA strand-exchange activity	2	0	< 0.01	-	1.00E+00	1.00E+00
estrogen response element binding	11	0	< 0.01	-	1.00E+00	1.00E+00
benzoate-CoA ligase activity	3	0	< 0.01	-	1.00E+00	1.00E+00
oxidoreductase activity, acting on diphenols and related substances as donors	9	0	< 0.01	-	1.00E+00	1.00E+00
oxidoreductase activity, acting on a heme group of donors	14	0	< 0.01	-	1.00E+00	1.00E+00
oxidoreductase activity, acting on a sulfur group of donors, quinone or similar compound as acceptor	3	0	< 0.01	-	1.00E+00	1.00E+00
oxidoreductase activity, acting on a sulfur group of donors, disulfide as acceptor	8	0	< 0.01	-	1.00E+00	1.00E+00
oxidoreductase activity, acting on a sulfur group of donors, oxygen as acceptor	11	0	< 0.01	-	1.00E+00	1.00E+00
XTP diphosphatase activity	1	0	< 0.01	-	1.00E+00	1.00E+00
UTP diphosphatase activity	3	0	< 0.01	-	1.00E+00	1.00E+00
ITP diphosphatase activity	1	0	< 0.01	-	1.00E+00	1.00E+00
poly(G) binding	8	0	< 0.01	-	1.00E+00	1.00E+00
diphthine methyl ester synthase activity	1	0	< 0.01	-	1.00E+00	1.00E+00
DNA N6-methyladenine demethylase activity	2	0	< 0.01	-	1.00E+00	1.00E+00
ABC-type sterol transporter activity	2	0	< 0.01	-	1.00E+00	1.00E+00

ATPase-coupled lipid transmembrane transporter activity	9	0	< 0.01	-	1.00E+00	1.00E+00
HMG box domain binding	12	0	< 0.01	-	1.00E+00	1.00E+00
oxidoreductase activity, acting on a sulfur group of donors, NAD(P) as acceptor	11	0	< 0.01	-	1.00E+00	1.00E+00
oxidoreductase activity, acting on a sulfur group of donors	55	0	< 0.01	-	1.00E+00	1.00E+00
oxidoreductase activity, acting on other nitrogenous compounds as donors, cytochrome as acceptor	4	0	< 0.01	-	1.00E+00	1.00E+00
GTP diphosphatase activity	2	0	< 0.01	-	1.00E+00	1.00E+00
oxidoreductase activity, acting on other nitrogenous compounds as donors	12	0	< 0.01	-	1.00E+00	1.00E+00
dTTP diphosphatase activity	1	0	< 0.01	-	1.00E+00	1.00E+00
8-oxo-7,8-dihydroguanine DNA N-glycosylase activity	2	0	< 0.01	-	1.00E+00	1.00E+00
short-chain fatty acyl-CoA hydrolase activity	1	0	< 0.01	-	1.00E+00	1.00E+00
deoxyhypusine synthase activity	1	0	< 0.01	-	1.00E+00	1.00E+00
tRNA-queuosine(34) galactosyltransferase activity	1	0	< 0.01	-	1.00E+00	1.00E+00
TPR domain binding	8	0	< 0.01	-	1.00E+00	1.00E+00
N-box binding	1	0	< 0.01	-	1.00E+00	1.00E+00
oxidoreductase activity, acting on NAD(P)H, quinone or similar compound as acceptor	58	0	< 0.01	-	1.00E+00	1.00E+00
oxidoreductase activity, acting on NAD(P)H, heme protein as acceptor	11	0	< 0.01	-	1.00E+00	1.00E+00
oxidoreductase activity, acting on NAD(P)H as acceptor	1	0	< 0.01	-	1.00E+00	1.00E+00
oxidoreductase activity, acting on NAD(P)H	90	0	< 0.01	-	1.00E+00	1.00E+00
nitric oxide dioxygenase activity, heme protein as donor	1	0	< 0.01	-	1.00E+00	1.00E+00
diacylglycerol-dependent, calcium-independent	4	0	< 0.01	-	1.00E+00	1.00E+00

serine/threonine kinase activity						
calcium,diacylglycerol-dependent serine/threonine kinase activity	3	0	< 0.01	-	1.00E+00	1.00E+00
transporter inhibitor activity	52	0	< 0.01	-	1.00E+00	1.00E+00
diacylglycerol-dependent serine/threonine kinase activity	17	0	< 0.01	-	1.00E+00	1.00E+00
eukaryotic translation initiation factor 2alpha kinase activity	4	0	< 0.01	-	1.00E+00	1.00E+00
cyclin-dependent protein serine/threonine kinase activity	29	1	13.65	+	7.08E-02	1.00E+00
cGMP-dependent protein kinase activity	2	0	< 0.01	-	1.00E+00	1.00E+00
cAMP-dependent protein kinase activity	7	0	< 0.01	-	1.00E+00	1.00E+00
cyclic nucleotide-dependent protein kinase activity	9	0	< 0.01	-	1.00E+00	1.00E+00
protein-lipid complex binding	30	1	13.19	+	7.31E-02	1.00E+00
Delta4-3-oxosteroid 5beta-reductase activity	2	0	< 0.01	-	1.00E+00	1.00E+00
lipoprotein particle binding	30	1	13.19	+	7.31E-02	1.00E+00
oxidoreductase activity, acting on the CH-NH group of donors, quinone or similar compound as acceptor	4	0	< 0.01	-	1.00E+00	1.00E+00
oxidoreductase activity, acting on the CH-NH group of donors, oxygen as acceptor	3	0	< 0.01	-	1.00E+00	1.00E+00
cyanamide hydratase activity	2	0	< 0.01	-	1.00E+00	1.00E+00
oxidoreductase activity, acting on the CH-NH group of donors, NAD or NADP as acceptor	17	0	< 0.01	-	1.00E+00	1.00E+00
corticosterone 18-monooxygenase activity	2	0	< 0.01	-	1.00E+00	1.00E+00
oxidoreductase activity, acting on the CH-NH group of donors	26	0	< 0.01	-	1.00E+00	1.00E+00
oxidoreductase activity, acting on the CH-NH2 group of donors, disulfide as acceptor	1	0	< 0.01	-	1.00E+00	1.00E+00

oxidoreductase activity, acting on the CH-NH2 group of donors, oxygen as acceptor	16	0	< 0.01	-	1.00E+00	1.00E+00
transporter activator activity	26	0	< 0.01	-	1.00E+00	1.00E+00
transporter regulator activity	191	0	< 0.01	-	1.00E+00	1.00E+00
**GO Cellular Component**						
blood microparticle	144	10	27.48	+	2.52E-12	5.04E-09
vesicle lumen	330	9	10.79	+	1.26E-07	6.30E-05
cytoplasmic vesicle lumen	329	9	10.83	+	1.23E-07	8.19E-05
secretory granule lumen	324	9	10.99	+	1.08E-07	1.08E-04
platelet alpha granule lumen	67	5	29.54	+	7.25E-07	2.90E-04
platelet alpha granule	91	5	21.75	+	3.34E-06	1.11E-03
endoplasmic reticulum lumen	318	7	8.71	+	1.45E-05	4.14E-03
endocytic vesicle lumen	22	3	53.97	+	2.26E-05	5.66E-03
extracellular membrane-bounded organelle	2132	16	2.97	+	4.54E-05	7.57E-03
collagen-containing extracellular matrix	386	7	7.18	+	5.00E-05	7.69E-03
extracellular organelle	2132	16	2.97	+	4.54E-05	8.25E-03
extracellular exosome	2102	16	3.01	+	3.82E-05	8.48E-03
extracellular vesicle	2131	16	2.97	+	4.52E-05	9.03E-03
external encapsulating structure	560	8	5.65	+	7.49E-05	9.36E-03
extracellular matrix	559	8	5.66	+	7.40E-05	9.86E-03
extracellular space	3268	20	2.42	+	7.01E-05	1.00E-02
secretory granule	914	9	3.9	+	4.26E-04	5.00E-02
chylomicron	13	2	60.89	+	4.80E-04	5.05E-02
extracellular region	4305	22	2.02	+	4.66E-04	5.17E-02
cytoplasm	12372	43	1.38	+	5.87E-04	5.86E-02
low-density lipoprotein particle	15	2	52.77	+	6.44E-04	6.13E-02
very-low-density lipoprotein particle	21	2	37.69	+	1.28E-03	1.11E-01
triglyceride-rich plasma lipoprotein particle	21	2	37.69	+	1.28E-03	1.16E-01
secretory vesicle	1102	9	3.23	+	1.61E-03	1.34E-01
high-density lipoprotein particle	27	2	29.32	+	2.11E-03	1.69E-01
postsynaptic specialization membrane of symmetric synapse	1	1	> 100	+	2.53E-03	1.94E-01
plasma lipoprotein particle	37	2	21.39	+	3.94E-03	2.81E-01
lipoprotein particle	37	2	21.39	+	3.94E-03	2.92E-01
intracellular vesicle	2575	14	2.15	+	4.79E-03	2.99E-01

cytoplasmic vesicle	2567	14	2.16	+	4.70E-03	3.03E-01
vesicle	4041	19	1.86	+	4.41E-03	3.04E-01
postsynaptic specialization of symmetric synapse	2	1	> 100	+	5.05E-03	3.06E-01
protein-lipid complex	40	2	19.79	+	4.59E-03	3.06E-01
outer dynein arm docking complex	3	1	> 100	+	7.56E-03	4.44E-01
intracellular organelle	13488	43	1.26	+	8.20E-03	4.68E-01
growth cone	171	3	6.94	+	9.25E-03	5.13E-01
ciliary basal body	337	4	4.7	+	1.03E-02	5.28E-01
site of polarized growth	176	3	6.75	+	1.00E-02	5.40E-01
cytoskeleton	2545	13	2.02	+	1.03E-02	5.40E-01
intracellular anatomical structure	15119	46	1.2	+	1.16E-02	5.80E-01
endocytic vesicle	360	4	4.4	+	1.29E-02	6.28E-01
ripoptosome	6	1	65.96	+	1.51E-02	7.00E-01
CD95 death-inducing signaling complex	6	1	65.96	+	1.51E-02	7.17E-01
pre-snoRNP complex	7	1	56.54	+	1.76E-02	7.97E-01
mitochondrial envelope	836	6	2.84	+	1.83E-02	8.14E-01
spherical high-density lipoprotein particle	8	1	49.47	+	2.00E-02	8.52E-01
HFE-transferrin receptor complex	8	1	49.47	+	2.00E-02	8.70E-01
death-inducing signaling complex	9	1	43.97	+	2.25E-02	9.00E-01
azurophil granule lumen	90	2	8.79	+	2.18E-02	9.06E-01
postsynaptic endosome membrane	9	1	43.97	+	2.25E-02	9.18E-01
symmetric synapse	10	1	39.58	+	2.50E-02	9.60E-01
haptoglobin-hemoglobin complex	10	1	39.58	+	2.50E-02	9.79E-01
apical junction complex	155	0	< 0.01	-	1.00E+00	1.00E+00
apoptosome	3	0	< 0.01	-	1.00E+00	1.00E+00
cytoplasmic side of transport vesicle membrane	1	0	< 0.01	-	1.00E+00	1.00E+00
contractile muscle fiber	251	0	< 0.01	-	1.00E+00	1.00E+00
RAVE complex	3	0	< 0.01	-	1.00E+00	1.00E+00
deuterosome	6	0	< 0.01	-	1.00E+00	1.00E+00
dense core granule lumen	6	0	< 0.01	-	1.00E+00	1.00E+00
mitochondrial processing peptidase complex	2	0	< 0.01	-	1.00E+00	1.00E+00
postsynaptic early endosome membrane	1	0	< 0.01	-	1.00E+00	1.00E+00

ATPase dependent transmembrane transport complex	54	0	< 0.01	-	1.00E+00	1.00E+00
extrinsic component of presynaptic endocytic zone membrane	5	0	< 0.01	-	1.00E+00	1.00E+00
CatSper complex	14	0	< 0.01	-	1.00E+00	1.00E+00
extrinsic component of postsynaptic specialization membrane	5	0	< 0.01	-	1.00E+00	1.00E+00
sperm flagellum	232	1	1.71	+	4.46E-01	1.00E+00
CHOP-C/EBP complex	3	0	< 0.01	-	1.00E+00	1.00E+00
astrocyte projection	22	0	< 0.01	-	1.00E+00	1.00E+00
extrinsic component of postsynaptic membrane	13	0	< 0.01	-	1.00E+00	1.00E+00
fatty acid beta-oxidation multienzyme complex	3	0	< 0.01	-	1.00E+00	1.00E+00
dendritic tree	625	1	0.63	-	1.00E+00	1.00E+00
spine apparatus	4	0	< 0.01	-	1.00E+00	1.00E+00
sorting endosome	6	0	< 0.01	-	1.00E+00	1.00E+00
CA3 pyramidal cell dendrite	2	0	< 0.01	-	1.00E+00	1.00E+00
basal dendrite	6	0	< 0.01	-	1.00E+00	1.00E+00
apical dendrite	18	0	< 0.01	-	1.00E+00	1.00E+00
late recombination nodule	1	0	< 0.01	-	1.00E+00	1.00E+00
nuclear membrane protein complex	11	0	< 0.01	-	1.00E+00	1.00E+00
matrix side of mitochondrial inner membrane	7	0	< 0.01	-	1.00E+00	1.00E+00
recombination nodule	2	0	< 0.01	-	1.00E+00	1.00E+00
serine-type peptidase complex	21	0	< 0.01	-	1.00E+00	1.00E+00
chiasma	2	0	< 0.01	-	1.00E+00	1.00E+00
phosphatidylinositol 3-kinase complex, class III	7	0	< 0.01	-	1.00E+00	1.00E+00
gamma-catenin-TCF7L2 complex	1	0	< 0.01	-	1.00E+00	1.00E+00
catenin-TCF7L2 complex	4	0	< 0.01	-	1.00E+00	1.00E+00
U4/U6 x U5 tri-snRNP complex	35	0	< 0.01	-	1.00E+00	1.00E+00
extrinsic component of presynaptic membrane	9	0	< 0.01	-	1.00E+00	1.00E+00
RISC-loading complex	8	0	< 0.01	-	1.00E+00	1.00E+00
hyaluranon cable	2	0	< 0.01	-	1.00E+00	1.00E+00
trans-Golgi network transport vesicle membrane	21	0	< 0.01	-	1.00E+00	1.00E+00

intracellular cyclic nucleotide activated cation channel complex	6	0	< 0.01	-	1.00E+00	1.00E+00
somatodendritic compartment	848	3	1.4	+	4.75E-01	1.00E+00
platelet-derived growth factor receptor-ligand complex	2	0	< 0.01	-	1.00E+00	1.00E+00
Rpd3L-Expanded complex	2	0	< 0.01	-	1.00E+00	1.00E+00
dense body	5	0	< 0.01	-	1.00E+00	1.00E+00
spectrin	9	0	< 0.01	-	1.00E+00	1.00E+00
mitotic spindle pole	39	0	< 0.01	-	1.00E+00	1.00E+00
polytene chromosome	1	0	< 0.01	-	1.00E+00	1.00E+00
neurotransmitter receptor complex	57	0	< 0.01	-	1.00E+00	1.00E+00
ER to Golgi transport vesicle membrane	63	0	< 0.01	-	1.00E+00	1.00E+00
vesicle membrane	1284	3	0.92	-	1.00E+00	1.00E+00
endomembrane system	4901	17	1.37	+	1.42E-01	1.00E+00
epididymosome	1	0	< 0.01	-	1.00E+00	1.00E+00
platelet-derived growth factor complex	2	0	< 0.01	-	1.00E+00	1.00E+00
postsynaptic actin cytoskeleton	10	0	< 0.01	-	1.00E+00	1.00E+00
microbody lumen	51	0	< 0.01	-	1.00E+00	1.00E+00
late endosome lumen	9	0	< 0.01	-	1.00E+00	1.00E+00
early endosome lumen	3	0	< 0.01	-	1.00E+00	1.00E+00
microtubule bundle	6	0	< 0.01	-	1.00E+00	1.00E+00
endosome lumen	38	0	< 0.01	-	1.00E+00	1.00E+00
microbody membrane	65	0	< 0.01	-	1.00E+00	1.00E+00
cytoplasmic ribonucleoprotein granule	263	0	< 0.01	-	1.00E+00	1.00E+00
ribonuclease III complex	2	0	< 0.01	-	1.00E+00	1.00E+00
late endosome membrane	179	1	2.21	+	3.65E-01	1.00E+00
SUMO ligase complex	13	0	< 0.01	-	1.00E+00	1.00E+00
early endosome membrane	207	0	< 0.01	-	1.00E+00	1.00E+00
mitochondrial chromosome	2	0	< 0.01	-	1.00E+00	1.00E+00
tubular endosome	5	0	< 0.01	-	1.00E+00	1.00E+00
**GO Molecular Function**						
phosphatidylcholine-sterol O-acyltransferase activator activity	6	2	> 100	+	9.33E-05	4.73E-01
macrolide binding	12	2	65.96	+	4.07E-04	6.87E-01

ferric iron binding	10	2	79.15	+	2.78E-04	7.05E-01
lipid transfer activity	66	3	17.99	+	6.22E-04	7.89E-01
butanol dehydrogenase (NAD+) activity	1	1	> 100	+	2.53E-03	8.01E-01
peptidase regulator activity	236	4	6.71	+	2.97E-03	8.35E-01
thiosulfate transmembrane transporter activity	1	1	> 100	+	2.53E-03	8.54E-01
peptidase inhibitor activity	177	4	8.94	+	1.04E-03	8.77E-01
sterol transfer activity	32	2	24.74	+	2.96E-03	8.83E-01
endopeptidase inhibitor activity	169	4	9.37	+	8.74E-04	8.86E-01
antioxidant activity	84	3	14.13	+	1.25E-03	9.09E-01
C5a anaphylatoxin chemotactic receptor binding	1	1	> 100	+	2.53E-03	9.15E-01
cholesterol transfer activity	25	2	31.66	+	1.81E-03	9.18E-01
oxaloacetate transmembrane transporter activity	2	1	> 100	+	5.05E-03	9.84E-01
C5L2 anaphylatoxin chemotactic receptor binding	1	1	> 100	+	2.53E-03	9.86E-01
endopeptidase regulator activity	198	4	8	+	1.57E-03	9.94E-01
growth hormone receptor binding	10	0	< 0.01	-	1.00E+00	1.00E+00
granulocyte colony-stimulating factor receptor binding	1	0	< 0.01	-	1.00E+00	1.00E+00
14-3-3 protein binding	32	0	< 0.01	-	1.00E+00	1.00E+00
neuromedin U binding	2	0	< 0.01	-	1.00E+00	1.00E+00
neuropeptide binding	33	0	< 0.01	-	1.00E+00	1.00E+00
neuromedin U receptor binding	1	0	< 0.01	-	1.00E+00	1.00E+00
1-(4-iodo-2,5-dimethoxyphenyl)propan-2-amine binding	2	0	< 0.01	-	1.00E+00	1.00E+00
N-terminal protein N-methyltransferase activity	2	0	< 0.01	-	1.00E+00	1.00E+00
granulocyte macrophage colony-stimulating factor receptor binding	1	0	< 0.01	-	1.00E+00	1.00E+00
erythropoietin receptor binding	3	0	< 0.01	-	1.00E+00	1.00E+00
ciliary neurotrophic factor receptor binding	6	0	< 0.01	-	1.00E+00	1.00E+00
cytokine receptor binding	274	2	2.89	+	1.52E-01	1.00E+00
cytokine activity	237	1	1.67	+	4.53E-01	1.00E+00

scavenger receptor binding	4	0	< 0.01	-	1.00E+00	1.00E+00
death receptor binding	21	1	18.85	+	5.18E-02	1.00E+00
Toll binding	1	0	< 0.01	-	1.00E+00	1.00E+00
lactosylceramide 4-alpha-galactosyltransferase activity	1	0	< 0.01	-	1.00E+00	1.00E+00
N-acetylgalactosaminyl-proteoglycan 3-beta-glucuronosyltransferase activity	4	0	< 0.01	-	1.00E+00	1.00E+00
xenobiotic transmembrane transporter activity	32	0	< 0.01	-	1.00E+00	1.00E+00
inositol trisphosphate phosphatase activity	14	0	< 0.01	-	1.00E+00	1.00E+00
smoothened binding	4	0	< 0.01	-	1.00E+00	1.00E+00
Tat protein binding	10	0	< 0.01	-	1.00E+00	1.00E+00
potassium ion binding	13	0	< 0.01	-	1.00E+00	1.00E+00
receptor tyrosine kinase-like orphan receptor binding	1	0	< 0.01	-	1.00E+00	1.00E+00
type II transforming growth factor beta receptor binding	11	0	< 0.01	-	1.00E+00	1.00E+00
N-acetylglucosaminyl-proteoglycan 4-beta-glucuronosyltransferase activity	2	0	< 0.01	-	1.00E+00	1.00E+00
patched binding	8	0	< 0.01	-	1.00E+00	1.00E+00
glucuronosyl-N-acetylglucosaminyl-proteoglycan 4-alpha-N-acetylglucosaminyltransferase activity	3	0	< 0.01	-	1.00E+00	1.00E+00
Notch binding	26	0	< 0.01	-	1.00E+00	1.00E+00
type 2 fibroblast growth factor receptor binding	5	0	< 0.01	-	1.00E+00	1.00E+00
hyaluronan synthase activity	3	0	< 0.01	-	1.00E+00	1.00E+00
tubulin deacetylase activity	2	0	< 0.01	-	1.00E+00	1.00E+00
phospholipid:diacylglycerol acyltransferase activity	1	0	< 0.01	-	1.00E+00	1.00E+00
frizzled binding	40	0	< 0.01	-	1.00E+00	1.00E+00
type 1 fibroblast growth factor receptor binding	6	0	< 0.01	-	1.00E+00	1.00E+00
mitochondrion targeting sequence binding	5	0	< 0.01	-	1.00E+00	1.00E+00
fibroblast growth factor receptor binding	27	0	< 0.01	-	1.00E+00	1.00E+00
endoplasmic reticulum signal peptide binding	3	0	< 0.01	-	1.00E+00	1.00E+00

signaling receptor binding	1544	9	2.31	+	1.44E-02	1.00E+00
glycerol-3-phosphate dehydrogenase (NAD+) activity	2	0	< 0.01	-	1.00E+00	1.00E+00
5'-3' RNA polymerase activity	33	0	< 0.01	-	1.00E+00	1.00E+00
DNA polymerase activity	40	0	< 0.01	-	1.00E+00	1.00E+00
neuropeptide receptor binding	41	0	< 0.01	-	1.00E+00	1.00E+00
oxidoreductase activity, acting on peroxide as acceptor	58	0	< 0.01	-	1.00E+00	1.00E+00
enoyl-[acyl-carrier-protein] reductase (NADPH) activity	2	0	< 0.01	-	1.00E+00	1.00E+00
intracellularly calcium-gated channel activity	19	0	< 0.01	-	1.00E+00	1.00E+00
RNA strand-exchange activity	2	0	< 0.01	-	1.00E+00	1.00E+00
estrogen response element binding	11	0	< 0.01	-	1.00E+00	1.00E+00
benzoate-CoA ligase activity	3	0	< 0.01	-	1.00E+00	1.00E+00
oxidoreductase activity, acting on diphenols and related substances as donors	9	0	< 0.01	-	1.00E+00	1.00E+00
oxidoreductase activity, acting on a heme group of donors	14	0	< 0.01	-	1.00E+00	1.00E+00
oxidoreductase activity, acting on a sulfur group of donors, quinone or similar compound as acceptor	3	0	< 0.01	-	1.00E+00	1.00E+00
oxidoreductase activity, acting on a sulfur group of donors, disulfide as acceptor	8	0	< 0.01	-	1.00E+00	1.00E+00
oxidoreductase activity, acting on a sulfur group of donors, oxygen as acceptor	11	0	< 0.01	-	1.00E+00	1.00E+00
XTP diphosphatase activity	1	0	< 0.01	-	1.00E+00	1.00E+00
UTP diphosphatase activity	3	0	< 0.01	-	1.00E+00	1.00E+00
ITP diphosphatase activity	1	0	< 0.01	-	1.00E+00	1.00E+00
poly(G) binding	8	0	< 0.01	-	1.00E+00	1.00E+00
diphthine methyl ester synthase activity	1	0	< 0.01	-	1.00E+00	1.00E+00
DNA N6-methyladenine demethylase activity	2	0	< 0.01	-	1.00E+00	1.00E+00
ABC-type sterol transporter activity	2	0	< 0.01	-	1.00E+00	1.00E+00

ATPase-coupled lipid transmembrane transporter activity	9	0	< 0.01	-	1.00E+00	1.00E+00
HMG box domain binding	12	0	< 0.01	-	1.00E+00	1.00E+00
oxidoreductase activity, acting on a sulfur group of donors, NAD(P) as acceptor	11	0	< 0.01	-	1.00E+00	1.00E+00
oxidoreductase activity, acting on a sulfur group of donors	55	0	< 0.01	-	1.00E+00	1.00E+00
oxidoreductase activity, acting on other nitrogenous compounds as donors, cytochrome as acceptor	4	0	< 0.01	-	1.00E+00	1.00E+00
GTP diphosphatase activity	2	0	< 0.01	-	1.00E+00	1.00E+00
oxidoreductase activity, acting on other nitrogenous compounds as donors	12	0	< 0.01	-	1.00E+00	1.00E+00
dTTP diphosphatase activity	1	0	< 0.01	-	1.00E+00	1.00E+00
8-oxo-7,8-dihydroguanine DNA N-glycosylase activity	2	0	< 0.01	-	1.00E+00	1.00E+00
short-chain fatty acyl-CoA hydrolase activity	1	0	< 0.01	-	1.00E+00	1.00E+00
deoxyhypusine synthase activity	1	0	< 0.01	-	1.00E+00	1.00E+00
tRNA-queuosine(34) galactosyltransferase activity	1	0	< 0.01	-	1.00E+00	1.00E+00
TPR domain binding	8	0	< 0.01	-	1.00E+00	1.00E+00
N-box binding	1	0	< 0.01	-	1.00E+00	1.00E+00
oxidoreductase activity, acting on NAD(P)H, quinone or similar compound as acceptor	58	0	< 0.01	-	1.00E+00	1.00E+00
oxidoreductase activity, acting on NAD(P)H, heme protein as acceptor	11	0	< 0.01	-	1.00E+00	1.00E+00
oxidoreductase activity, acting on NAD(P)H as acceptor	1	0	< 0.01	-	1.00E+00	1.00E+00
oxidoreductase activity, acting on NAD(P)H	90	0	< 0.01	-	1.00E+00	1.00E+00
nitric oxide dioxygenase activity, heme protein as donor	1	0	< 0.01	-	1.00E+00	1.00E+00
diacylglycerol-dependent, calcium-independent	4	0	< 0.01	-	1.00E+00	1.00E+00

serine/threonine kinase activity						
calcium,diacylglycerol-dependent serine/threonine kinase activity	3	0	< 0.01	-	1.00E+00	1.00E+00
transporter inhibitor activity	52	0	< 0.01	-	1.00E+00	1.00E+00
diacylglycerol-dependent serine/threonine kinase activity	17	0	< 0.01	-	1.00E+00	1.00E+00
eukaryotic translation initiation factor 2alpha kinase activity	4	0	< 0.01	-	1.00E+00	1.00E+00
cyclin-dependent protein serine/threonine kinase activity	29	1	13.65	+	7.08E-02	1.00E+00
cGMP-dependent protein kinase activity	2	0	< 0.01	-	1.00E+00	1.00E+00
cAMP-dependent protein kinase activity	7	0	< 0.01	-	1.00E+00	1.00E+00
cyclic nucleotide-dependent protein kinase activity	9	0	< 0.01	-	1.00E+00	1.00E+00
protein-lipid complex binding	30	1	13.19	+	7.31E-02	1.00E+00
Delta4-3-oxosteroid 5beta-reductase activity	2	0	< 0.01	-	1.00E+00	1.00E+00
lipoprotein particle binding	30	1	13.19	+	7.31E-02	1.00E+00
oxidoreductase activity, acting on the CH-NH group of donors, quinone or similar compound as acceptor	4	0	< 0.01	-	1.00E+00	1.00E+00
oxidoreductase activity, acting on the CH-NH group of donors, oxygen as acceptor	3	0	< 0.01	-	1.00E+00	1.00E+00
cyanamide hydratase activity	2	0	< 0.01	-	1.00E+00	1.00E+00
oxidoreductase activity, acting on the CH-NH group of donors, NAD or NADP as acceptor	17	0	< 0.01	-	1.00E+00	1.00E+00
corticosterone 18-monooxygenase activity	2	0	< 0.01	-	1.00E+00	1.00E+00
oxidoreductase activity, acting on the CH-NH group of donors	26	0	< 0.01	-	1.00E+00	1.00E+00
oxidoreductase activity, acting on the CH-NH2 group of donors, disulfide as acceptor	1	0	< 0.01	-	1.00E+00	1.00E+00

oxidoreductase activity, acting on the CH-NH2 group of donors, oxygen as acceptor	16	0	< 0.01	-	1.00E+00	1.00E+00
transporter activator activity	26	0	< 0.01	-	1.00E+00	1.00E+00
transporter regulator activity	191	0	< 0.01	-	1.00E+00	1.00E+00

Table S4 Experimental conditions for the validation of the selected proteins signature peptides

**Accession Number**	**Protein Name**	**Peptide Sequences**	**Length (AA)**	**Molecular weight (g/mol)**	**MRM Transition**		**Cone Voltage (v)**	**Collision Energy (v)**	**RT (min)**
					MS1 (m/z)(z)	MS2(m/z) (ion type)			
Q96CT7	Coiled-coil domain-containing protein 124	R.AAFTAFEEAQLPR.L[171, 183]	13	1535.66	484.2490	175.1190 [y1]	20	20	5.06
P02818	Osteocalcin	EVCELNPDCDELADHIGFQEAY [72,93]	23	2666.85	837.9	182.12 [y1]	34	25	5.84
P02647	Apolipoprotein A1	EQLGPVTQEFWDNLEK[86,101]	16	1933.08	644.9828	951.4571 [y7]	20	20	5.05
						1080.5 [y2]			
P00738	Haptoglobin	R.TEGDGVYTLNNEK.Q [119, 131]	13	1458.58	480.5598	390.1983 [y3]	20	20	4.47
						276[y2]			
