# Supplementary figures and images for: Proteomic Profiling Reveals Novel Molecular Insights into Dysregulated Proteins in Established Cases of Rheumatoid Arthritis
Source: Proteomes. 2025 Jul 4;13(3):32. doi: 10.3390/proteomes13030032 (PMC12286002; doi:10.3390/proteomes13030032)

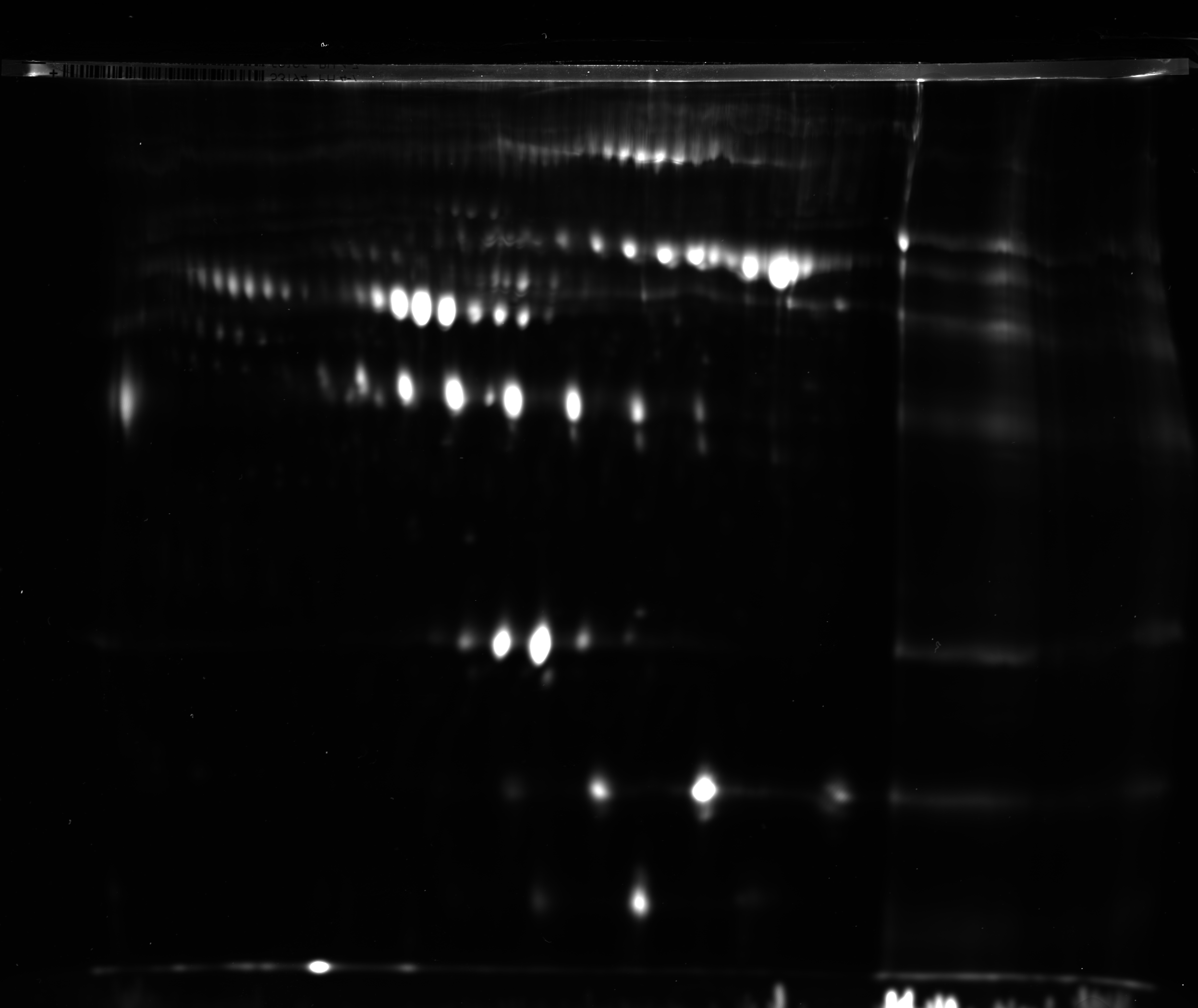

Supplement: Supplementary file 1 [file proteomes-13-00032-s001.zip › GELS/GEL1-Cy3_PUB_254.bmp]

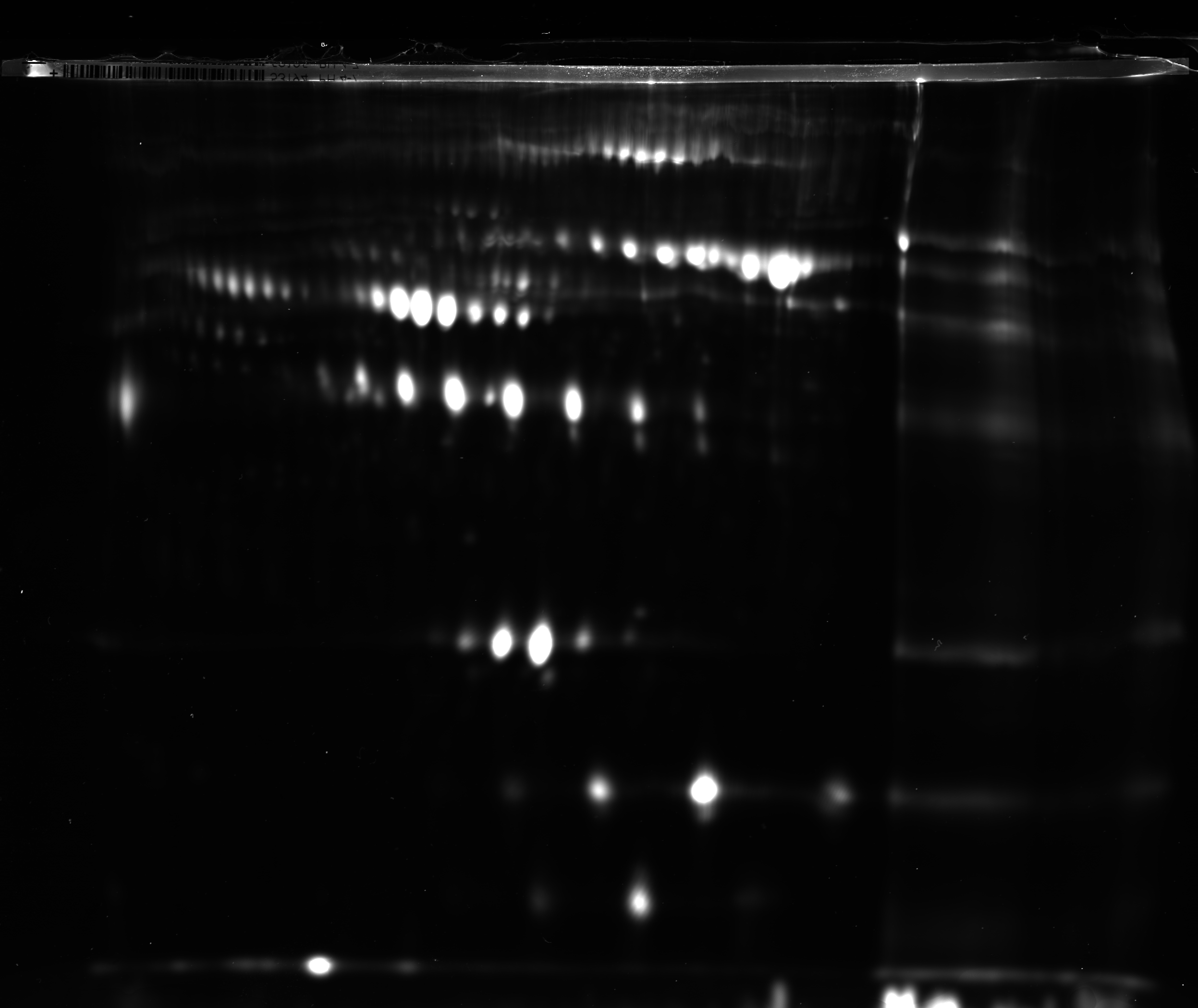

Supplement: Supplementary file 1 [file proteomes-13-00032-s001.zip › GELS/GEL1-Cy5_PUB_254 (2).bmp]

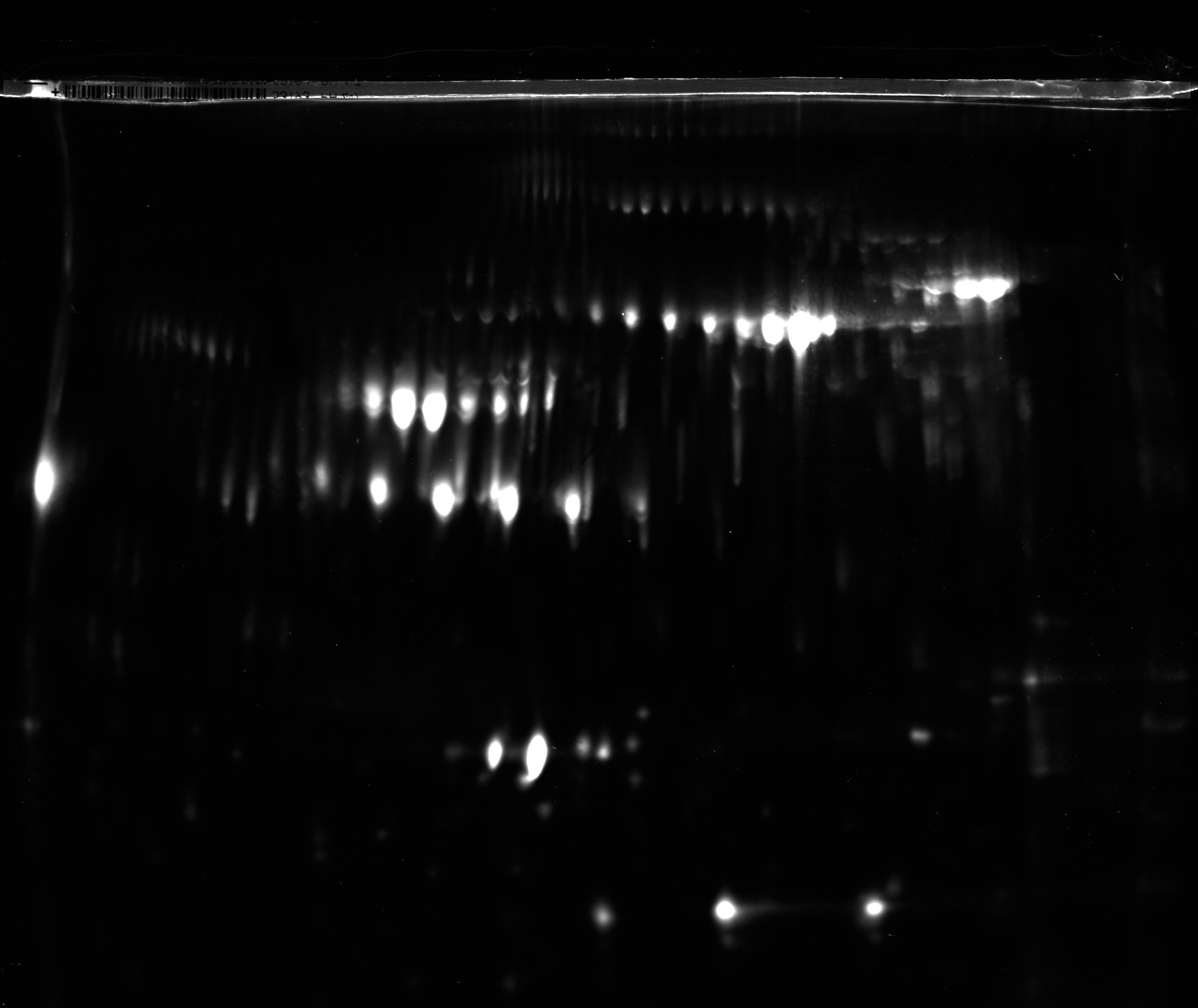

Supplement: Supplementary file 1 [file proteomes-13-00032-s001.zip › GELS/GEL10-CY3_PUB_254.bmp]

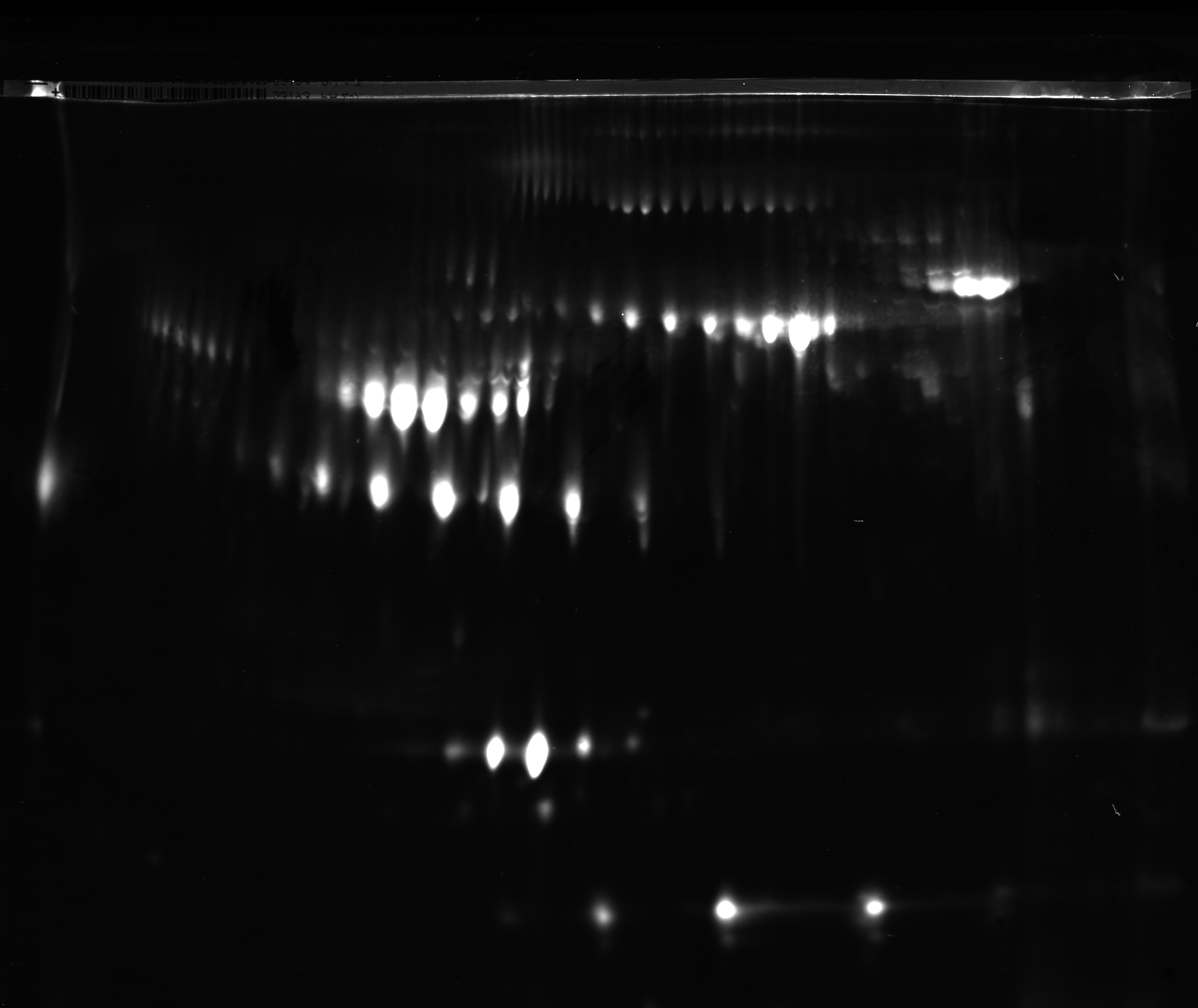

Supplement: Supplementary file 1 [file proteomes-13-00032-s001.zip › GELS/GEL10-CY5_PUB_254.bmp]

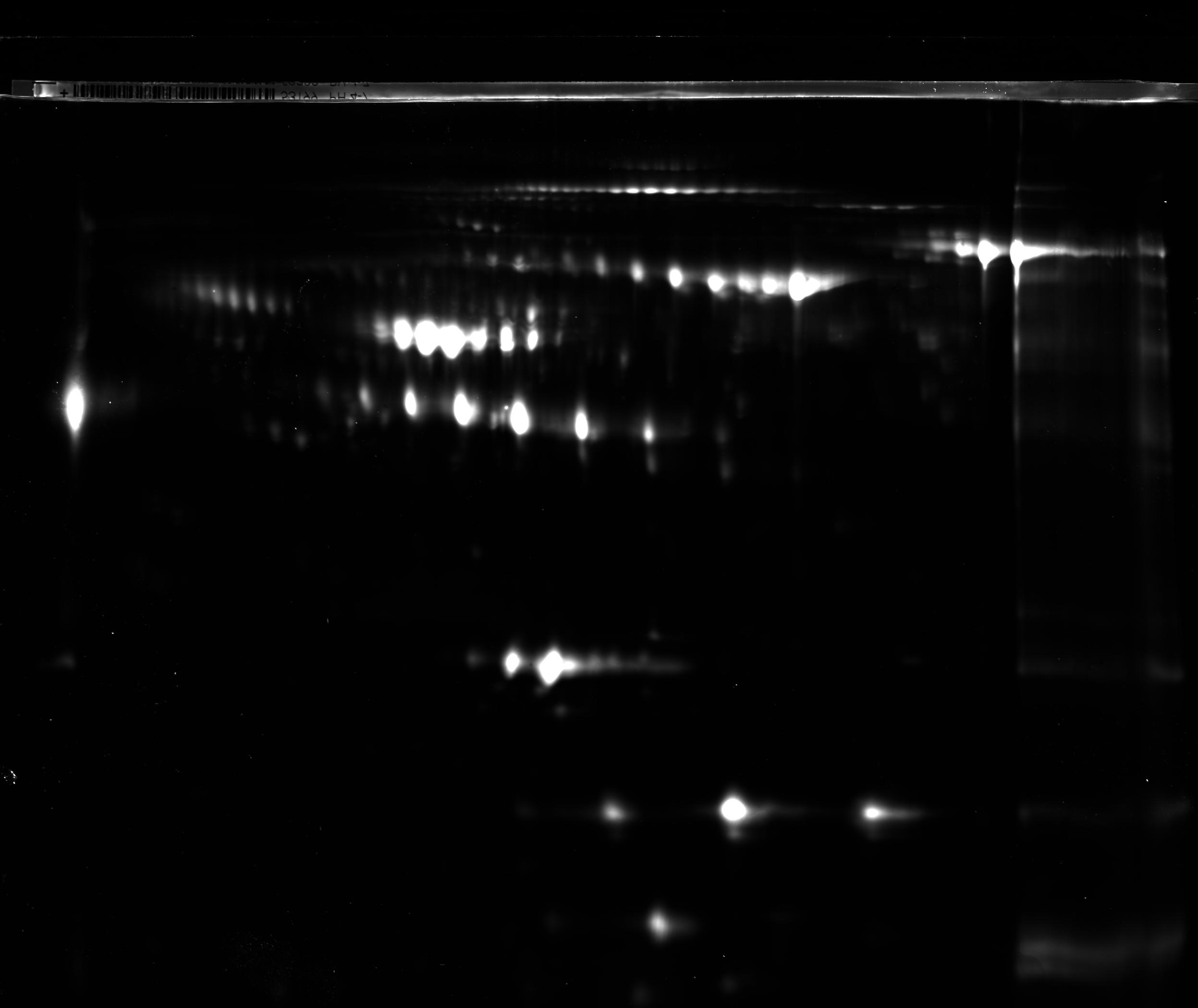

Supplement: Supplementary file 1 [file proteomes-13-00032-s001.zip › GELS/GEL11-CY3_PUB_254.bmp]

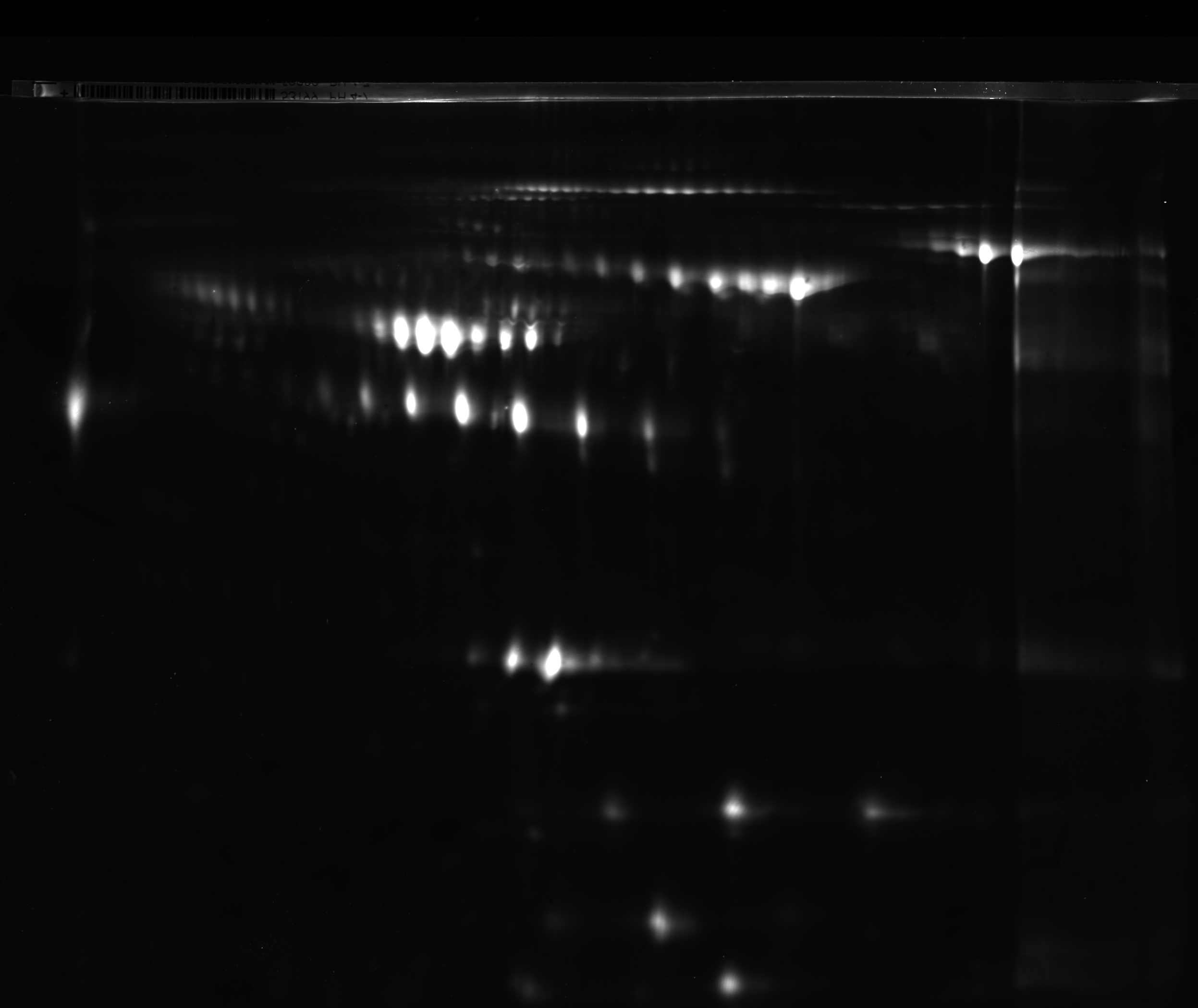

Supplement: Supplementary file 1 [file proteomes-13-00032-s001.zip › GELS/GEL11-CY5_PUB_254.bmp]

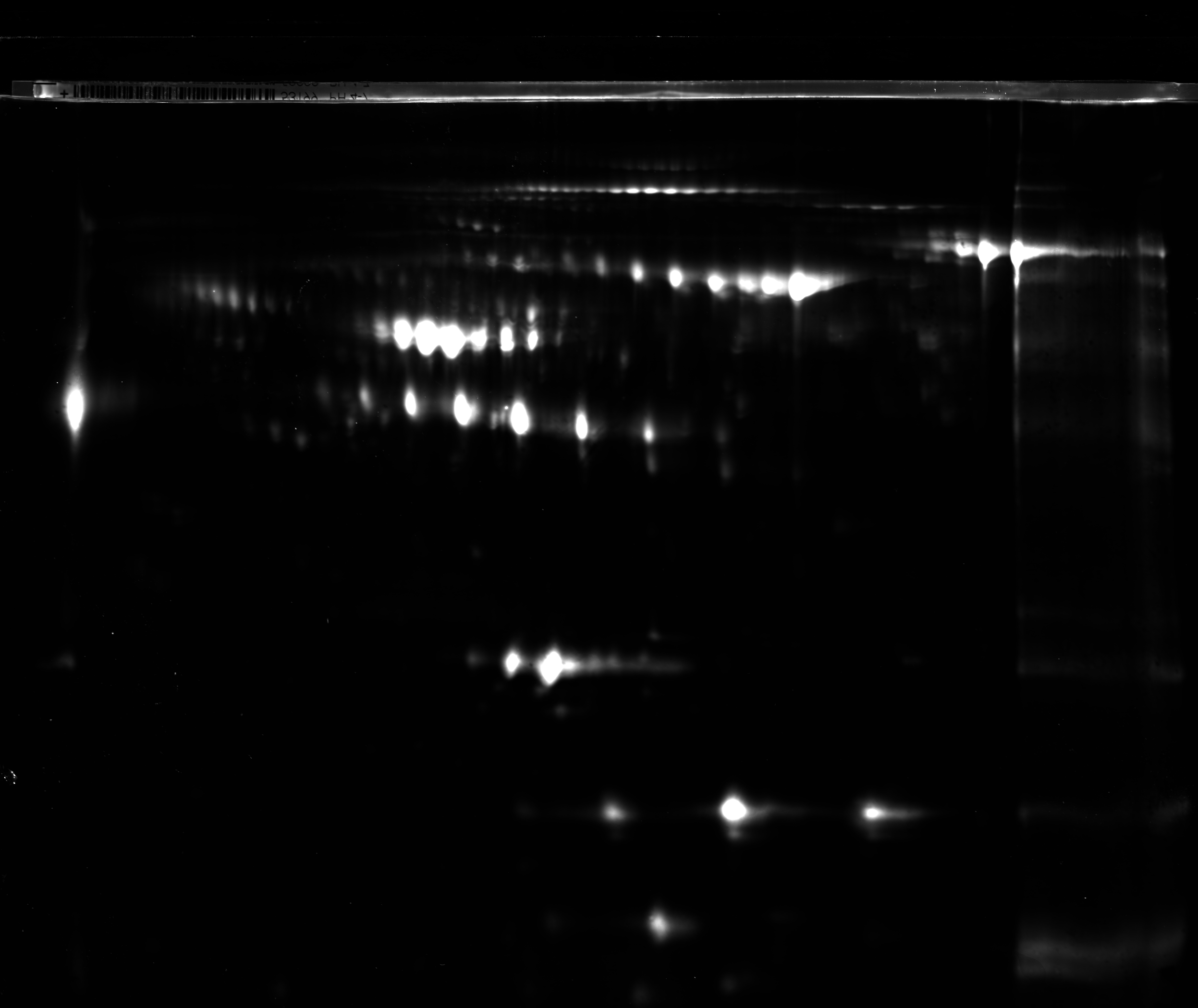

Supplement: Supplementary file 1 [file proteomes-13-00032-s001.zip › GELS/GEL12-CY3_PUB_254.bmp]

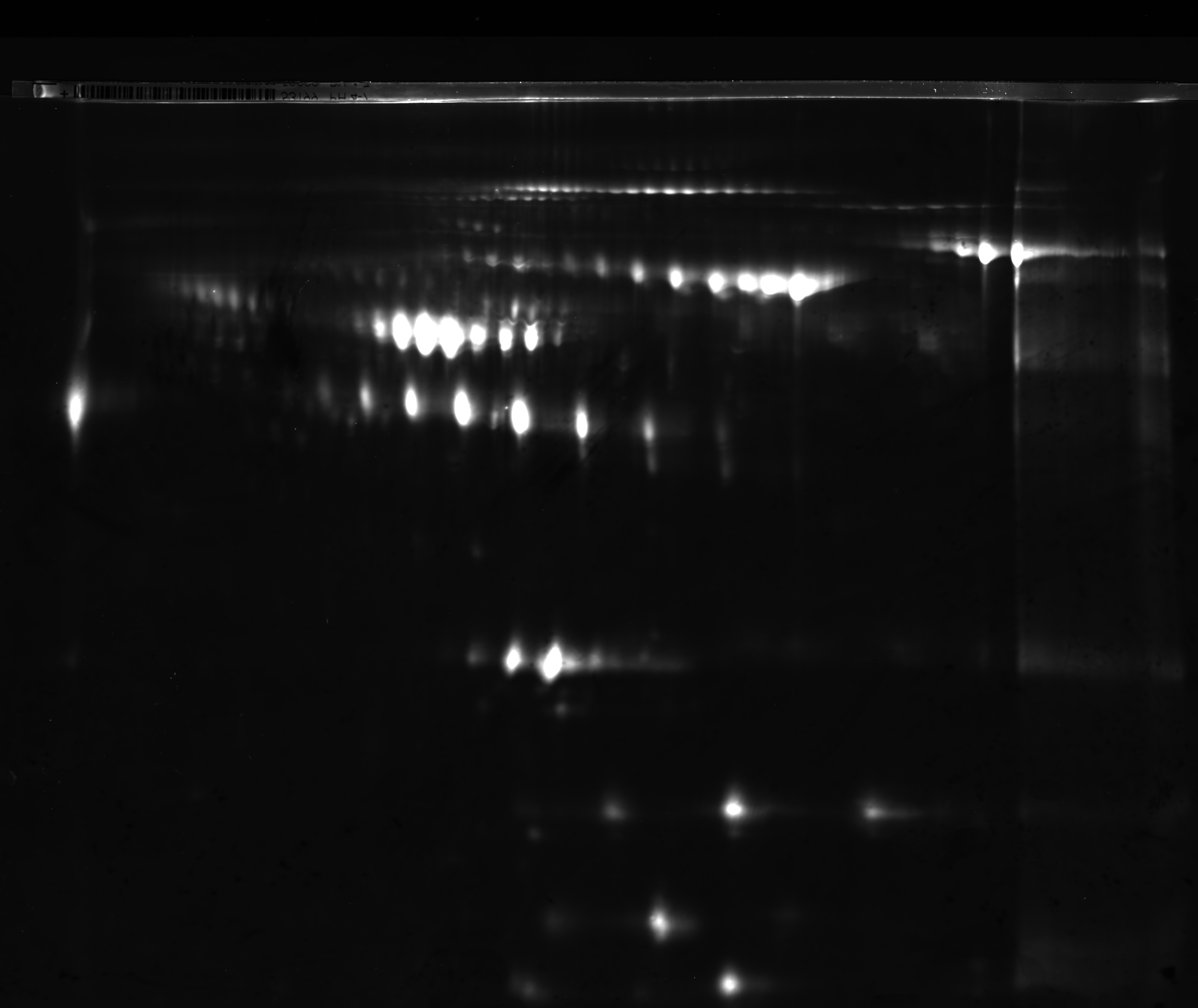

Supplement: Supplementary file 1 [file proteomes-13-00032-s001.zip › GELS/GEL12-CY5_PUB_254.bmp]

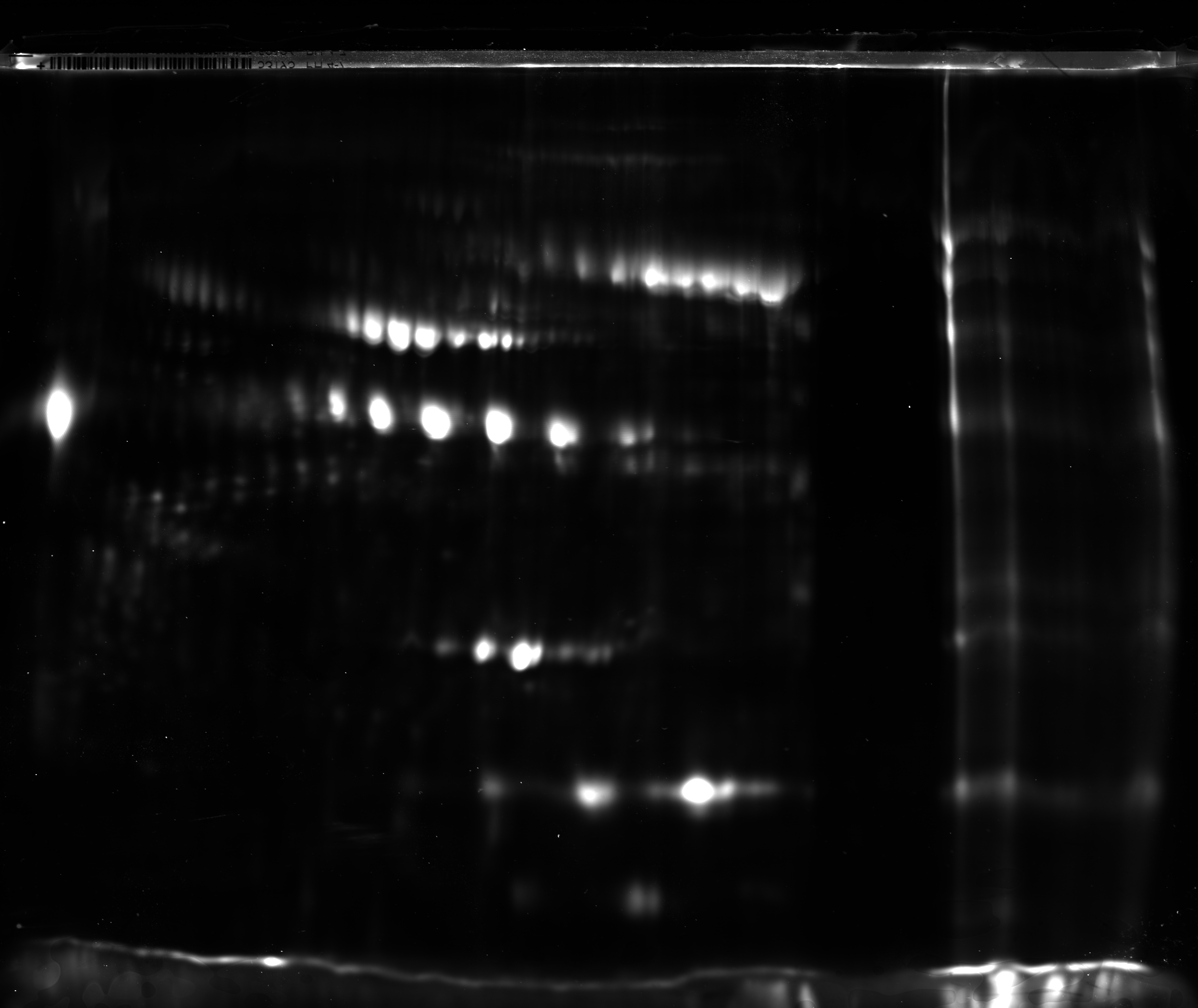

Supplement: Supplementary file 1 [file proteomes-13-00032-s001.zip › GELS/GEL2-Cy3_PUB_254.bmp]

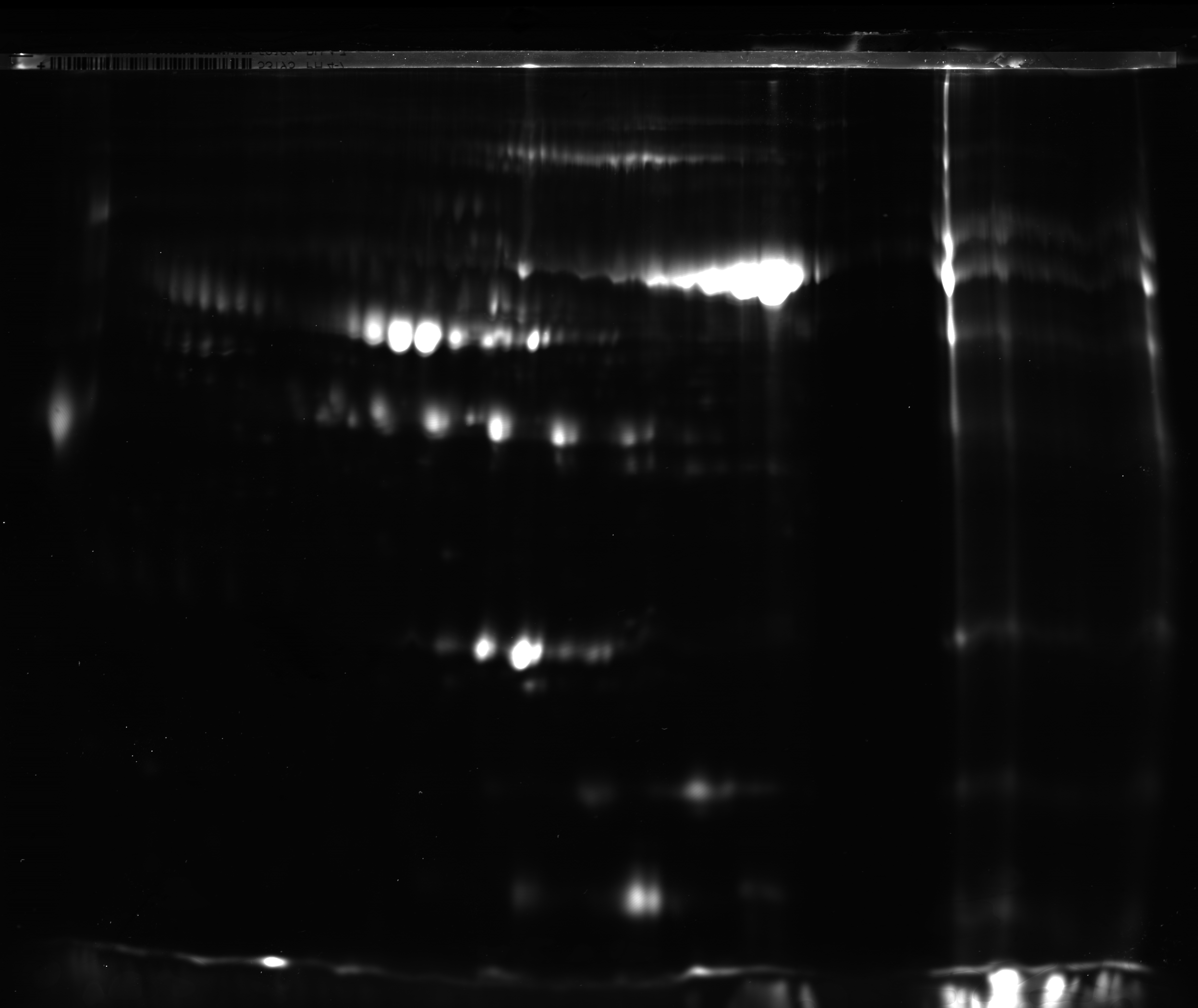

Supplement: Supplementary file 1 [file proteomes-13-00032-s001.zip › GELS/GEL2-Cy5_PUB_254.bmp]

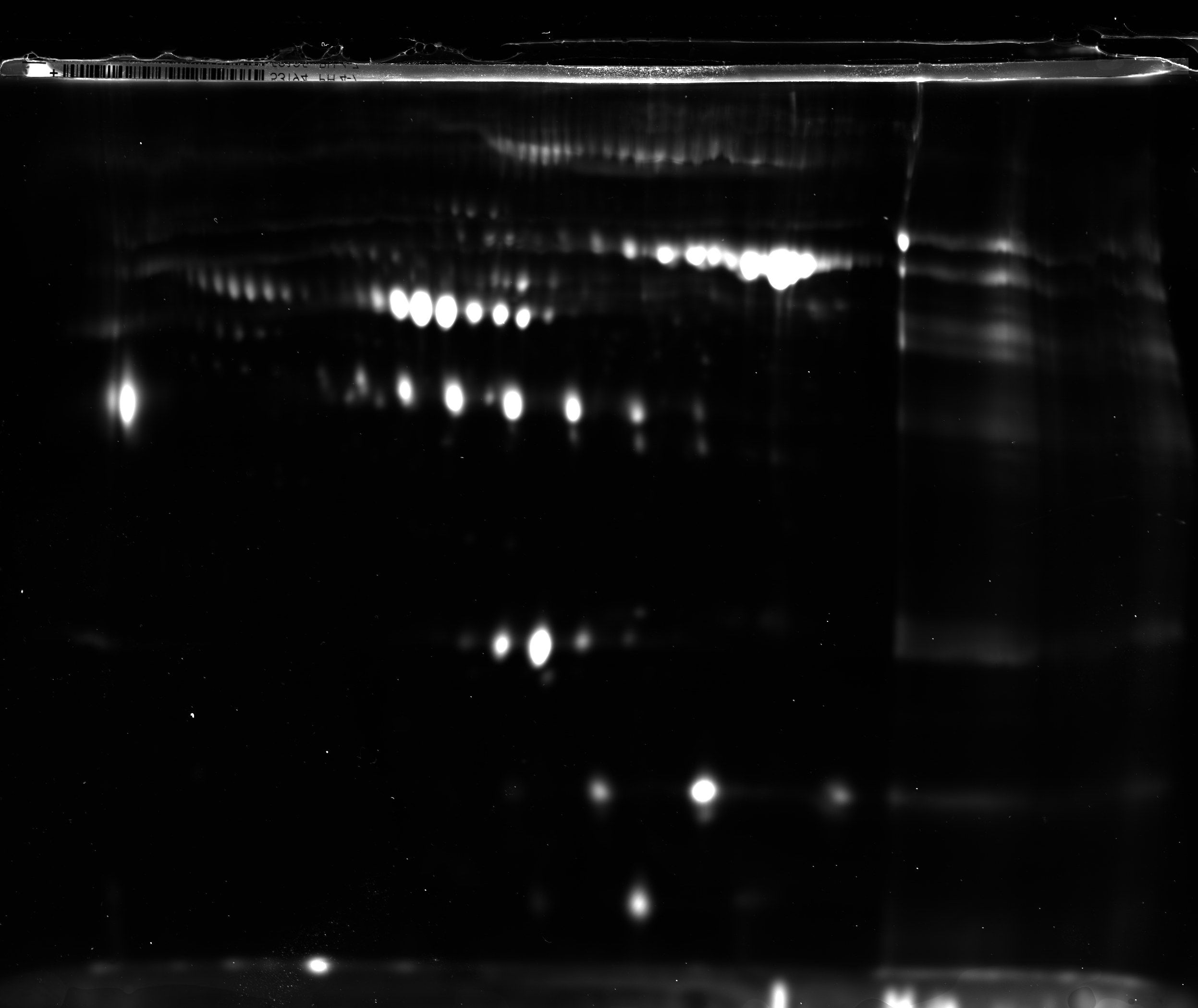

Supplement: Supplementary file 1 [file proteomes-13-00032-s001.zip › GELS/GEL3-Cy3_PUB_25.bmp]

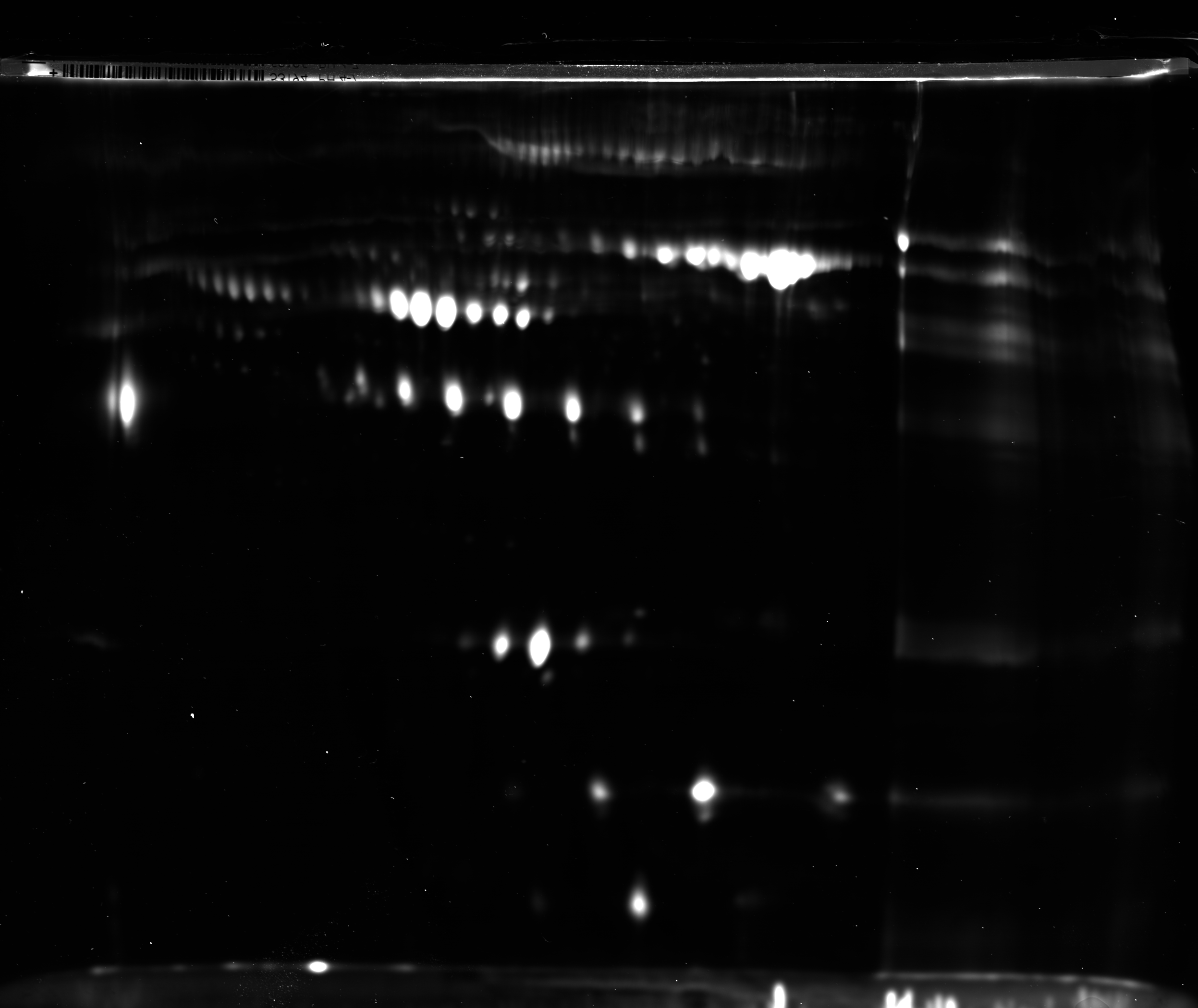

Supplement: Supplementary file 1 [file proteomes-13-00032-s001.zip › GELS/GEL3-cy5_PUB_25.bmp]

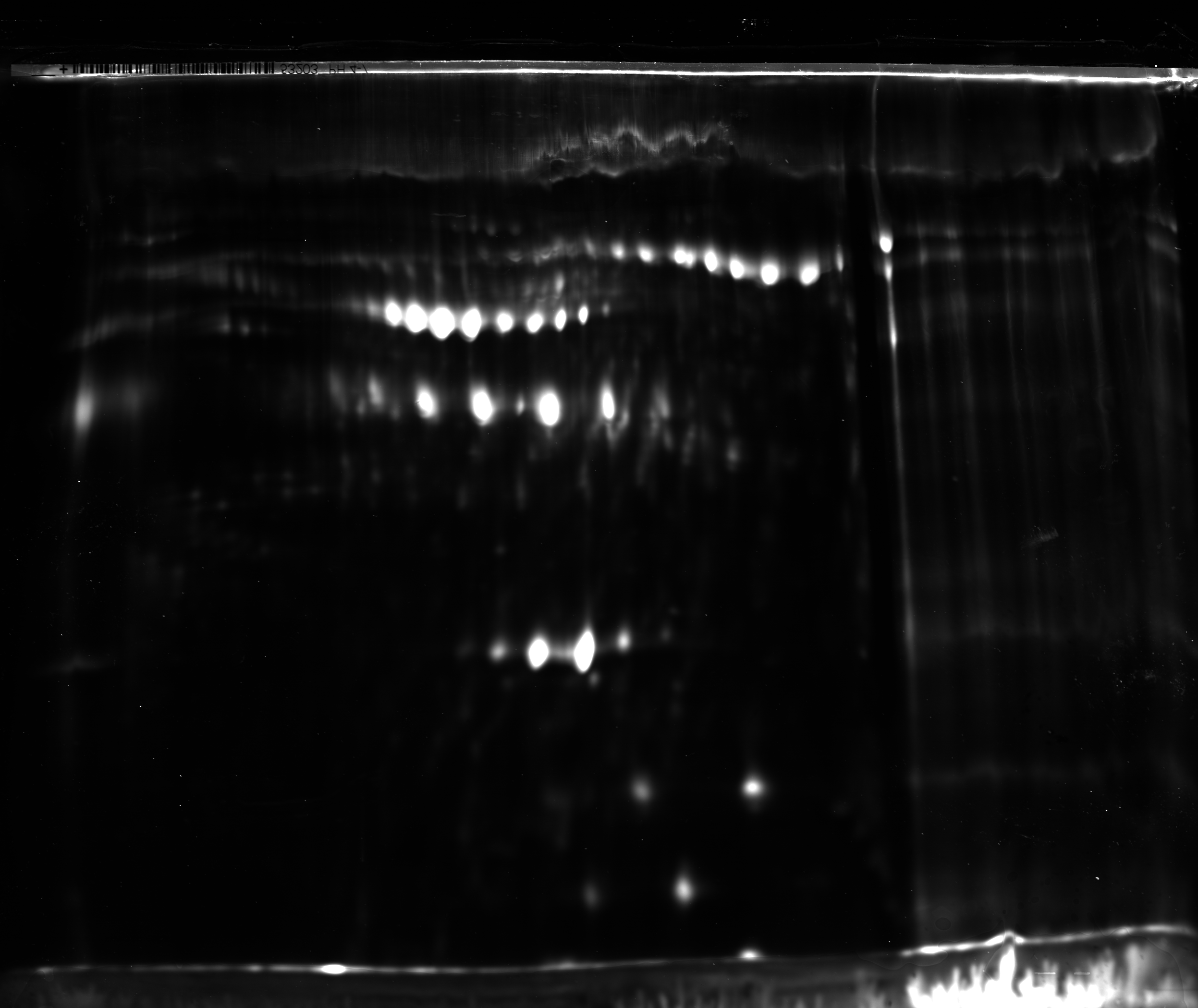

Supplement: Supplementary file 1 [file proteomes-13-00032-s001.zip › GELS/gel4-Cy3_PUB_254.bmp]

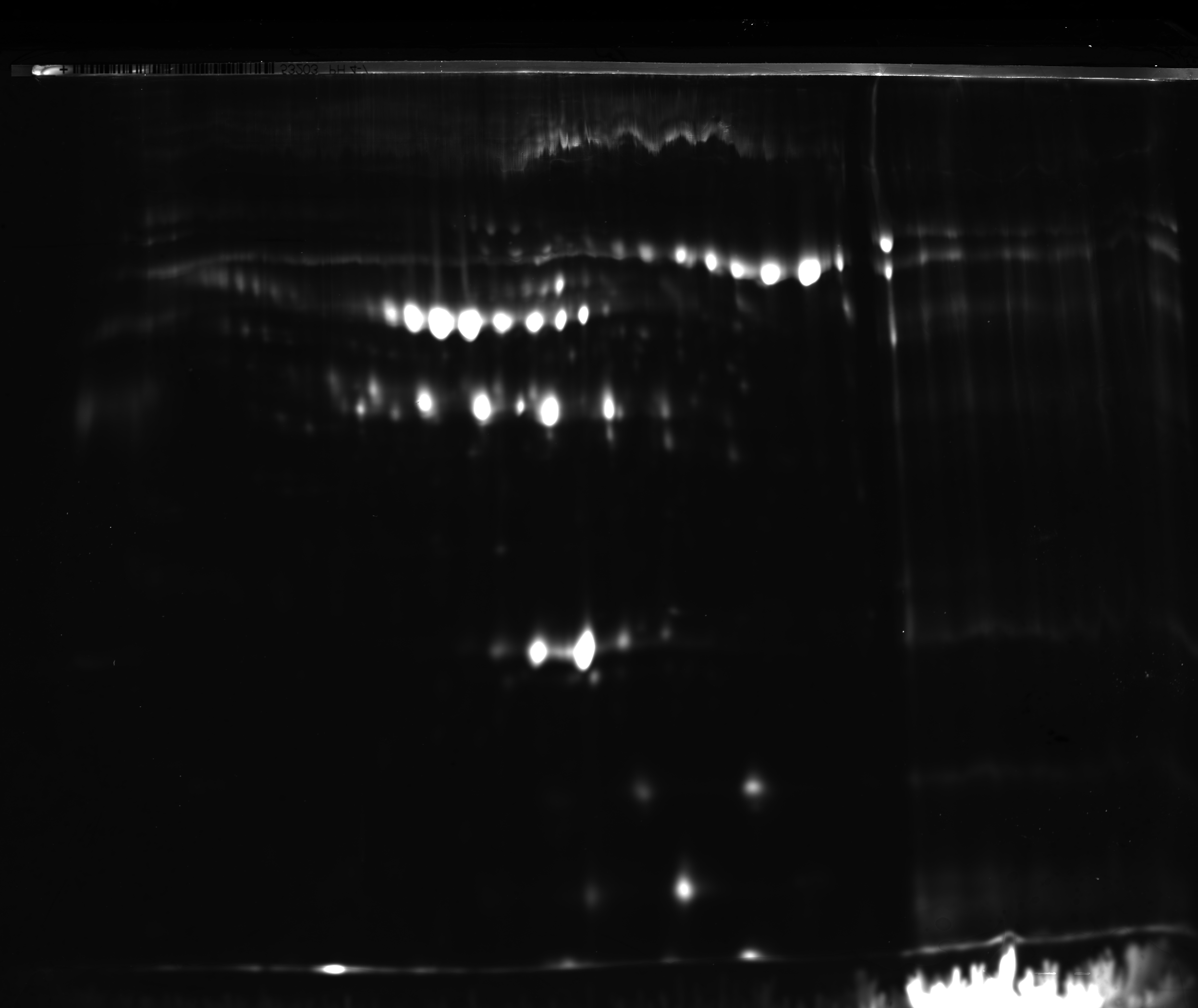

Supplement: Supplementary file 1 [file proteomes-13-00032-s001.zip › GELS/gel4-Cy5_PUB_254.bmp]

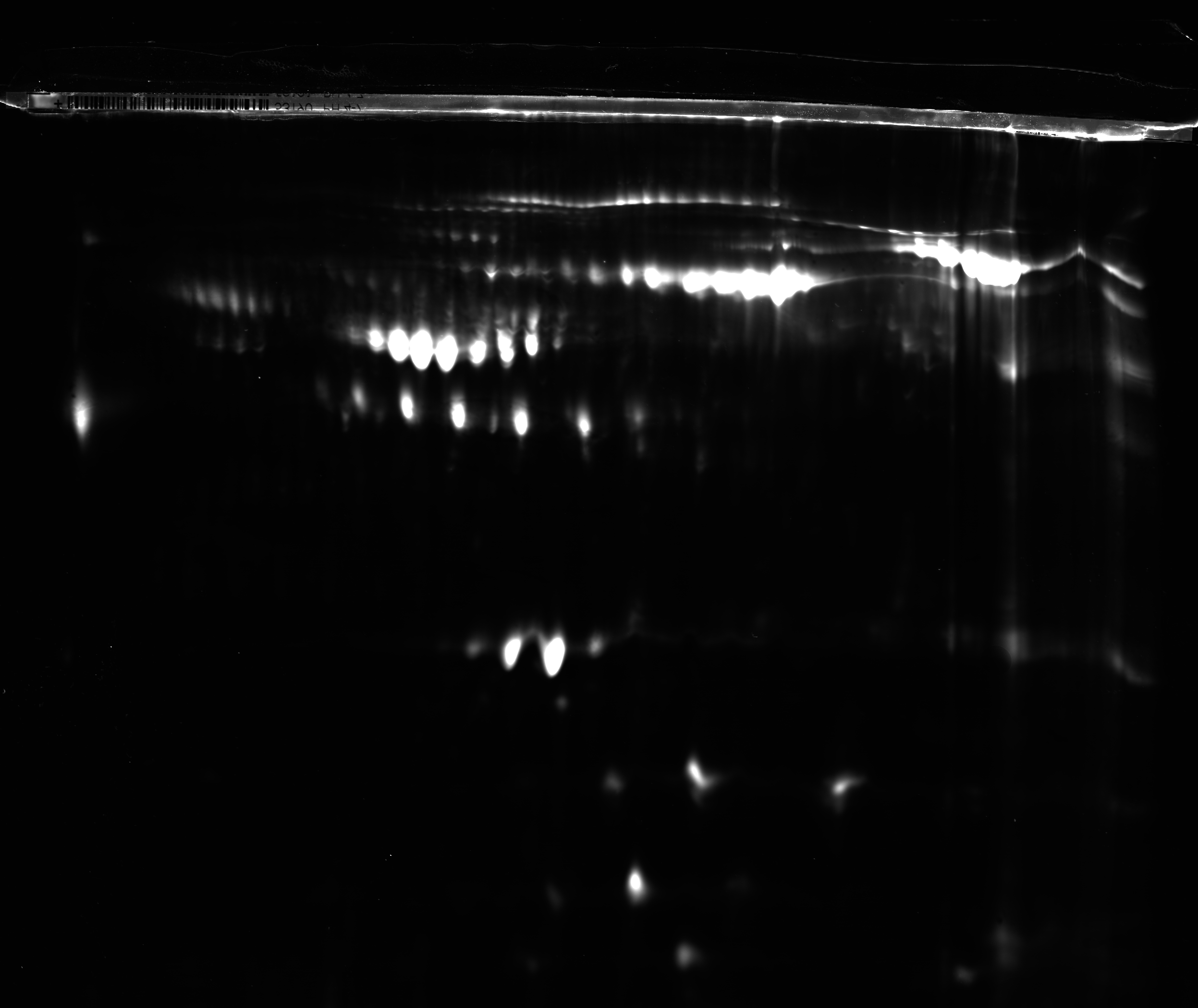

Supplement: Supplementary file 1 [file proteomes-13-00032-s001.zip › GELS/GEL5-CY3_PUB_254.bmp]

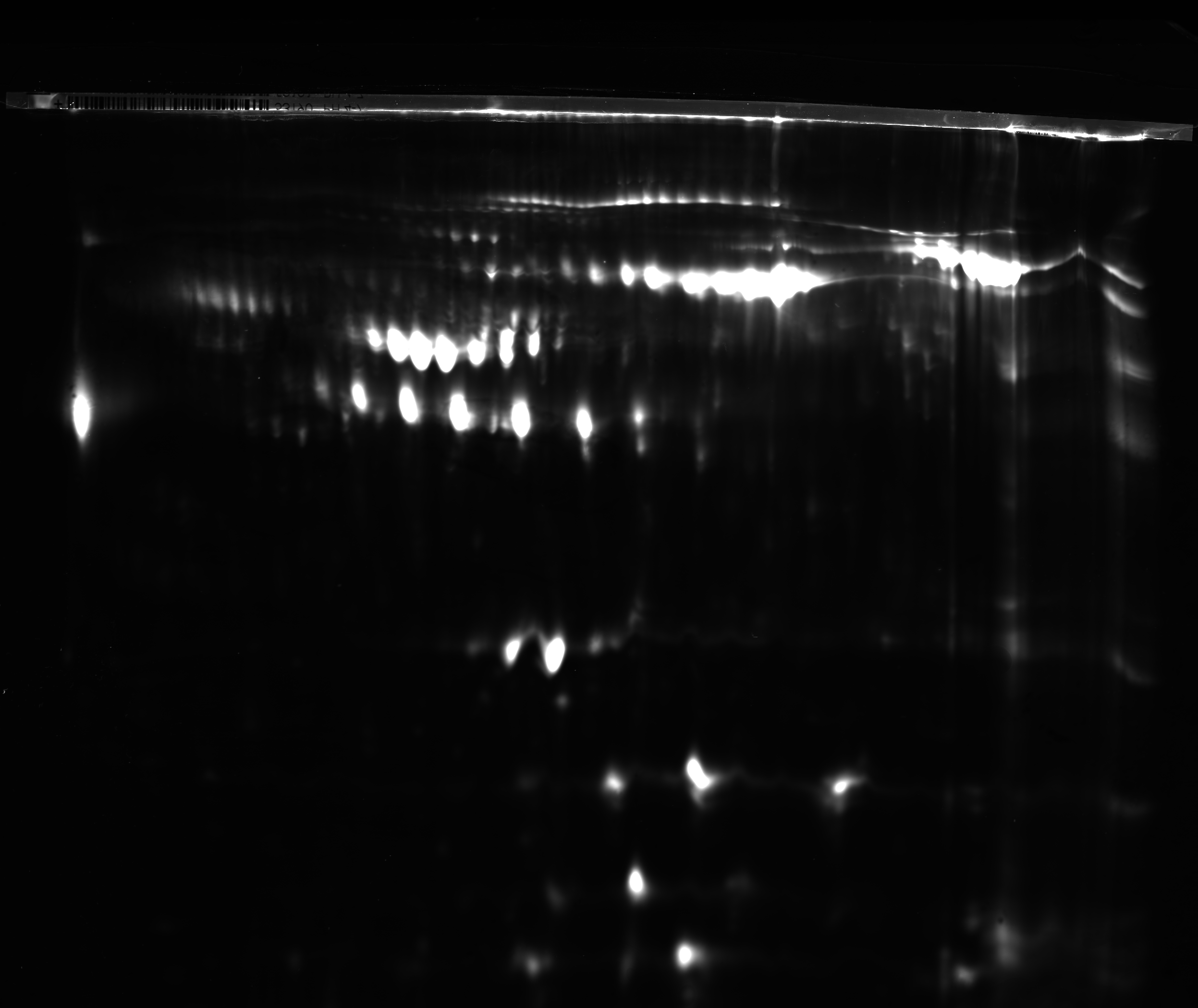

Supplement: Supplementary file 1 [file proteomes-13-00032-s001.zip › GELS/GEL5-CY5_PUB_254.bmp]

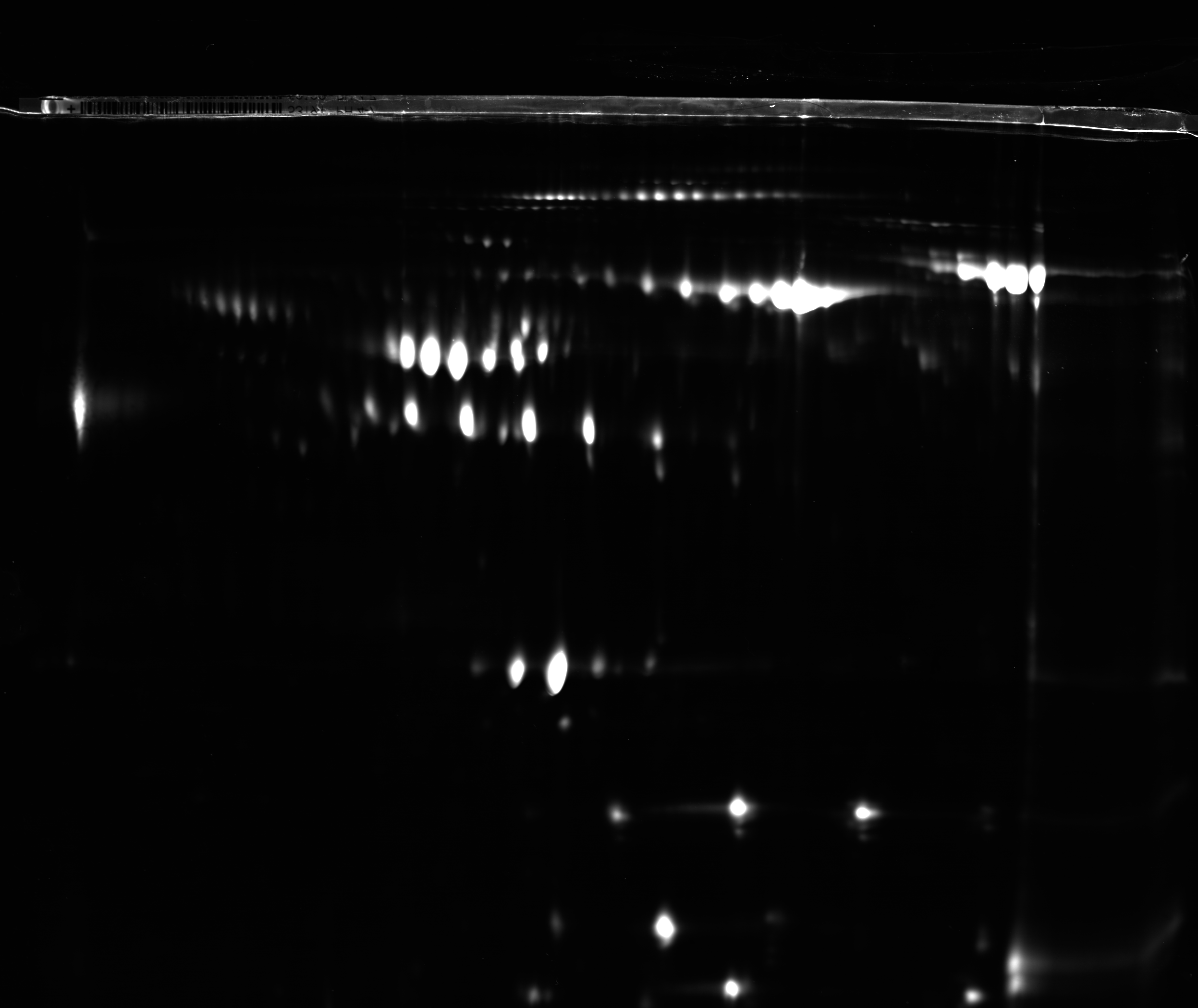

Supplement: Supplementary file 1 [file proteomes-13-00032-s001.zip › GELS/GEL6-CY3_PUB_254.bmp]

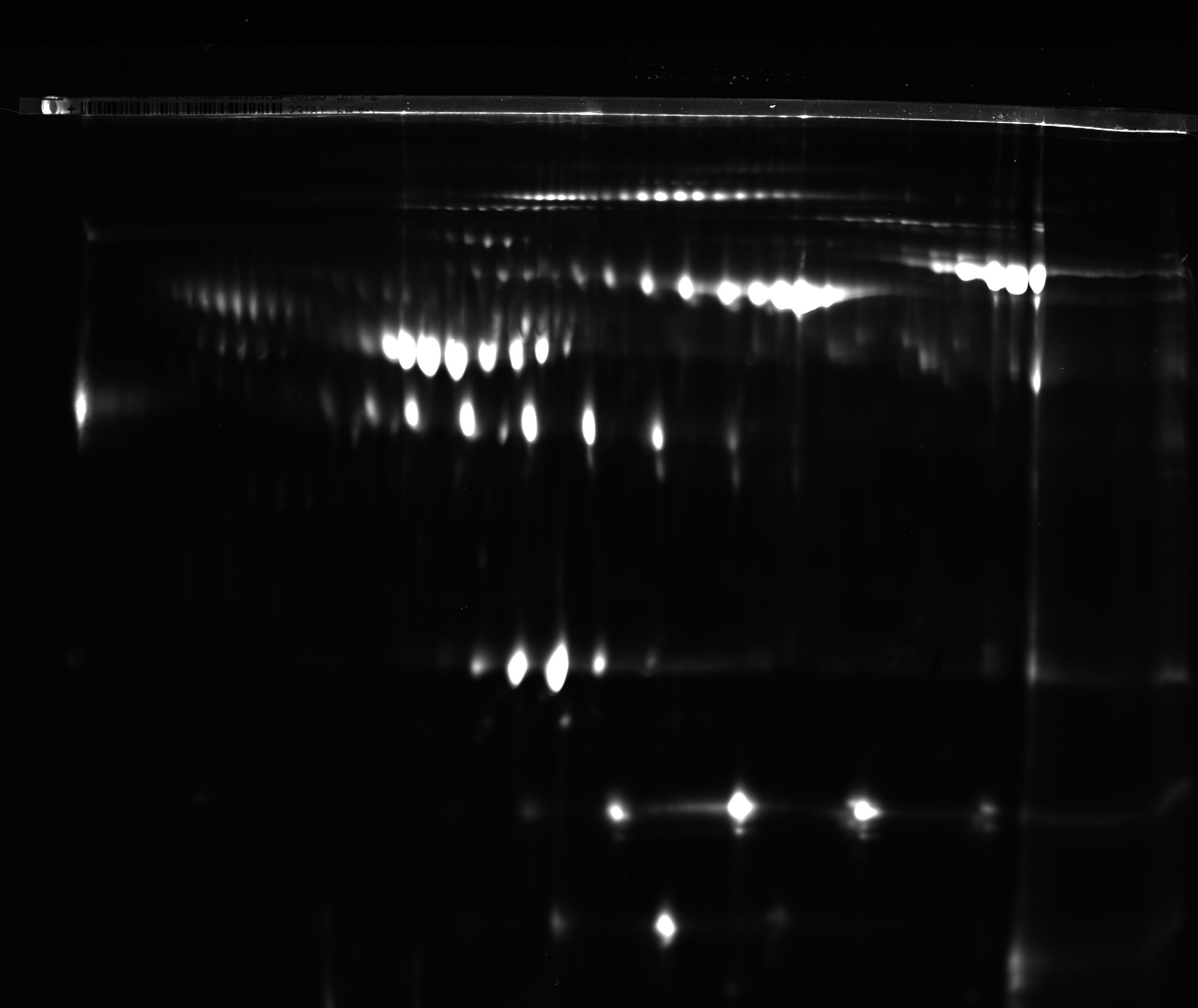

Supplement: Supplementary file 1 [file proteomes-13-00032-s001.zip › GELS/GEL6-CY5_PUB_254_PUB_254.bmp]

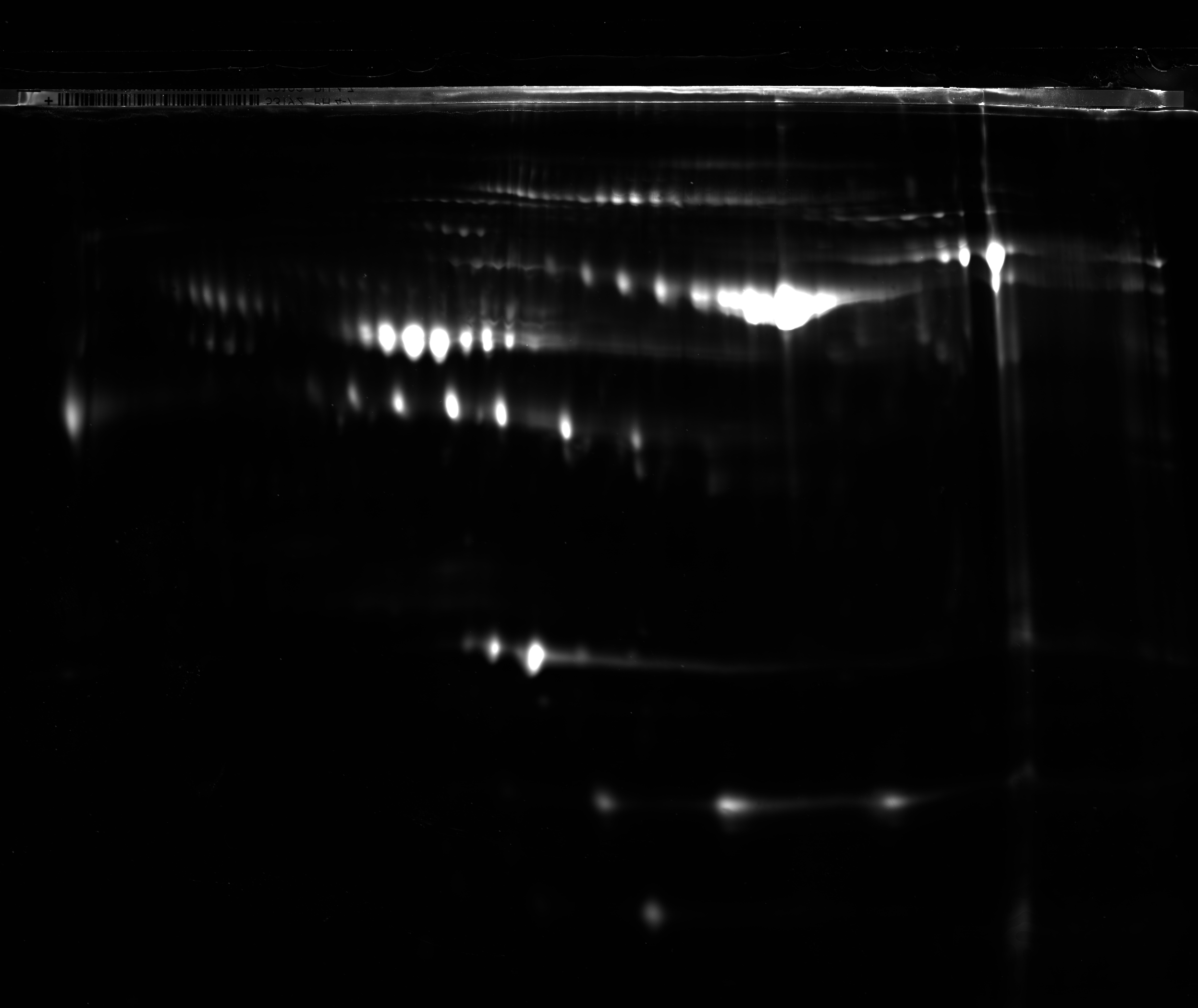

Supplement: Supplementary file 1 [file proteomes-13-00032-s001.zip › GELS/GEL7-CY3_PUB_254.bmp]

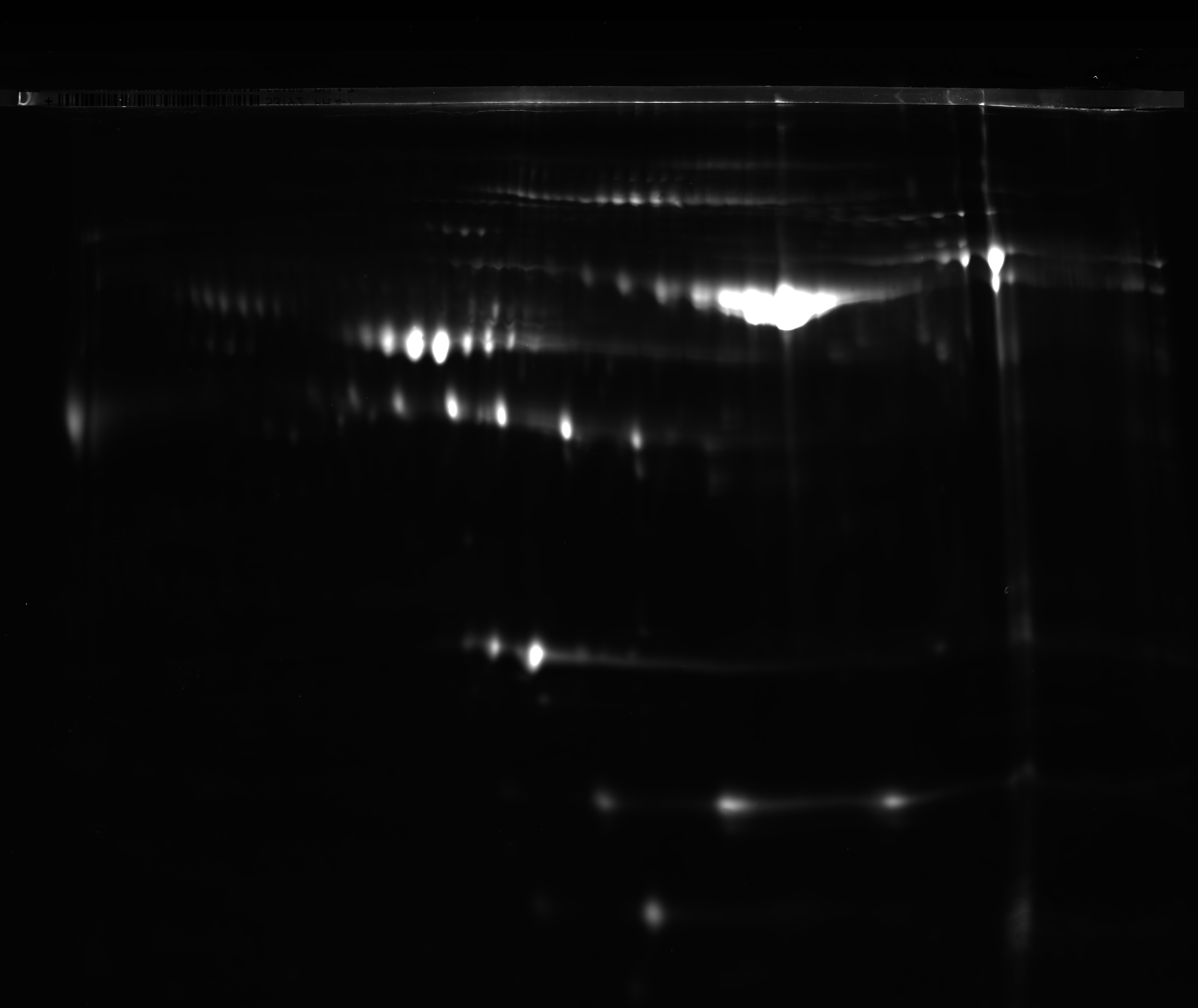

Supplement: Supplementary file 1 [file proteomes-13-00032-s001.zip › GELS/GEL7-CY5_PUB_254.bmp]

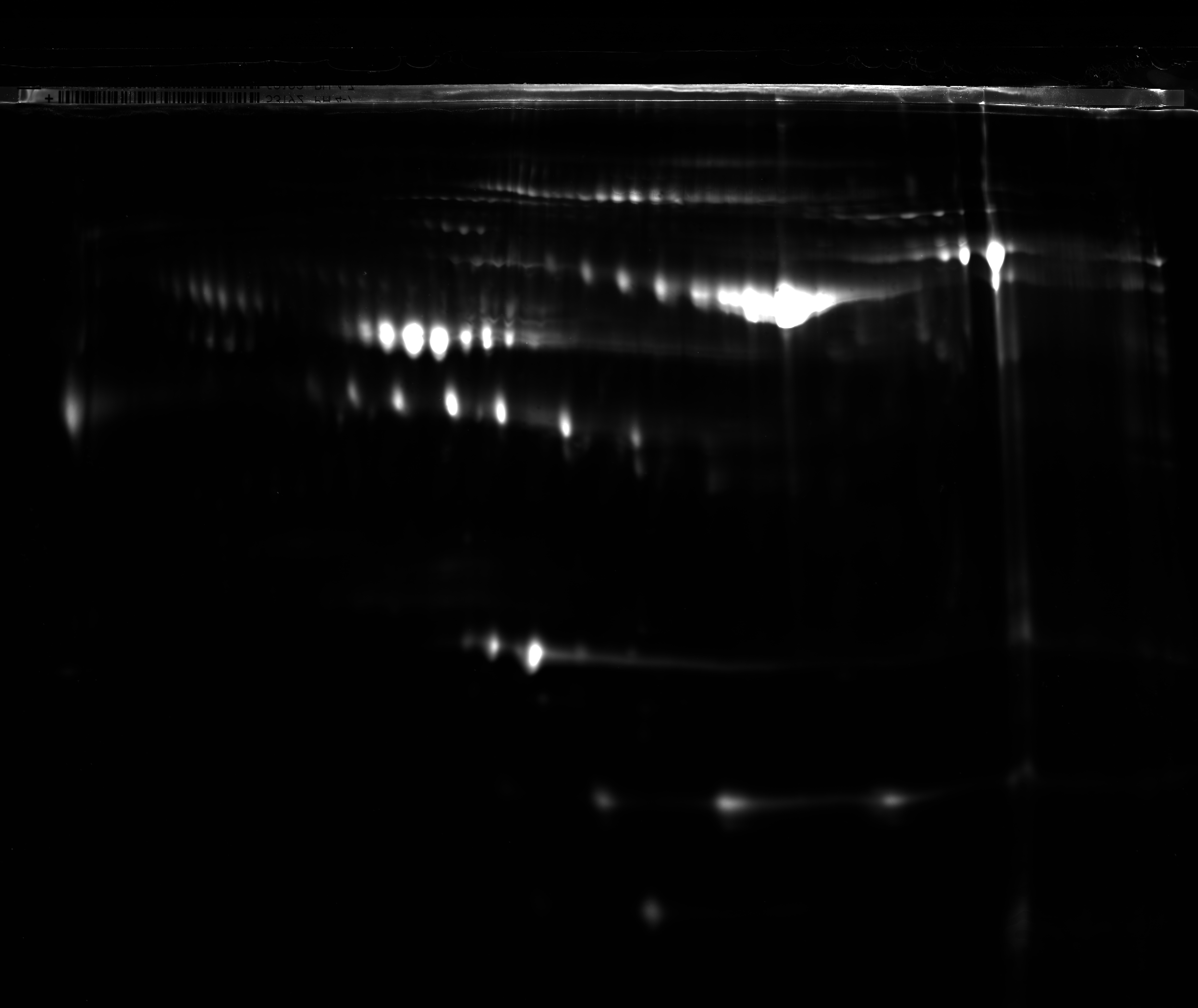

Supplement: Supplementary file 1 [file proteomes-13-00032-s001.zip › GELS/GEL8-CY3_PUB_254.bmp]

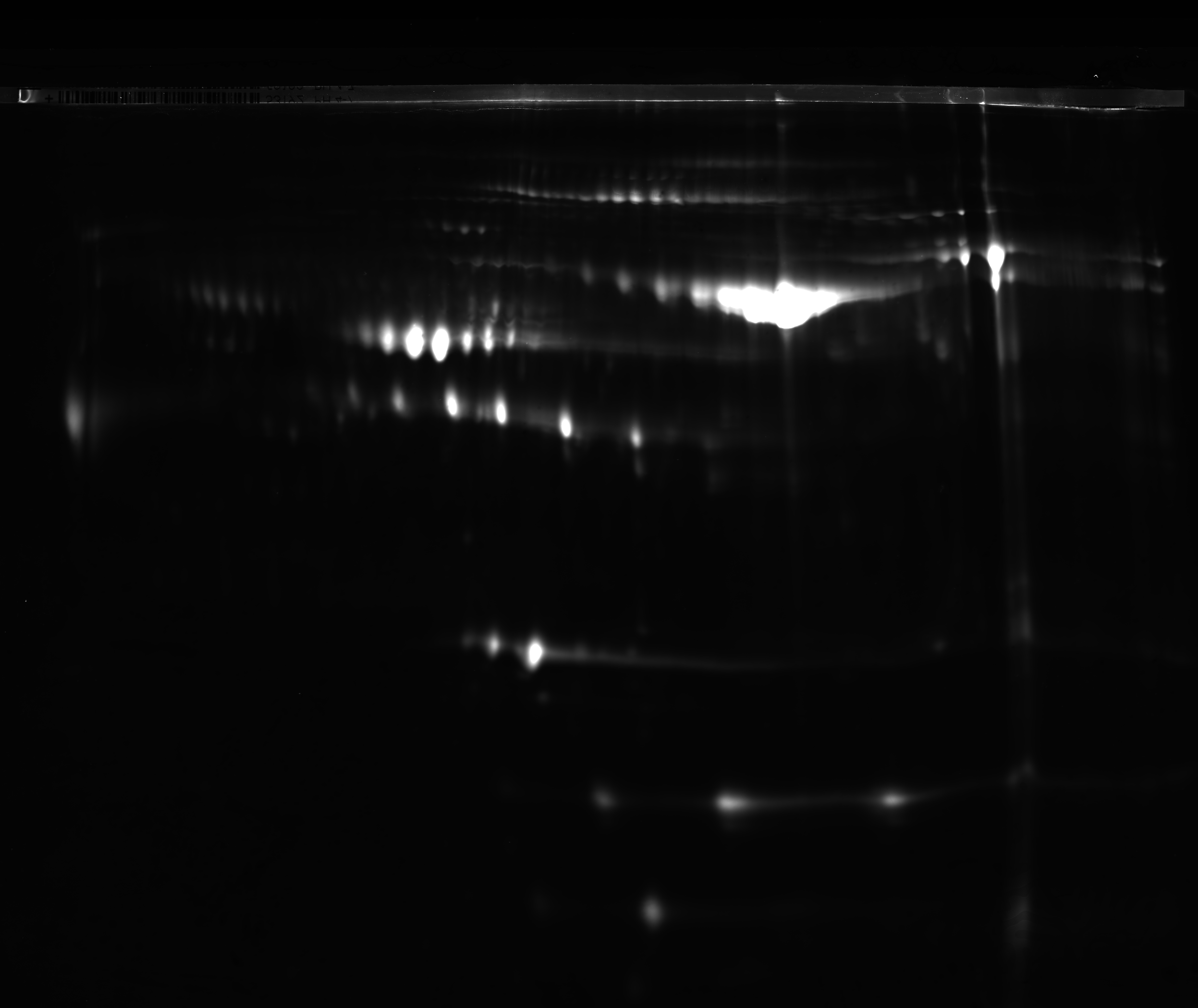

Supplement: Supplementary file 1 [file proteomes-13-00032-s001.zip › GELS/GEL8-CY5_PUB_254.bmp]

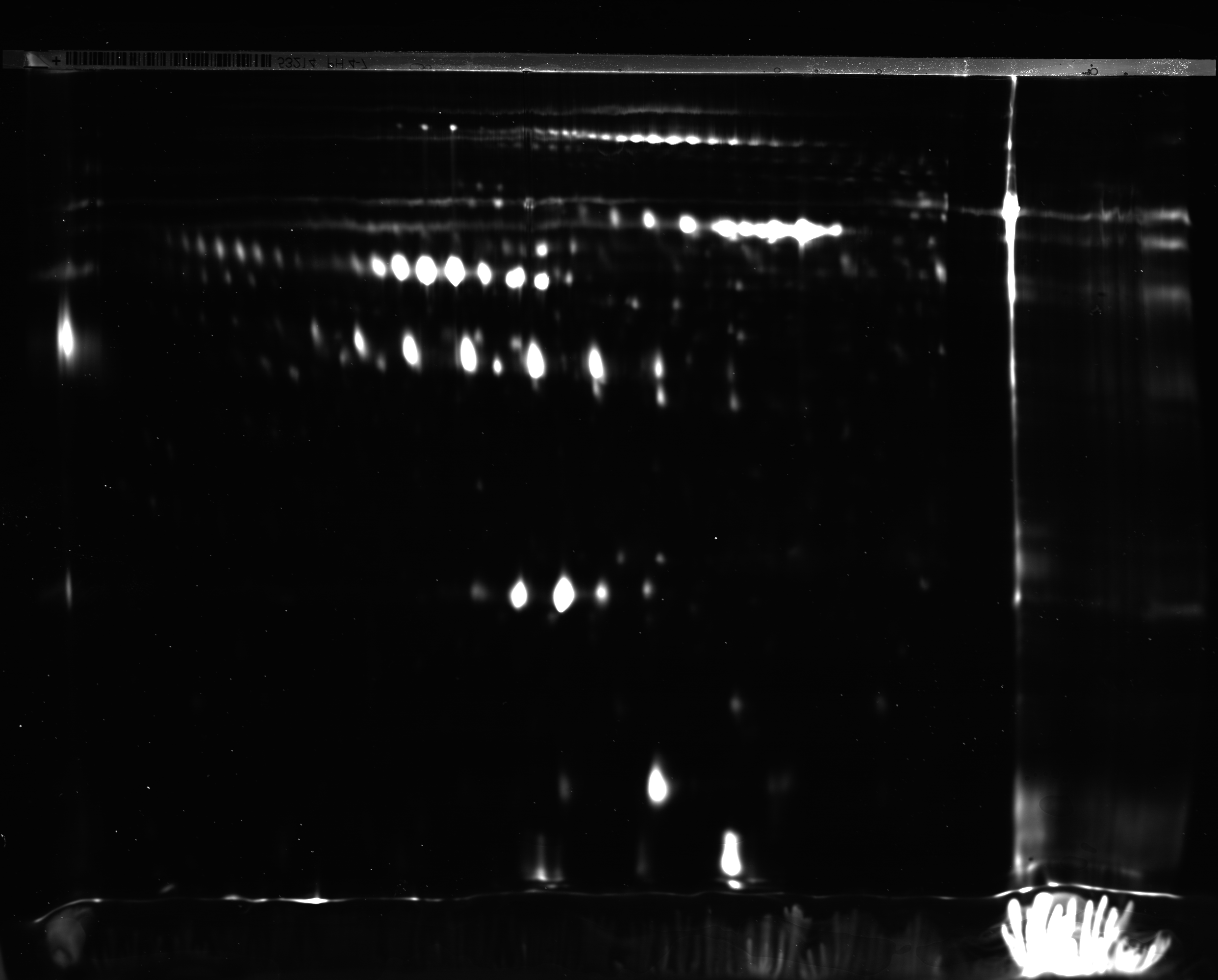

Supplement: Supplementary file 1 [file proteomes-13-00032-s001.zip › GELS/GEL9-CY3_PUB_254.bmp]

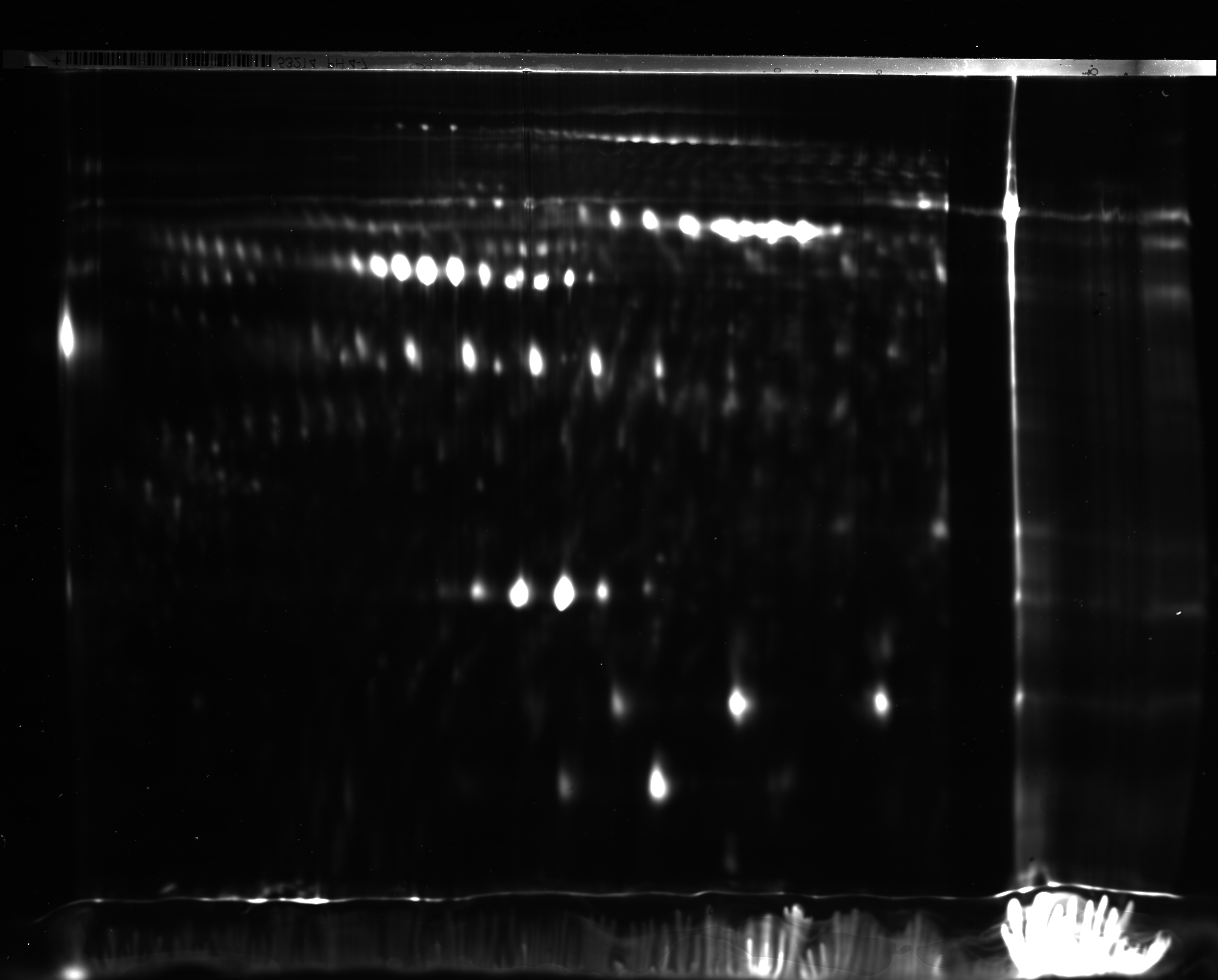

Supplement: Supplementary file 1 [file proteomes-13-00032-s001.zip › GELS/GEL9-CY5_PUB_254.bmp]
